# Transcriptome Profiles of Carcinoma-in-Situ and Invasive Non-Small Cell Lung Cancer as Revealed by SAGE

**DOI:** 10.1371/journal.pone.0009162

**Published:** 2010-02-11

**Authors:** Kim M. Lonergan, Raj Chari, Bradley P. Coe, Ian M. Wilson, Ming-Sound Tsao, Raymond T. Ng, Calum MacAulay, Stephen Lam, Wan L. Lam

**Affiliations:** 1 Genetics Unit, Integrative Oncology, British Columbia Cancer Research Centre, Vancouver, British Columbia, Canada; 2 Imaging Unit, Integrative Oncology, British Columbia Cancer Research Centre, Vancouver, British Columbia, Canada; 3 Ontario Cancer Institute, Princess Margaret Hospital, Toronto, Ontario, Canada; 4 Computer Science, University of British Columbia, Vancouver, British Columbia, Canada; National Cancer Institute, United States of America

## Abstract

**Background:**

Non-small cell lung cancer (NSCLC) presents as a progressive disease spanning precancerous, preinvasive, locally invasive, and metastatic lesions. Identification of biological pathways reflective of these progressive stages, and aberrantly expressed genes associated with these pathways, would conceivably enhance therapeutic approaches to this devastating disease.

**Methodology/Principal Findings:**

Through the construction and analysis of SAGE libraries, we have determined transcriptome profiles for preinvasive carcinoma-in-situ (CIS) and invasive squamous cell carcinoma (SCC) of the lung, and compared these with expression profiles generated from both bronchial epithelium, and precancerous metaplastic and dysplastic lesions using *Ingenuity Pathway Analysis*. Expression of genes associated with epidermal development, and loss of expression of genes associated with mucociliary biology, are predominant features of CIS, largely shared with precancerous lesions. Additionally, expression of genes associated with xenobiotic metabolism/detoxification is a notable feature of CIS, and is largely maintained in invasive cancer. Genes related to tissue fibrosis and acute phase immune response are characteristic of the invasive SCC phenotype. Moreover, the data presented here suggests that tissue remodeling/fibrosis is initiated at the early stages of CIS. Additionally, this study indicates that alteration in copy-number status represents a plausible mechanism for differential gene expression in CIS and invasive SCC.

**Conclusions/Significance:**

This study is the first report of large-scale expression profiling of CIS of the lung. Unbiased expression profiling of these preinvasive and invasive lesions provides a platform for further investigations into the molecular genetic events relevant to early stages of squamous NSCLC development. Additionally, up-regulated genes detected at extreme differences between CIS and invasive cancer may have potential to serve as biomarkers for early detection.

## Introduction

Lung cancer is estimated to inflict the highest cancer mortality rate in the United States in 2009 [1]. Molecular-targeted therapies directed towards signal transduction pathways active in cellular proliferation, differentiation, apoptosis, and angiogenesis, have been used in the treatment of non-small cell lung cancer (NSCLC), but with varied and often disappointing results [2], [3], [4], [5], [6]. As the simultaneous targeting of multiple signaling pathways has shown improvements in clinical response [6], further knowledge of biological pathways involved in lung cancer development, along with identification of aberrantly expressed genes within these pathways, would be expected to facilitate development of novel therapeutic intervention [7], [8].

**Figure 1:**
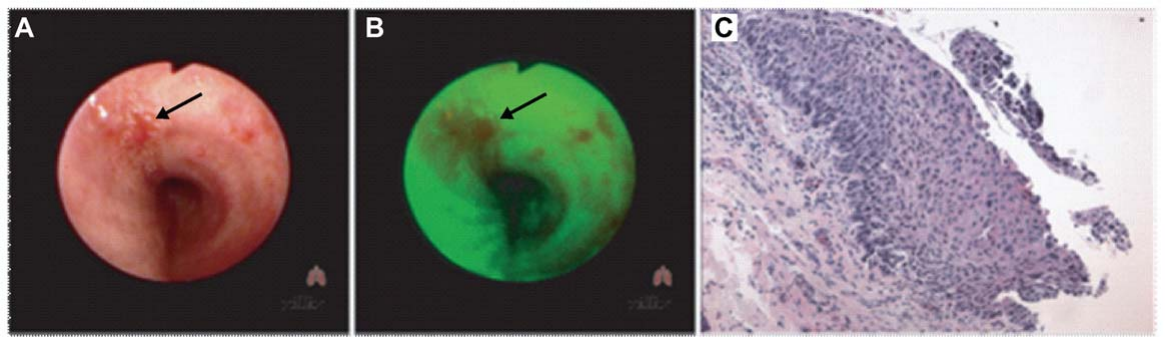
Detection of carcinoma-in-situ bronchial lesions. Bronchoscopy using **A**. white light for detection of CIS lesions (indicated by arrow), or **B**. LIFE (lung-imagine fluorescent endoscopy) for detection of CIS lesions (indicated by arrow). **C**. Histological section identifying a CIS lesion within the bronchial epithelium, typified by extensive squamous stratification.

Carcinoma-in-situ (CIS) of the lung, are preinvasive lesions of squamous NSCLC, frequently associated with histological, cytological, and genetic aberrations, and progression to invasive cancer typically ensues [9], [10], [11], [12]. As these minute lesions are optimally visible in the central airways by fluorescent bronchoscopy or LIFE (lung-imaging fluorescent endoscopy) [13], [14], experimental and clinical studies are rare (**Figure 1**). Although many expression profiling studies have been reported for advanced stage lung tumors [15], [16], early stage (CIS and locally invasive) lesions remain largely unexplored. Molecular genetic analysis of preinvasive lesions, free from background noise associated with commonly studied advanced tumors, is essential to the identification of key genes and corresponding molecular pathways underlying early events in neoplastic transformation and cancer development. An understanding of these early aberrations is essential for prompt therapeutic intervention of this devastating disease.

Large-scale gene expression studies frequently employ microarray technologies. Recent studies emphasize the value of tailor-made arrays, with target selection based upon prior knowledge of gene expression, to more accurately reflect the transcriptome of the tissue of interest; specifically applied to the study of NSCLC [17]. SAGE (serial analysis of gene expression) offers a sequence-based, non-biased approach to comprehensive transcriptome analysis, with no prior knowledge of expression required [18]. Moreover, information gained from SAGE analysis potentially contributes to design of more appropriate microarrays for focused research and diagnostic purposes.

In a previous study, we described the transcriptomes of smoke-damaged bronchial epithelium and lung parenchyma by way of SAGE [19]. Here we extend our analysis to the unexplored preinvasive stage in lung cancer development, and present the first report of large-scale expression profiling of carcinoma-in-situ of the lung. Additionally, we describe transcriptomes for invasive squamous cell carcinoma (SCC), and precancerous metaplastic and dysplastic (PC) lesions, derived from the construction and analysis of multiple SAGE libraries, culminating in greater than 22 megabases of sequence information. Through the utilization of *Ingenuity Pathway Analysis*, we have identified genes associated with epidermal development and xenobiotic metabolism/detoxification as components of CIS lesions, and genes associated with immune response and tissue remodeling/fibrosis as components of invasive SCC. Additionally, we found genes associated with mucociliary differentiation to be down-regulated in both CIS and PC lesions. We discuss the potential relevance of these transcriptional aberrations to the early stages of lung cancer development, and present a view of the CIS transcriptome previously unknown.

## Materials and Methods

### Ethics statement

This study was approved by the University of British Columbia-British Columbia Cancer Agency Research Ethics Board (UBC-BCCA REB). Written consent was obtained from all subjects.

### Specimens

Bronchial epithelial (BE) brushing specimens were previously described [19]. Biopsy specimens included precancerous (PC) lesions (metaplasia, dysplasia), carcinoma-in-situ (CIS) lesions, and invasive squamous cell carcinoma (SCC) tumor tissue. PC and CIS specimens were obtained by autofluorescent bronchoscopy (Figure 1). All biopsies (except for those used in constructing libraries CIS-3, SCC-5, and SCC-6) were collected in RNA*later*® [Applied Biosystems (Ambion Inc., Canada)] and stored at −85°C. The biopsy used in construction of library CIS-3 was embedded in OCT media, and stored at −85°C. For construction of libraries SCC-5 and SCC-6, RNA was isolated from tumor tissue from eight individuals by guanidium isothiocyanate and phenol/chloroform extraction. All subjects contributing to this study were either former or current smokers (**Table 1**).

**Table 1:** Summary of patient demographics and library descriptions.

Library^1^	Gender	Age	Smoking status	Pathology	Useful tags sequenced	GEO Series Accession
BE-3^2^	M	68	former	NA	152,086	GSE5473
BE-4A&4B	M	69	former	NA	222,395	GSE3707
BE-5^2^	M	70	former	NA	158,288	GSE5473
BE-6	M	67	former	NA	91,571	GSE3707
BE-7	M	56	current	NA	81,309	GSE3707
BE8B	M	72	former	NA	81,799	GSE5473
BE-9^2^	M	68	former	NA	162,903	GSE5473
BE-10	M	65	former	NA	86,725	GSE3707
BE-11A	F	56	former	NA	89,622	GSE3707
BE-12	F	63	current	NA	88,186	GSE3707
BE-13^2^	F	63	current	NA	93,345	GSE5473
BE-14	F	63	former	NA	155,462	GSE3707
BE-15	M	72	former	NA	143,129	GSE3707
BE-16	F	71	former	NA	131,285	GSE3707
				BE total =	1,738,105	
CIS-1	M	61	former	in-situ	163,460	GSE7898^3^
CIS-2	M	68	former	in-situ	160,466	GSE7898
CIS-3	M	69	current	in-situ	201,617	GSE7898
CIS-4	M	70	former	in-situ	174,246	GSE7898
CIS-5	M	72	former	in-situ	211,034	GSE7898
				CIS total =	910,823	GSE7898
SCC-1	M	78	current	invasive	150,712	GSE7898
SCC-2	M	62	former	invasive	152,220	GSE7898
SCC-3	M	70	current	invasive	208,451	GSE7898
SCC-4	M	68	former	invasive	176,874	GSE7898
SCC-5	1M/3F	54, 64, 75, 84	former/current	invasive	152,786	GSE7898
SCC-6	4M	65, 69, 74, 77	former/current	invasive	150,233	GSE7898
				SCC total =	991,276	GSE7898
Met	M	51	current	metaplasia	202,340	GSE7898
Dys	M	71	former	dysplasia	155,185	GSE7898
			Precancer total =		357,525	

BE, bronchial epithelial; CIS, carcinoma in-situ; SCC, invasive squamous cell carcinoma; Met, metaplasia; Dys, dysplasia.

1 With the exception of SCC-5 and SCC-6, all libraries were constructed from single individuals; the former of which were constructed from two pools of four individuals each.

2 With deeper sequencing of previously reported libraries generated in previous studies [19], [37].

3 All libraries constructed in this study were submitted to GEO as a single series.

### SAGE library construction and sequence processing

With the exception of construction of libraries SCC-5 and SCC-6, each specimen (biopsy or bronchial brushing as previously described [19]) was retrieved from RNA*later*® (or OCT for library CIS-3), and homogenized in lysis/binding solution. For libraries SCC-5 and SCC-6, RNA was pooled from four specimens in equal amount, and ∼19 µg of total RNA were immersed in lysis/binding solution and used for construction of each library. The resultant lysates were used directly for SAGE library construction according to the MicroSAGE protocol, using *Nla* III as the anchoring enzyme and *Bsm* FI as the tagging enzyme (www.ncbi.gov/SAGE). This approach was shown to yield highly reproducible SAGE libraries [19]. On average, 10^5^ SAGE tags, excluding linker and duplicate ditags, were sequenced per library; all raw SAGE data has been deposited with GEO (Table 1). For normalization, tag counts were scaled to 10^6^ tags for each library, i.e. as tags per million (TPM).

### Cluster analysis

To evaluate the degree of similarity among the lung SAGE libraries generated in this study (two PC libraries, five CIS libraries, and six invasive SCC libraries) and those generated in a previous study (14 BE libraries, and two lung parenchyma libraries), cluster analysis was used. For this analysis, the 300 most abundant tags were retained from each library, yielding a merged list of 1128 unique tags. The data were then log_10_ transformed and clustered using *Genesis*, using an average-linkage algorithm and a Euclidean distance metric [20]. For tags with counts of zero, the data was not transformed, and was retained as zero.

### Differential expression analysis

Unless stated otherwise, differential gene expression was defined by a minimal three-fold difference (rounded to one decimal place) in average normalized tag counts (TPM) between any two datasets being compared. In addition, a minimal average normalized tag count of 40 TPM was required in the over-expressing dataset. Tag-to-gene mapping was according to the SAGE Genie database, September 17, 2009 version [21] (cgap.nci.nih.gov/SAGE). As the newest release of SAGE Genie does not automatically align to mitochondrial transcripts, we utilized a manual mapping approach to identify these transcripts.

### Data analysis

Datasets of differentially expressed genes were analyzed primarily through the use of *Ingenuity Pathway Analysis* (IPA), version 8.0 (Ingenuity® Systems, www.ingenuity.com). *Functional analysis of entire datasets* identified the biological functions and/or diseases that were most significant to the dataset. Genes from the dataset that were associated with biological functions and/or diseases in the Ingenuity Pathways Knowledge Base were considered for the analysis (IPA eligible mapped IDs). *Canonical pathway analysis of entire datasets* identified the pathways from the IPA library of canonical pathways that were most significant to the dataset, based upon genes within the dataset that were associated with a canonical pathway in the Ingenuity Pathways Knowledge Base. *Toxicity list analysis of entire datasets* identified the Tox lists from the IPA library of Tox Lists that were most significant to the dataset. Genes from the dataset that were associated with a List were considered for the analysis. For each of these analyses, the significance of the association between the genes in the dataset and the assigned biological function and/or disease/canonical pathway/toxicity list, was measured by Fischer's exact test to calculate a p-value to determine the probability that the association was explained by chance alone.

*My Pathways* is a graphical representation of the biological relationships between gene products, which are supported by at least one reference from the literature, from a textbook, or from canonical information stored in the Ingenuity Pathways Knowledge Base. Genes are displayed using various shapes that represent the functional class of the gene product as indicated in the legend within the specific figures.

### RNA isolation from clinical specimens

For quantitative RT-PCR analysis, RNA from matched tumor and normal lung parenchyma were collected from resected tissues. Briefly, multiple sections of each tumor were cut, with the first and last in a series stained with H&E for inspection by a lung pathologist. After confirming diagnosis and assessing tumor heterogeneity with pathology review, we captured those portions of the tumor having a minimum of 70% cancer cells. RNA was extracted from both microdissected tumor tissue and from associated normal tissue with the *RNeasy* kit (Qiagen, Mississauga, ON, Canada). In total, RNA from nine paired tumor and normal parenchyma samples was used for RT-PCR analysis. In addition, RNA was extracted as previously described [19], from six bronchial brushings representing three current and three former smokers, for quantitative RT-PCR analysis.

### RT-PCR analysis

For quantitative RT-PCR (qPCR), approximately 1 µg total RNA was converted into cDNA using the High-Capacity cDNA Archive kit (cat# 4322171, Applied Biosystems), and gene-specific quantitative PCR was performed using TaqMan Universal PCR Master Mix and TaqMan primers (cat# 4326708; Applied Biosystems), according to manufacturer's recommendation. Beta-actin was used as an endogenous control (primer product code 4352935E). Primer product codes for test genes were as follows: ECE2 (Hs00981189_g1), MAGEA9 (Hs00245619_s1), MAGEA11 (Hs00377815_m1), CLDN1 (Hs00221623_m1), CKS1B (Hs01029137_g1), POSTN (Hs00170815_m1), ARTN (Hs00754699_s1), SFRP2 (Hs00293258_m1), UBE2S (Hs00819350_m1), C19orf48 (Hs00364147_m1), FBXO27 (Hs00381091_m1), MCM2 (Hs00170472_m1), NTS (Hs00175048_m1), SLC6A8 (Hs00373917_g1), and SLC2A1 (Hs00197884_m1, Hs00892681_m1). The reactions were run on an iCycler iQ Real-Time PCR Detection System (Bio-Rad Laboratories (Canada) Ltd., Mississauga, ON, Canada). Differential expression was determined using the delta-delta CT method. For the tumor/normal parenchyma pairs, fold changes were calculated for each pair. When comparing the tumors to the brushings, fold changes were calculated comparing each tumor to the average expression of the six BE samples. The average tumor over normal parenchyma fold change and the average tumor over BE fold change are reported for each gene.

### Gene dosage determination

Carcinoma-in-situ specimens used for copy-number profiling were collected in 10% buffered formalin. Microdissection was performed on paraffin sections to obtain cancer cells. Typically greater than 20 serial sections were necessary to yield sufficient material. DNA was isolated from collected cells by proteinase K phenol/chloroform extraction as previously described [22]. Whole genome tiling path array CGH analysis was performed using SMRT array version 2 as previously described [23], [24]. This platform is suitable for profiling formalin fixed paraffin embedded material [22], [23], [25], [26], [27], [28], [29], [30]. Genome segmentation and copy number status was performed using aCGH-Smooth on array image data and visualized using SIGMA software [31], [32], [33], [34]. Loss array elements were assigned a value of −1, retained elements a value of 0, and gained elements a value of 1. Twenty CIS specimens were profiled in total, and a threshold for gene-specific copy number gain/loss was set at 20%. The 20% threshold was imposed in an effort to reduce the detection of spurious or random events due to background genomic instability inherent to the samples, and thereby selecting for those events which occur with some degree of regularity.

### Public microarray data comparisons

Microarray expression data for 53 primary squamous tumors was retrieved from the Lung Cancer Dataset at NCBI, GEO accession number GSE3141 [35]. Microarray expression data for 67 bronchial brushings retrieved from a mixed population of current and former smokers, was profiled internally. All microarray data was RMA normalized [36].

## Results and Discussion

SAGE offers an unbiased and comprehensive approach to expression profiling, limited only by the depth of sequencing chosen by the researcher, and offers an unprecedented opportunity for transcript discovery. This is in sharp contrast to microarray analysis, where expression profile and scope of analysis is predetermined by target design typically employing a limited number of the most commonly characterized genes [17]. In a previous study, we described the transcriptomes of smoke-damaged bronchial epithelium and lung parenchyma by way of SAGE [19]. Here we extend our analysis to the largely unexplored area of early-stage lung cancer development, and present the first report of large-scale gene expression profiling of carcinoma-in-situ (CIS) of the lung. An understanding of the molecular genetics governing the preinvasive stages is critical to facilitate early detection and immediate therapeutic intervention before progression to invasive cancer ensues. In the current study, we present a comparative analyses, with emphasis on genes over-expressed in CIS and invasive cancer transcriptomes, relative to non-cancerous transcriptomes of the lung including bronchial epithelium (BE), and precancerous lesions (PC: squamous metaplasia and dysplasia).

Twenty-seven lung SAGE libraries comprised of 3,997,729 total sequence tags (∼40 megabases of high quality DNA sequence) were analyzed in this study. Normal lung is represented by 14 bronchial epithelial libraries (BE-1 through BE-14) [19], [37]. Precancer stage is represented by two libraries derived from squamous metaplasia (Met) and squamous dysplasia (Dys). Squamous cell carcinoma of the lung is represented by five carcinoma in-situ libraries (CIS-1 through CIS-5), and six invasive carcinoma libraries (SCC-1 through SCC-6) (detailed in **Table 1** and **Table 2**). (It is noted that specimens comprising the BE, PC, CIS, and SCC datasets were from a mixed population of current and former smokers.) This data has identified greater than 129,000 unique sequence tags/potential transcripts in CIS lesions, and nearly 140,000 unique sequence tags/potential transcripts in invasive squamous NSCLC.

**Table 2:** Summary of SAGE libraries generated and tags sequenced.

	BE^3^	Precancer	CIS	SCC	Total
Libraries	14	2	5	6	27
Tags Sequenced	1,738,105	357,525	910,823	991,276	3,997,729
Unique tags (UT)^1^	177,713	70,043	129,683	139,843	304,568
UTs excl. singleton^2^	76,471	24,661	49,054	52,017	150,331

BE, bronchial epithelial; Precancer (squamous metaplasia, squamous dysplasia); CIS, carcinoma-in-situ; SCC, invasive squamous cell carcinoma.

1 Unique tags are defined by the 10 nucleotide long sequence, and represent the maximum number of unique transcripts within the respective SAGE dataset.

2 Exclusion of singletons; singleton is defined as sequence tags having a raw tag count of one within an individual dataset (comprised of multiple libraries as indicated).

3 BE libraries were generated in previous studies [19], [37].

### Analysis of the top 300 most abundant tags in BE, CIS and SCC SAGE datasets

#### Cluster analysis

**Figure 2:**
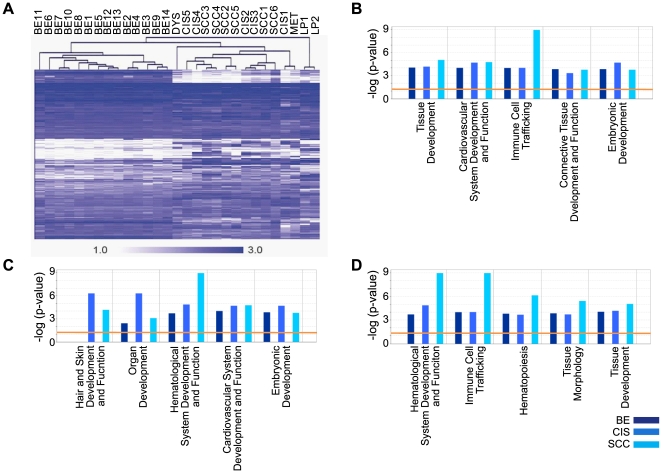
Analysis of the top 300 most abundant tags from the BE, CIS, and invasive cancer datasets. **A.** Cluster analysis of lung SAGE libraries. All SAGE libraries from this study, including five carcinoma in-situ libraries (CIS-1 through CIS-5), six invasive squamous cell carcinoma libraries (SCC-1 through SCC-6), one squamous metaplasia library (Met), and one squamous dysplasia library (Dys), as well as 14 bronchial epithelial libraries (BE-1 through BE-14), and two normal lung parenchyma SAGE libraries (LP-1, LP-2; accession GSE3708) generated in a previous study [19], [37], were analyzed by cluster analysis using an average-linkage algorithm. The top 300 most abundant tags were retained from each library, and analysis was based on 1128 unique tags in total. In the dendrogram, branch length represents distance. **B–D.** IPA functional analysis of the most abundant genes in the BE, CIS, and invasive cancer datasets. Tag-to-gene mappings for the top 300 most abundant tags from the BE, CIS, and SCC datasets, were used for IPA core analysis, consisting of 220, 231, and 233 IPA eligible mapped IDs, respectively. The three sets of data were displayed together using IPA core comparisons, and the five most significant functions within *Physiological System Development and Function* are shown for each of the three datasets. The data in B is sorted according to highest significance in BE, the data in C is sorted according to highest significance in CIS, and the data in D is sorted according to highest significance in invasive SCC. The orange line indicates the threshold limit of significance, preset at a p-value of 0.05. For a complete listing of the tags/mapped IDs used for this analysis see Table S1.

Cluster analysis yielded anticipated grouping of SAGE libraries, attesting to sample quality. For this analysis, the 300 most abundant tags were retained from each library, yielding a merged list of 1128 unique tags. Average linkage clustering analysis based on the 1128 most abundant SAGE tags, reveals that all cancer libraries (both CIS and invasive SCC) cluster together, and separately from the BE libraries (**Figure 2A**). We note some clustering of the invasive SCC libraries (four out of six). Similar clustering is observed when using the top 500 or top 1000 unique tags per library (data not shown).

#### Ingenuity pathway analysis

To characterize and compare the bronchial epithelial and cancer transcriptomes, we computated average normalized tag counts for BE (14 libraries), CIS (5 libraries), and invasive SCC (6 libraries) datasets, and subsequently selected the most abundant 300 unique tags from each dataset for analysis (**Table S1**). Tags mapping to mitochondrial-encoded genes and ribosomal protein genes, were found at similar frequencies within the top 300 most abundant tags, across all three datasets of BE, CIS, and SCC, at ∼8% and ∼18%, respectively. We used the core analysis component of *Ingenuity Pathway Analysis* (IPA) to categorize these genes according to biological functions. Only those molecules having at least one functional annotation in the IPA Knowledge Base qualify for analysis, and included 220 (BE), 231 (CIS), and 233 (SCC) IPA eligible genes. These analyses reveal that genes within the category of *Hair and Skin Development and Function* are highly expressed in the CIS transcriptome relative to both BE and SCC, and genes within the categories of *Hematological System Development and Function* and *Immune Cell Trafficking* are highly expressed in the SCC transcriptome relative to both BE and CIS. Hence, high expression of genes associated with epidermal development is identified here as a characteristic feature of the CIS transcriptome, and high expression of genes associated with cellular movement as a characteristic feature of the SCC transcriptome. See **Figure 2B–D** for a summary of these analyses.

A notable feature of both the CIS and SCC datasets is the abundance of tags mapping to the constant region of immunoglobulin heavy chains. In fact, the SAGE tag for IGHG1 is the most abundant tag in both the CIS and the invasive cancer datasets (Table S1). Although it is possible that expression of these immunoglobulin chains originates from infiltrating lymphoid tissue and surrounding stroma, previous studies have demonstrated expression of heavy chain constant and variable regions of immunoglobulins in breast cancer epithelial cells [38], [39], [40], and expression of IgG heavy and light chains in various epithelial cancer cells including lung SCC [41]. These immunoglobulin chains may represent cancer cell autoantibodies, and stimulate growth in an autocrine/paracrine fashion [40]. Intriguingly, the abundance of tags mapping to immunoglobulin heavy chain transcripts in CIS preinvasive lesions of the lung, in contrast to the relatively low detection in both BE and precancerous metaplastic and dysplastic lesions (detailed in subsequent tables), suggests that up-regulation of these transcripts may have relevance to initiation of NSCLC.

### Gene expression changes common to carcinoma-in-situ and precancerous lesions

**Figure 3:**
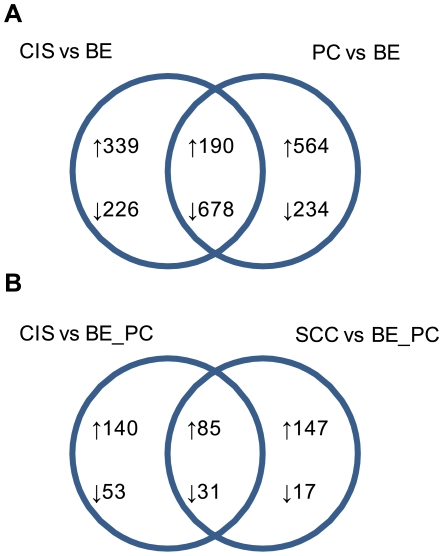
Venn diagrams of differentially expressed genes discussed in this manuscript. Criteria for differential gene expression was defined as a minimal three-fold difference in normalized mean tag counts, and with a minimal mean tag abundance of 40 TPM in the over-expressing datasets. Up-arrows indicate up-regulated gene expression changes; down-arrows indicate down-regulated gene expression changes; numerical values refer to the number of differentially expressed tags. Areas of interception reflect gene expression changes in common between the two datasets. **A.** Expression changes in carcinoma-in-situ and precancerous lesions relative to BE. **B.** Expression changes in the cancer datasets relative to both bronchial epithelium and precancerous datasets. BE: bronchial epithelial; PC: precancer; CIS: carcinoma-in-situ; SCC: invasive squamous cell carcinoma.

Transition from a healthy bronchial epithelium to invasive cancer is thought to proceed via progression of histological and genetic abnormalities: BE to PC to CIS to SCC, where PC represents precancerous lesions (squamous metaplasia and dysplasia). Squamous metaplasia is a transient component of normal wound healing of the bronchial epithelium, and typically resolves to a re-differentiated epithelium composed of pseudostratified ciliated and secretory cells, restoring bronchial function [42]. (Use of the term PC here does not imply an obligatory progression to cancer, but rather refers to lesions/abnormalities that despite infrequent progression to cancer, are considered as precursors to cancer.) Conversely, CIS lesions demonstrate a low regression frequency with a high incidence of progression to invasive cancer [43], [44] and are characterized by a more extensive stratification of squamous cell types compared to PC [9], [11], [12]. By identifying gene expression changes common to both PC and CIS relative to BE, we focus on those genetic events which occur early and persist through to CIS. In accordance with our selection criteria (minimal three-fold difference in average normalized tag abundance; minimal average normalized tag abundance of 40 TPM in the over-expressing dataset), 868 SAGE tags were found to be similarly differentially expressed in PC and CIS relative to BE, consisting of 190 up-regulated tags, and 678 down-regulated tags (**Figure 3A**).

#### Up-regulated expression changes

Approximately 35% of the tags up-regulated in CIS relative to BE (190 out of 529 tags) were found to be commonly up-regulated in PC lesions, and approximately 25% of tags up-regulated in PC lesions relative to BE (190 out of 754 tags) were found to be similarly up-regulated in CIS (Figure 3A). See **Table S2** for a description of these tags up-regulated in both PC and CIS. IPA functional analysis (based on 143 eligible mapped IDs) indicates that roughly 35% of these commonly up-regulated genes are associated with epidermal development and associated disorders, as described in **Table 3**. In addition to those genes described in Table 3, a review of the literature identified other genes within the PC/CIS up-regulated dataset to be associated with epidermal development, including SBSN, CNFN, CRCT1, additional members of the small proline-rich family of proteins (SPRR2E and SPRR3), and additional members of the S100A family of calcium-binding proteins. Many of these genes are encoded either within the epidermal differentiation complex (EDC) locus on 1q21 [45], [46] or within a conserved locus on 19q13 [47], and specify components of the cornified cell envelope, a structure that provides barrier protection to epidermis and internal epithelium in response to insult or injury [48], [49].

**Table 3:** Genes associated with epidermal development in the CIS_PC over BE dataset by IPA functional analysis.

Function Annotation	p-value	Molecules^3^	# Molecules^4^
**Category: Dermatological Diseases and Conditions** 1			
dermatological disorder	4.18E-17	COL1A1, COL1A2, COL3A1, COL6A1, COL7A1, DEFB103A, DEFB4, DSG1, DSG3, DSP, FYN, GJA1, HIF1A, IGHG1, IGL@, ITGA6, JUP, KRT5, KRT14, KRT17, KRT6A, KRT6B, LGALS1, LMNA, LTBP2, MMP1, PKP1, S100A7, S100A8, SELL, SFN, TP63, TUBA1C, TUBA4A, TYMS	35
dermatological disorder of mammalia	2.98E-03	DSG1, DSG3, IGHG1, MMP1, SELL	5
epidermolysis bullosa	3.66E-09	COL7A1, DSP, ITGA6, KRT5, KRT14, MMP1	6
recessive epidermolysis bullosa dystrophica	8.12E-05	COL7A1, MMP1	2
burn	1.04E-04	COL1A1, COL1A2, COL3A1, COL6A1, COL7A1	5
Ehlers-Danlos syndrome	1.94E-04	COL1A1, COL1A2, COL3A1	3
skin cancer	4.53E-04	CD44, FYN, HIF1A, KLK6, MCL1, MMP2, TUBA1C, TUBA4A, TYMS	9
epidermolysis bullosa simplex	4.81E-04	KRT5, KRT14	2
skin tumor	1.62E-03	CD44, FYN, HIF1A, MCL1, MMP2, TUBA1C, TUBA4A, TYMS	8
disease of skin	2.04E-03	CAV1, DSG3, KRT14, MMP1, SELL	5
acanthosis	2.80E-03	DSG3, MMP1	2
psoriasis	3.90E-03	DEFB103A, DEFB4, IGHG1, S100A7	4
pemphigus of mice	4.23E-03	DSG1, DSG3	2
**Category: Hair and skin Development and Function** 2			
development of epidermis	7.70E-13	COL1A1, COL7A1, DSP, EMP1, EVPL, FABP5, KRT5, KRT14, KRT17, S100A7, SPRR1A, SPRR1B, TP63	13
development of skin	1.07E-03	COL1A1, COL3A1, SFN, TP63	4
differentiation of keratinocytes	1.12E-08	CSTA, DSP, EVPL, FABP5, IVL, SFN, SPRR1A, SPRR1B, TP63	9
cell movement of keratinocytes	4.23E-03	JUP, TP63	2
proliferation of epidermal cells	6.78E-03	JUP, KLK6, SFN, TP63	4

1 IPA Diseases and Disorders.

2 IPA Physiological System Development and Function.

3 See Table S2 for corresponding tag abundance.

4 47 unique genes were identified out of 143 IPA eligible mapped IDs.

**Figure 4:**
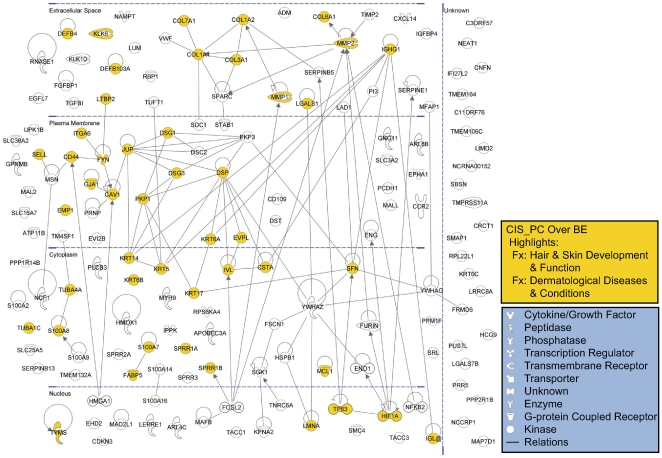
IPA pathway graphical representation for the CIS_PC over BE dataset of up-regulated genes. 155 genes (IPA mapped IDs) are represented out of 190 SAGE tags up-regulated in both CIS and PC relative to BE. (See Table S2 for tag data.) Gene products are positioned according to subcellular localization. Only direct connections (i.e., direct physical contact between two molecules) among the individual gene products are shown for clarity of presentation; lines indicate protein-protein binding interactions, and arrows refer to “acts on” interactions such as proteolysis, expression, and protein-DNA/RNA interactions. Genes associated with epidermal development (see Table 3) are highlighted.

IPA pathway graphical representation of the genes commonly up-regulated in CIS and PC relative to BE, is presented in **Figure 4**. Considering the molecular interactions identified by IPA, functional associations among desmosomal cadherins and catenins are prominent within the PC/CIS up-regulated dataset. Desmosomes are intercellular adhesion junctions that provide mechanical integrity to the epithelium, and studies indicate that desmosomal cadherins modulate keratinocyte differentiation and epidermal morphogenesis [50]. This analysis also suggests that a signaling cascade mediated by members of the 14-3-3 family of proteins, may be active here. 14-3-3 sigma (SFN) mediates keratinocyte differentiation and stratification of epidermis [51]. The pathway diagram also suggests that specific aspects of keratinocyte terminal differentiation may be mediated by the AP-1 transcription factor FOSL2, which may have an additional role in extracellular matrix remodeling. Indeed, FOSL2 has been identified as a mediator of pulmonary fibrosis [52]. A consideration of genes associated with the transcription factor HIF1A in the PC/CIS lesions, is suggestive of a remodeling/profibrotic response to hypoxic growth conditions [53], [54], [55]. Indeed, a functional link between hypoxia and fibrosis is documented in the literature [56], [57], [58]. Expression of other genes identified here, such as NCF1 (the regulatory subunit of NADPH oxidase), and the heme catabolic enzyme HMOX1, is also indicative of an oxygen-related stress response and tissue remodeling/fibrosis [59], [60], [61], [62]. It is noted that additional IPA analysis identified *Hepatic Fibrosis/Hepatic Stellate Cell Activation* and *14-3-3-mediated Signaling* as significant categories within *Canonical Pathways*, and identified *Hepatic Fibrosis* as the most significant category within *Toxicity Lists* (data not shown).

Additional analysis by Gene Ontology using the *GATHER* annotation tool [63], similarly identified epidermal development as a prominent component of the transcriptome of commonly up-regulated genes in CIS and PC lesions, relative to BE (**Figure S1A, Table S3**).

#### Down-regulated expression changes

The majority of tags down-regulated in CIS relative to BE, were found to be commonly down-regulated in PC lesions (678 tags out of 904 tags), and vice versa (678 tags out of 912 tags down-regulated in PC relative to BE) (Figure 3A). See **Table S4** for a description of these tags down-regulated in both PC and CIS. IPA functional analysis (based on 347 eligible mapped IDs) identified *Cellular Assembly and Organization* and *Embryonic Development* as the two most significant functional categories (p-values 1.29E-05–4.67E-02 and 1.30E-05–4.32E-02, respectively) for this dataset of commonly down-regulated genes (data not shown). Within the former, seven genes associated with biogenesis and formation of cilia were identified. These include dynein components of the cilium axoneme (DNAI2, DYNC2H1), FOXJ1 transcription factor and master regulator of motile ciliogenesis [64], intraflagellar transport proteins IFT172 and IFT88, kinesin family member KIF3A, and BBS5, a protein family member linked to Bardet-Biedl Syndrome and localized to ciliary basal bodies. Genes identified within the functional category of *Embryonic Development* are associated with patterning, specification of the midline axis, and formation of the neural tube. As these developmental processes have been linked to ciliary activity, specifically primary cilia-mediated Hedgehog signaling [65], [66], presumably this data overall reflects loss of the ciliated cell phenotype as common to PC and CIS lesions.

We also note at a lower significance, down-regulation of genes associated with DNA recombination and repair in CIS and PC lesions (p-value 1.16E-04-4.42E-02). These include DNA repair genes (CCNO and NEIL1, glycosylases associated with base-excision repair; cyclin-dependent kinase CDK2; glycoprotein CLU; p53-inducible ribosomal protein RPS27L associated with the G1 DNA damage checkpoint; helicase RUVBL2; TRIP13, a regulator of double-strand break repair and meiotic checkpoint control; antioxidant SOD1), genes associated with DNA modification (editing enzyme APOBEC3G; antioxidant CAT), and DNA catabolism (exoribonuclease XRN2), mediators of ATP hydrolysis (ATPIF1, MAPK1, N4BP2, RUVBL1, RUVBL2, TGM2), centriole duplication (AKAP9, CETN2), and folate receptor FOLR1.

IPA pathway graphical representation of genes commonly down-regulated in CIS and PC also highlights genes associated with ciliogenesis including axonemal components and centrosomal proteins, genes associated with goblet cell differentiation, and genes associated with epithelial cell polarization and ion transport (**Figure S2**). Down-regulation of these genes, as well as many others in the BE over PC_CIS dataset also associated with ciliogenesis but not identified as such by IPA, reflect a pronounced loss of mucociliary differentiation in both PC and CIS lesions, presumably accompanied by deficiency in clearance and defense of the airways [67]. For further description of the genes identified in Figure S2, see **Text S1**.

Additional analysis by Gene Ontology using the *GATHER* annotation tool [63], identified processes associated with cilia function such as gametogenesis and spermatogenesis, and microtubule-based processes, as prominent components of the transcriptome of commonly down-regulated genes in CIS and PC lesions, relative to BE (**Figure S1B**, Table S3).

### Identification of differentially expressed genes in carcinoma-in-situ and invasive cancer transcriptomes key to cancer development

By identifying genes differentially expressed between preinvasive and invasive stages of lung cancer development (CIS and SCC, respectively), relative to both non-cancerous bronchial epithelium and precancerous metaplasia/dysplasia lesions (BE and PC, respectively), we propose to identify expression changes instrumental to both initiation (CIS) and progression (SCC) of lung cancer. In accordance with our selection criteria (minimal three-fold difference in average normalized tag abundance; minimal average normalized tag abundance of 40 TPM in the over-expressing dataset), 309 SAGE tags were found to be differentially expressed in CIS relative to BE and PC, and 280 tags were differentially expressed in SCC relative to BE and PC, with 116 tags similarly differentially expressed (**Figure 3B**). It is noted that the stringent selection criteria imposed in this study for differential expression would preclude certain genes, although present in the SAGE datasets and relevant to cancer development, from further analysis (see example below). However, a high stringency within the selection process, typically lends greater confidence to the relevance of those genes identified as differentially expressed in the cancer datasets.

#### Up-regulated expression changes

**Figure 5:**
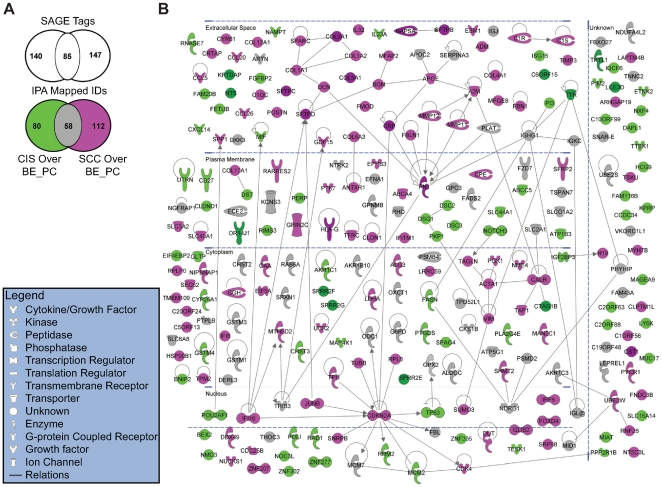
Genes up-regulated in the CIS and invasive SCC datasets relative to BE and PC. **A.** Venn diagram of up-regulated SAGE tags and corresponding IPA mapped IDs for the CIS and SCC datasets. (See Table S5 and Table S6 for description of up-regulated tags in the CIS and SCC datasets, respectively.) **B**. IPA pathway graphical representation for the CIS over BE_PC dataset (80 unique IDs displayed in green; 58 shared IDs displayed in gray), and the SCC over BE_PC dataset (112 unique IDs displayed in red; 58 shared IDs displayed in gray). Gene products are positioned according to subcellular localization. Only direct connections (i.e., direct physical contact between two molecules) among the individual gene products are shown for clarity of presentation; lines indicate protein-protein binding interactions, and arrows refer to “acts on” interactions such as proteolysis, expression, and protein-DNA/RNA interactions. Eleven genes were detected at levels 20-fold or greater in the CIS over BE_PC dataset relative to the invasive cancer dataset (indicated by dark green), and 10 genes were detected at levels 20-fold or greater in the SCC over BE_PC dataset relative to the CIS dataset (indicated by dark red).

We identified 225 SAGE tags to be over-expressed in CIS relative to both BE and PC (**Table S5**), and 232 tags to be over-expressed in invasive SCC relative to both BE and PC (**Table S6**). It is noted that greater than 35% of the over-expressed tags within the CIS dataset (85 tags) were commonly up-regulated in SCC (Figure 3B), suggesting that significant expression changes relating to advanced cancer have already occurred by the time a diagnosis of CIS has been made, in accordance with irreversibility of CIS lesions. Discrepancy between the number of up-regulated tags and the number of IPA mapped IDs within each dataset, indicates that a significant proportion of potentially up-regulated genes in early-stage lung cancer remain to be identified (**Figure 5A**). IPA pathway graphical representation for up-regulated tags with mapped IDs for the two cancer datasets, is presented in **Figure 5B**. A higher proportion of up-regulated gene products are localized to the extracellular space in the SCC dataset relative to the CIS dataset. Considering the molecular interactions identified by IPA, functional networks involving the cell surface/extracellular matrix adhesion protein FN1, and transcriptional/cell cycle regulator CDKN2A, highlight the SCC dataset. Up-regulation of FN1-interacting proteins associated with tissue remodeling/fibrosis, and FN1-interacting proteins associated with acute phase response, suggests a link between these processes in SCC. A link between acute phase response and tissue repair has been previously proposed [68]. Activation of a CDKN2A functional network associated with cellular senescence, may reflect a protective response of the involved organ to acute tissue injury [69]. No outstanding molecular interactions were apparent for the CIS up-regulated dataset. Differential expression for a subset of the up-regulated genes in Figure 5B was validated by real-time quantitative RT-PCR (**Table S7**).

We performed analysis by Gene Ontology using the GATHER annotation tool [63] for genes up-regulated in CIS and SCC relative to BE and PC (**Figure S3; Table S8**). This analysis identified fatty acid biosynthesis (described by a cluster of 5 genes) as a component of the CIS dataset. In agreement with IPA analysis described above, gene ontology analysis identified defense response (described by a cluster of 20 genes) as a notable component of the invasive cancer dataset. Additionally, skeletal development, response to wounding, anion transport, and carbohydrate catabolism were also identified by GATHER gene ontology analysis, as components of the invasive cancer dataset.

#### Epidermal development

Considering the notable expression of genes associated with epidermal development common to the PC and CIS datasets, we investigated whether functionally related genes are also enriched in the cancer datasets (CIS and SCC) relative to both BE and PC. The genes identified from this investigation using IPA functional analysis are described in **Table 4**. These data indicate that gene expression patterns reflective of epidermal development are not restricted to precancerous lesions, but rather also present as a component of CIS (∼15% of IPA eligible mapped IDs), and invasive cancer (∼26% of IPA eligible mapped IDs) apart from precancerous lesions.

**Table 4:** Up-regulated genes associated with epidermal development in CIS and invasive SCC according to IPA functional analysis.

Function Annotation	CIS/BE_PC^3^ p-value	SCC/BE_PC^4^ p-value	Molecules^5^	# Molecules^6^
**Category: Dermatological Diseases and Conditions** 1				
burn		*3.35E-08*	*COL12A1, COL17A1, COL1A1, COL1A2, COL3A1, COL4A1, COL5A1, COL6A3*	8
Ehlers-Danlos syndrome		*5.63E-06*	*COL1A1, COL1A2, COL3A1, COL5A1*	4
Ehlers-Danlos syndrome, type I		*2.77E-04*	*COL1A1, COL5A1*	2
psoriatic arthritis	**8.43E-05**		**CD27, IGHG1, IGKC, IL23A, MAP4K1**	5
psoriatic arthritis of humans	**1.37E-02**		**IGHG1**	1
malignant cutaneous melanoma		*5.51E-04*	*CDK4, CDKN2A*	2
dermatological disorder	**9.67E-04**	*5.55E-10*	*APOE, C1R, C1S, CCL5, CCL20,* **CD27**, *CDC25B, CDKN2A, CLDN1, COL12A1, COL17A1, COL1A1, COL1A2, COL3A1, COL4A1, COL5A1, COL6A3, CRP,* **DSG1**, *FN1, GDF15, * ***GPX2*** *, GRIN2C, * ***IGHG1, IGKC*** *, **IGL@*** ***,*** ** IL23A**, *JUNB,* **MAP4K1, ** ***ODC1,*** ** PERP, PKP1,** ***PSMB4,*** ** TP63**	34
dermatological disorder of mammalia	**3.86E-02**		**DSG1, IGHG1, IL23A**	3
blister	**2.49E-03**	*4.83E-03*	*COL17A1,* **DSG1**, ***PLAT***	3
blistering of epithelial tissue	**6.89E-03**		**PERP**	1
psoriasis	**1.30E-02**		**IGHG1, IL23A, ODC1**	3
fibrosis of dermis		*1.36E-03*	*CDK4, COL1A1*	2
hereditary angioedema		*1.36E-03*	*C1R, C1S*	2
hirsutism	**1.37E-02**		**ODC1**	1
metaplasia of squamous epithelium	**1.37E-02**		**TP63**	1
skin tumor	**2.61E-02**	*9.26E-03*	*CDK4, CDKN2A, * ***EFNA1*** *, * ***GPX2*** *, HSP90B1,* **IL23A**, ***ODC1*** *,* **RRM2**, *SPP1*	9
**Category: Hair and Skin Development and Function^2^**				
cell spreading of epithelial cell lines		*1.01E-04*	*ANTXR1, CYR61, FN1*	3
adhesion of epithelial cell lines		*2.16E-04*	*EPHB3, FN1, POSTN, TIMP3*	4
presence of hair follicle	**6.89E-03**		**TP63**	1
development of skin		*1.18E-04*	*CLDN1, COL1A1, COL3A1, COL5A1, IRF6*	5
tensile strength of skin		*2.77E-04*	*COL5A1, DCN*	2
stratification of skin	**6.89E-03**		**TP63**	1
proliferation of epidermal cells		*8.57E-03*	*CDK4, EFNA1, IRF6, JUNB*	4
thickness of skin tissue	**1.37E-02**		**ODC1**	1
mitogenesis of skin cell lines	**2.73E-02**		**IGHG1**	1
survival of melanocytes	**2.73E-02**		**EFNA1**	1
vascularization of skin	**2.73E-02**	*5.51E-04*	*MFGE8, * ***ODC1***	2
senescence of keratinocytes		*1.36E-03*	*CDK4, CDKN2A*	2
arrest in growth of keratinocytes	**4.07E-02**		**TP63**	1
differentiation of keratinocytes		*4.47E-03*	*CDKN2A, FN1, IRF6, JUNB*	4
growth of melanocytes	**4.73E-02**	*1.89E-03*	*CDKN2A, * ***EFNA1***	2

1 IPA Diseases and Disorders. ^2^IPA Physiological System Development and Function.

3 Dermatological Diseases and Conditions: 8.43E-05–3.86E-02; Hair and Skin Development and Function: 6.89E-03–4.73E-02.

4 Dermatological Diseases and Conditions: 5.55E-10–9.26E-03; Hair and Skin Development and Function: 1.01E-04–8.57E-03.

5 type in bold denotes those genes associated with the corresponding function annotation in the CIS over BE_PC dataset; type in italics denotes those genes associated with the corresponding function annotation in the SCC over BE_PC dataset; type in both bold and italics denotes those genes associated with the corresponding function annotation in both the CIS over BE_PC and the SCC over BE_PC datasets. See Table S5 and Table S6 for corresponding tag abundance values.

6 16 unique genes identified out of 109 IPA eligible mapped IDs within the CIS over BE_PC dataset; 40 unique genes identified out of 153 IPA eligible mapped IDs within the SCC over BE_PC dataset.

In addition to those genes described in Table 4, other genes up-regulated in CIS relative to both BE and PC that are associated with epidermal development, include KRTDAP (encoded on19q13), KPRP, SPRR2F, SPRR2G, and LCE3D (all encoded on 1q21). KRTDAP is associated with epidermal morphogenesis, and is a potential regulator of keratinocyte differentiation [70], [71], [72], [73], [74]. KPRP is an epidermal marker expressed in stratified squamous epithelia [75], and has a potential role in calcium-induced keratinocyte differentiation, and expression is increased in psoriasis [76]. It is noted that genes associated with development of the cornified cell envelope encoded on 1q21/19q13, tend to be expressed at notably lower levels in invasive cancer relative to CIS (see Table S2 and Table S5).

As described in Table 4, transcriptional regulators associated with keratinocyte differentiation over-expressed within invasive cancer include IRF6, CDKN2A, and JUNB. IRF6 is an interferon-induced transcription factor associated with the switch between keratinocyte proliferation and differentiation [77]. JUNB, a member of the AP-1 transcription factor family, is associated with keratinocyte differentiation during wound healing and psoriasis [78], [79]. The cyclin-dependent protein kinase inhibitor/transcription factor CDKN2A (see above), is associated with cell cycle arrest/senescence of keratinocytes, and differentiation of epidermis [80], [81]. Although considered a tumor suppressor protein, increased expression of CDKN2A at the invasive front of basal cell carcinomas and colon cancer has been reported, and the correlation of increased invasiveness with decreased proliferation, suggests that CDKN2A may play a role in cancer cell invasion [82], [83].

An association between epidermal development and squamous cell lung cancer development is frequently studied through analysis of the EGFR pathway [84]. Anti-EGFR therapies have been initiated for various types of cancer, including NSCLC [85], [86], [87], [88]. Also, up-regulation of KGF, a member of the fibroblast growth factor family and mediator of epidermal differentiation, is associated with pancreatic cancer [89]. The identification of additional genes associated with epidermal development and up-regulated in the early stages of NSCLC, may enhance our understanding of the role of this pathway in lung cancer development, and broaden treatment options.

#### Additional IPA analysis

**Figure 6:**
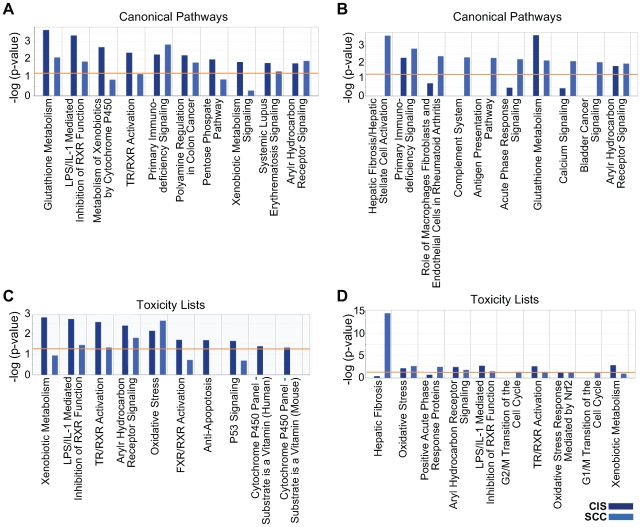
IPA canonical pathways analysis and toxicity lists analysis of the CIS over BE_PC and the SCC over BE_PC datasets. For analysis of the CIS over BE_PC dataset, 109 IPA mapped IDs were eligible; for analysis of the SCC over BE_PC dataset, 153 IPA mapped IDs were eligible. The two sets of data were displayed together using IPA core comparisons, and the 10 most significant functions within *Canonical Pathways* and *Toxicity Lists* are shown above for each dataset. The data in **A** and **C** is sorted according to highest significance in CIS over BE_PC, and the data in **B** and **D** is sorted according to highest significance in SCC over BE_PC. The orange line indicates the threshold limit of significance, preset at a p-value of 0.05.

We utilized IPA core analysis to identify additional functions within the CIS and invasive SCC datasets of up-regulated genes (**Figure 6**). *Canonical Pathways* analysis and *Toxicity Lists* analysis identified metabolism/detoxification of xenobiotics as a component of the CIS dataset, and hepatic fibrosis as a major characteristic of the invasive cancer phenotype. Specific genes associated with these phenotypes are listed in **Table 5** and **Table 6** and described below.

**Table 5:** Up-regulated genes in CIS and invasive SCC associated with metabolism/detoxification of xenobiotics according to specific categories within IPA canonical pathways and toxicity lists as indicated.

IPA Canonical & Toxicity	CIS/BE_PC^1^	SCC/BE_PC^2^	Entrez Gene Name	Location	Type(s)
**Aryl Hydrocarbon Receptor Signaling**		CDK4	cyclin-dependent kinase 4	Nucleus	kinase
		CDKN2A	cyclin-dependent kinase inhibitor 2A (melanoma, p16, inhibits CDK4)	Nucleus	transcription regulator
	FASN		fatty acid synthase	Cytoplasm	enzyme
	MCM7	MCM7	minichromosome maintenance complex component 7	Nucleus	enzyme
**Xenobiotic Metabolism Signaling**	CHST2	CHST2	carbohydrate (N-acetylglucosamine-6-O) sulfotransferase 2	Cytoplasm	enzyme
	CHST3		carbohydrate (chondroitin 6) sulfotransferase 3	Cytoplasm	enzyme
	PPP2R1B		protein phosphatase 2 (formerly 2A), regulatory subunit A, beta isoform	Unknown	phosphatase
**Metabolism of Xenobiotics by Cytochrome P450; Xenobiotic Metabolism**	AKR1C1		aldo-keto reductase family 1, member C1 (dihydrodiol dehydrogenase 1; 20-alpha (3-alpha)-hydroxysteroid dehydrogenase)	Cytoplasm	enzyme
	AKR1C3	AKR1C3	aldo-keto reductase family 1, member C3 (3-alpha hydroxy-steroid dehydrogenase, type II)	Cytoplasm	enzyme
**Cytochrome P450 Panel-substrate is a Vitamin (Human)**	CYP26A1		cytochrome P450, family 26, subfamily A, polypeptide 1	Cytoplasm	enzyme
**Glutathione Metabolism**	G6PD	G6PD	glucose-6-phosphate dehydrogenase	Cytoplasm	enzyme
**Glutathione Metabolism; NRF2-mediated Oxidative Stress Response; Oxidative Stress**	GPX2	GPX2	glutathione peroxidase 2 (gastrointestinal)	Cytoplasm	enzyme
**Glutathione Metabolism; Metabolism of Xenobiotics by Cytochrome P450; Xenobiotic Metabolism Signaling; Xenobiotic Metabolism; Aryl Hydrocarbon Receptor Signaling; NRF2-mediated Oxidative Stress Response; Oxidative Stress**	GSTM1	GSTM1	glutathione S-transferase mu 1	Cytoplasm	enzyme
	GSTM3	GSTM3	glutathione S-transferase mu 3 (brain)	Cytoplasm	enzyme
	GSTM4		glutathione S-transferase mu 4	Cytoplasm	enzyme
**NRF2-mediated Oxidative Stress Response**		ACTA1	actin, alpha 1, skeletal muscle	Cytoplasm	other
		JUNB	jun B proto-oncogene	Nucleus	transcription regulator
**Oxidative Stress**		CCL5	chemokine (C-C motif) ligand 5	Extracellular Space	cytokine

1 13 unique genes identified out of 109 IPA eligible mapped IDs. See Table S5 for corresponding tag abundance values.

2 12 unique genes identified out of 153 IPA eligible mapped IDs. See Table S6 for corresponding tag abundance values.

**Table 6:** Up-regulated genes in invasive SCC associated with tissue fibrosis according to IPA functions, canonical pathways, and toxicity lists^1^.

Gene Symbol^2^	Entrez Gene Name	Location	Type(s)	SCC/BE^5^	SCC/PC^6^	CIS/BE^5^	CIS/PC^6^
A2M	alpha-2-macroglobulin	Extracellular Space	transporter	17.4	3.2	4.1	0.8
APOE	apolipoprotein E	Extracellular Space	transporter	4.1	3	0.9	0.7
BGN	biglycan	Extracellular Space	other	>43	4.3	>5	0.5
CCL5	chemokine (C-C motif) ligand 5	Extracellular Space	cytokine	3.1	4.2	1.5	2
CDK4	cyclin-dependent kinase 4	Nucleus	kinase	3.9	3	1.7	1.3
CDKN2A	cyclin-dependent kinase inhibitor 2A (melanoma, p16, inhibits CDK4)	Nucleus	transcription regulator	11.2	3.5	5.5	1.7
COL1A1	collagen, type I, alpha 1	Extracellular Space	other	45	6.4	2	0.3
COL1A2	collagen, type I, alpha 2	Extracellular Space	other	160.5	8.2	9	0.5
COL3A1	collagen, type III, alpha 1	Extracellular Space	other	>284	40.6	>13	1.9
COL4A1	collagen, type IV, alpha 1	Extracellular Space	other	285	10.5	46	1.7
COL5A1	collagen, type V, alpha 1	Extracellular Space	other	>46	6.6	>1	0.1
COL6A3	collagen, type VI, alpha 3	Extracellular Space	other	>159	4.5	>18	0.5
DCN	decorin	Extracellular Space	other	40	4	16.7	1.7
ECE2^3^	endothelin converting enzyme 2	Plasma Membrane	peptidase	19.4	4.4	22.4	5.1
FBN1	fibrillin 1	Extracellular Space	other	18.5	3.5	7.7	1.5
FN1	fibronectin 1	Plasma Membrane	enzyme	38.6	3.9	0.7	0.1
GLIS2	GLIS family zinc finger 2	Nucleus	transcription regulator	5.4	10	1.7	3.2
IFI6	interferon, alpha-inducible protein 6	Cytoplasm	other	3.2	5.9	2.4	4.5
IFITM1	interferon induced transmembrane protein 1 (9–27)	Plasma Membrane	other	5.1	6.8	1.6	2.2
MMP12	matrix metallopeptidase 12 (macrophage elastase)	Extracellular Space	peptidase	27	4.5	9.5	1.6
MYH7B	myosin, heavy chain 7B, cardiac muscle, beta	Unknown	other	>51	4.2	>28	2.3
SPARC	secreted protein, acidic, cysteine-rich (osteonectin)	Extracellular Space	other	116	3	33	0.8
SPP1	secreted phosphoprotein 1	Extracellular Space	cytokine	17.3	30.2	1.9	3.3
TIMP3^4^	TIMP metallopeptidase inhibitor 3	Extracellular Space	other	18.5 & 5.5	3.5 & 6.9	3 & 0.9	0.6 & 1.1

1 Functions: Organismal Injury and Abnormalities (p value 2.59E-04); Canonical Pathways: Hepatic Fibrosis/Hepatic Stellate Cell Activation; Toxicity Lists: Hepatic Fibrosis.

2 24 unique genes identified out of 153 IPA eligible mapped IDs.

3 Also up-regulated in CIS.

4 Two unique tags per gene.

5 Ratio of mean TPM for 6 SCC libraries (or 5 CIS libraries) versus mean TPM for 14 BE libraries. Where mean TPM for BE libraries is 0, ratio is cited as “> mean TPM value” for SCC libraries (or CIS libraries).

6 Ratio of mean TPM for 6 SCC libraries (or 5 CIS libraries) versus average TPM for 2 PC libraries.

5,6 See Table S5 and Table S6 for corresponding tag abundance values.

#### Metabolism/detoxification of xenobiotics

Metabolism/detoxification of xenobiotics is a protective cellular response to prevent damage to macromolecules upon exposure to both exogenous and an excess of endogenous stressors [such as reactive oxygen species (ROS)]. Most notably, glutathione metabolic enzymes that mediate phase II detoxification of xenobiotics via conjugation with glutathione, and members of the aldose-reductase family of oxidoreductases, have been identified to be up-regulated in the CIS dataset (Table 5). Most of these genes are also up-regulated in the invasive cancer dataset. Functionally related genes not included within Table 5, include the oxidoreductases SRXN1 and AKR1B10. SRXN1 expression protects against cigarette smoke-induced oxidative stress, and is important for redox homeostasis [90], [91], [92]. Similar to AKR1C1, AKR1B10 catalyzes NADPH-dependent reduction and inhibition of 4-HNE and other toxic aldehydes resulting from peroxidation of membrane lipids [93]. The pentose phosphate pathway (PPP) provides reducing equivalents for glutathione reduction/recycling and maintenance of redox status; three genes associated with the PPP (ALDOC, G6PD, TKTL1), are up-regulated in CIS lesions. Expression of many of these genes (AKR1B10, AKR1C1, AKR1C3, G6PD, GPX2, GSTM1, GSTM3, GSTM4, SRXN1) is regulated by the redox-sensitive NRF2 transcription factor, and is induced by cigarette smoke [94], [95], [96], [97]. However, these protective responses may also promote adaptation to adverse environmental conditions (redox stressors), and survival with propagation of damaged cells. For example, SRXN1 may play a role in development of skin malignancies [92]. Both AKR1C isoforms and AKR1B10 catalyze oxidative activation of xenobiotic proximate carcinogen PAH trans-dihydrodiols (such as B[a]P, a component of cigarette smoke), to generate reactive ortho-quinones, and mediate redox cycling with ROS amplification [98]. Over-expression of AKR1B10 and AKR1C1 has been reported for many cancer types [99]. Expression of NRF2-regulated anti-oxidant/glutathione metabolic gene HMOX1, and NCF1 (the p47phox subunit of NADPH oxidase, a major cellular source of ROS) is up-regulated in CIS lesions, but is also relatively high in precancerous lesions (Figure 4, Table S2), suggesting that xenobiotic/oxidative stress may initiate early in the pathway leading to invasive lung cancer. It has recently been suggested that NADPH oxidase may stimulate the protective activity of the NRF2-KEAP1 signaling pathway [100].

#### Tissue fibrosis

Tissue fibrosis, initiated in response to injury and facilitated by inflammatory mediators, is characterized by the excessive accumulation of extracellular matrix components. Genes typically associated with tissue fibrosis, and up-regulated in the invasive SCC dataset described in this study, include fibrillar collagens and other fibrillar matrix components, matrix metalloproteases, metalloprotease inhibitors, proteoglycans, chemotactic proteins, transcriptional regulators, and contractile proteins (Table 6). In addition to those genes identified by IPA, other genes within the invasive cancer dataset associated with tissue fibrosis include extracellular proteins MFGE8 [101], and POSTN, a mediator of collagen fibrillogenesis [102], [103] [104]. Related genes up-regulated in the SCC dataset include constituents of the fibrillar extracellular matrix such as MFAP2 [105], [106], FBLN1 [107], and the small leucine-rich proteoglycan FMOD, a mediator of collagen fibrillogenesis and matrix assembly [108]. In addition to structural properties, many of these components, such as MFAP2, MFGE8, and POSTN have signaling activities relevant to cancer development [109], [110], [111], [112], [113].

Tissue fibrosis is a component of various cancer types, and studies suggest that advanced fibrosis contributes to aggressiveness and resistance to chemotherapy [114], [115] [116]. The myofibroblast cell, thought to originate from various sources including transformation of resident or bone marrow-derived fibroblasts, transdifferentiation of epithelial cells to mesenchymal-type cells via EMT (epithelial-mesenchymal transition), and activation of resident stellate (astrocyte) cells, is a mediator of tissue fibrosis [117]. Activation of pancreatic stellate cells mediates the fibrotic process inherent to pancreatic cancer and contributes to cancer progression [118], [119], [120], [121]. Activated hepatic stellate cells are the major mediators of liver fibrosis and contribute to liver cancer [122], [123], [124]. The epidermal growth factor receptor regulates pancreatic fibrosis via stimulation of pancreatic stellate cells [125]. The cellular origins of the tissue fibrosis apparent from analysis of the invasive lung cancer dataset presented in this study, is not known. Some genes identified in Table 6 are associated with hepatic stellate cell activation. Others, including POSTN, contribute to pancreatic stellate cell activiation [126], and also mediate EMT [127]. MFGE8 is also a mediator of EMT [111], and the cytoskeletal intermediate filament protein, VIM (over-expressed here) is a mesenchymal cell marker, and an indicator of EMT [128], [129], [130]. EMT is associated with progression to invasive cancer [131].

#### Down-regulated expression changes

We identified 84 SAGE tags to be down-regulated in CIS relative to both BE and PC (**Table S9**), and 48 SAGE tags to be down-regulated in invasive SCC relative to both BE and PC (**Table S10**), with 31 tags in common (Figure 3B). It is noted that IPA functional analysis did not identify any down-regulated genes from these datasets as specifically associated with ciliogenesis, and although multiple significant functional categories were identified, no specific biological process within these categories took prominence (data not shown). This was the case when analysed separately or as a single dataset of down-regulated genes, perhaps partially attributed to the relatively small size of the datasets. However, when based on indirect as well as direct molecular connections, IPA pathway graphical representation identified receptor tyrosine kinase ERBB2, as central to a network associated with airway biology (**Figure S4)**. In addition to associations with multiple developmental processes, ERBB2 also plays a role in airway repair including differentiation of ciliated and goblet cells, while inhibiting squamous metaplasia [132]. In this regard, it is intriguing to hypothesize that failure to initiate a potential ERBB2 signaling complex in CIS lesions, may compromise redifferentiation/restoration of the bronchial epithelium following injury, and contribute to initiation of in-situ cancer. For further description of the down-regulated genes identified in Figure S4, see **Text S2**.

We performed analysis by Gene Ontology using the GATHER annotation tool [63] for down-regulated genes in CIS and SCC relative to BE and PC (**Figure S5**; Table S8). Genes associated with defense response were most prominently identified by this analysis.

### Potential biomarkers

Genes expressed at notably different levels among normal and cancer datasets, have the potential to serve as biomarkers for early detection. For a listing of potential biomarkers for both CIS and invasive SCC, based upon a minimal 20-fold up-regulation (and a minimal average tag abundance of 40 TPM), see **Table 7**. Generally, genes associated with epidermal development (KRTDAP, SPRR2G, SPRR2E) may potentially serve as biomarkers for CIS, whereas genes associated with immune response (MHC class I receptor HLA-G, acute-phase response protein CRP) may potentially serve as biomarkers for invasive SCC. It is noted that, although precise identification of the expressing cell type(s) for the genes associated with immune response may warrant further investigation, these genes may nonetheless accurately reflect the tumor cell microenvironment and provide diagnostic potential. Multiple literature reports support the potential of HLA-G and CRP to serve as biomarkers for invasive cancer of various tissue types including NSCLC [133], [134], [135], [136], [137], [138]. Additionally, the data presented in this study suggests that the neuropeptide NTS, may have potential as a biomarker for CIS lesions, whereas CST1, a peptidase inhibitor within the cystatin superfamily, may have potential as a biomarkers for invasive SCC lesions. Intriguingly, a recent study describes the potential of CST1 as a urinary marker for colorectal cancer [139].

**Table 7:** Potential biomarkers for CIS, invasive SCC, and squamous cell lung cancer.

Tag^1^	BE Mean TPM	CIS Mean TPM	SCC Mean TPM	PC Av TPM	Gene Symbol	Mapping Reliability (%)^6^
**CIS** 2						
GGCTTCTAAC	0	903	35	31	SPRR2E	95
ACCTCCACTG	0	698	0	10	KRTDAP	91
AATCTCTCAA	16	318	13	0	NTS	95
GTCAAGCCCA	0	165	1	2	SPRR2G	91
CTGCCATTAA	2	89	4	0	OR14J1	47
TTTCCAGCAC	1	75	3	0	C6orf15	95
ACGCCTACTG	0	61	1	2	TKTL1	95
CACAGGCATC	0	58	0	0	nu_sr^5^	50
TAACCAAGAG	0	42	0	0	TTR	95
AGGAAGTCTT	1	41	1	0	nu_r^5^	48
GAAATACCCA	1	40	1	0	nu_r^5^	48
**SCC** 3						
GATCAGGCCA	0	13	284	7	COL3A1	94
CCACGGGATT	0	10	250	6	No match	–
CACCTCCTAT	1	0	239	0	nu_r^5^	48
CTGAACTGCA	0	0	133	2	HLA-G	94
GCCGTGAACA	0	0	129	6	SFTPC	94
GAGAGAGACT	0	1	127	2	CRP	95
AGGAAAGGTT	1	0	102	0	Hs.629594	49
GTACACACCC	0	0	78	0	CST1	95
CTAAGAAAGT	2	1	43	0	No match	–
**CIS and SCC** 4						
GAAATAAAGC	62	38019	14716	561	IGHG1	94
AAGGGAGCAC	31	5039	3229	32	IGL@	94
CTCCCCCAAG	33	7043	1969	32	IGHG1	94
CTCCCCCAAA	29	4772	1604	18	IGHG1	94
CAAACTAACC	10	466	516	2	IGHG1	94
GGTTGAAAAA	11	317	348	15	SNAR-E	89
AGAAGACGTT	1	730	240	0	nu_r^5^	48
GCGGAGGTGG	2	504	176	3	IGHG1	89
AAATAAAGCA	1	392	174	0	nu_r^5^	48
CTGGGTGCCT	0	527	172	3	PSMB4	54
GGAAATAAAG	3	309	134	2	nu_r^5^	48
GAAGCCCCAG	0	300	101	0	IGKC	94
AGGGTCCCCG	1	266	97	0	IGKC	94
TGCCGTTTTG	2	192	94	2	GSTM3	92
GAGATAAAGC	0	250	78	3	Hs.682707	48
GAAATAAGGC	2	246	77	0	SLCO1A2	51
TTGAAACTGT	0	67	61	0	MID1	67
GAAATAGAGC	0	190	57	0	nu_r^5^	48
TTAAATTAAT	2	51	48	0	NTRK2	94
AGGGGAGCAC	0	45	45	0	LOC100287927	67

1 Criteria for potential biomarkers was set at a minimal of 20-fold enhanced expression based on normalized mean tag counts, and a minimal mean abundance level of 40 TPM, in the marker dataset.

2 Tags detected at a minimal of 20-fold enhanced expression in CIS relative to BE, PC, and SCC SAGE datasets.

3 Tags detected at a minimal of 20-fold enhanced expression in SCC relative to BE, PC, and CIS SAGE datasets.

4 Tags detected at a minimal of 20-fold enhanced expression in both CIS and SCC relative to both BE and PC SAGE datasets.

5 Tags map to cDNA sequences from the database of Unclustered ESTs.

6 Mapping reliability as defined by SAGE Genie.

Due to rarity of CIS specimens, we were not able to validate up-regulation of gene expression directly. However, an analysis of publically available microarray expression data from squamous lung carcinoma specimens [35], and microarray expression data from internally profiled bronchial brushings, indicates that genes associated with epidermal development and showing specific expression in the CIS SAGE dataset, such as KRTDAP and SPRR2G, may actually retain up-regulation in a subset of invasive tumors (**Figure S6**). Thus, these genes may have potential to serve as early-stage biomarkers for SCC in a combinatorial manner.

To identify potential biomarkers associated with both preinvasive and invasive squamous cell lung cancer, we selected SAGE tags on the basis of a minimal 20-fold up-regulation in both the CIS and invasive cancer datasets (relative to BE and PC) (Table 7). Intriguingly, several of these tags map to immunoglobulin heavy chain and light chain genes (see above). Other genes identified here include NTRK2, a member of the neurotrophin tyrosine kinase receptor family, and GSTM3, a mediator of glutathione/xenobiotic metabolism (see above). Genetic polymorphisms of GSTM3 and other members of the GST family of proteins, have been associated with cancer risk [140], [141], [142]. Enhanced expression of NTRK2 has been associated with poor prognosis in neuroblastoma and other cancer types [143], [144], [145]. It is noted that several of the selected tags in Table 7 are currently unmapped to a gene ID; further experimentation to resolve these mappings may provide additional genes for biomarker evaluation.

In an effort to evaluate the frequency of over-expression of these candidate genes in SCC on a broader scale, we again consulted publically available microarray expression data from squamous lung carcinoma specimens [35], and microarray expression data from internally profiled bronchial brushings. Although sporadic, a trend of up-regulation was observed for COL3A1, SFTPC, CST1, IGHG1, GSTM3, SLCO1A2, and NTRK2 in squamous tumors relative to bronchial epithelium, lending support to the potential of these genes to serve as biomarkers for invasive SCC (Figure S6). See **Table S11** for raw microarray data used for this analysis.

### Differential gene expression and genomic copy-number status in CIS lesions

**Figure 7:**
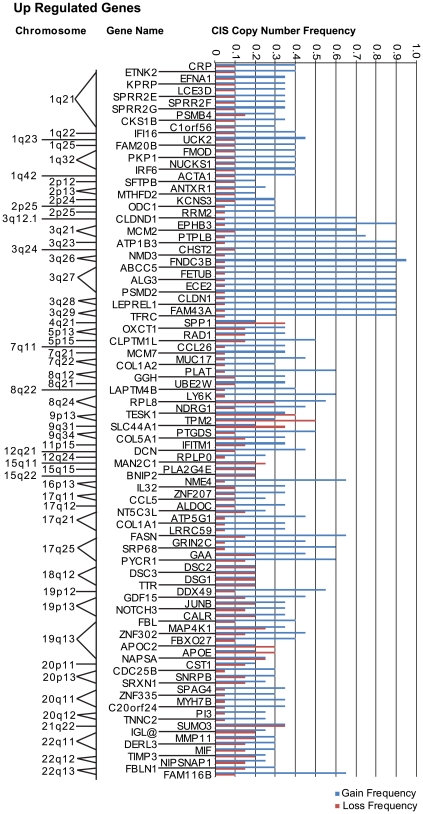
Correlation between up-regulated gene expression in CIS and SCC relative to BE and PC, with regions of frequent copy-number gain in CIS specimens. Up-regulated genes (x-axis), plotted according to chromosomal location as indicated, were matched with segmental copy-number status (y-axis), defined by frequent copy-number gain (blue) and loss (red), from 20 independent CIS specimens. 224 genes were analyzed, and only those associated with regions gained at a minimal frequency of 0.2 are shown above. Knowledge of losses in addition to gains serves as a filter to identify those chromosomal regions that are preferentially gained rather than a reflection of general instability. See Table S12 for raw data pertaining to these analyses.

**Figure 8:**
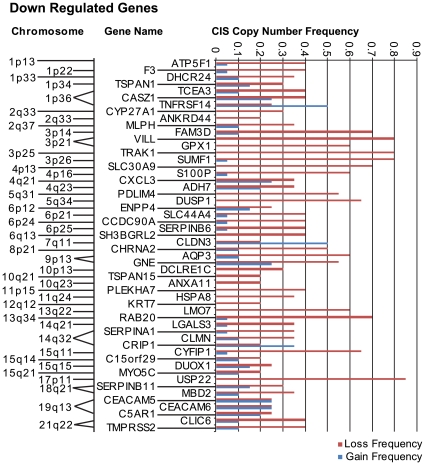
Correlation between down-regulated gene expression in CIS and SCC relative to BE and PC, with regions of frequent copy-number loss in CIS specimens. Down-regulated genes (x-axis), plotted according to chromosomal location as indicated, were matched with segmental copy-number status (y-axis), defined by frequent copy-number loss (red) and gain (blue), from 20 independent CIS specimens. 81 genes were analyzed, and only those associated with regions lost at a minimal frequency of 0.2 are shown above. Knowledge of gains in addition to losses serves as a filter to identify those chromosomal regions that are preferentially lost rather than a reflection of general instability. See Table S13 for raw data pertaining to these analyses.

To investigate whether alterations in gene dosage contributes mechanistically to the differential gene expression identified here in early-stage lung cancer, we compared segmental copy number gain/loss from 20 independent CIS specimens with locus information for up-regulated and down-regulated genes in CIS/SCC relative to BE and PC. A subset of up-regulated genes localized to regions of stable frequent gain (**Figure 7**), and a subset of down-regulated genes localized to regions of stable frequent loss (**Figure 8**). Most prominently identified corresponding regions of copy number gain include 1q21–1q42.13 and 3q12.1–3q29, and loci within chromosomal arms 7q, 8q, 17q, and 20q. It is noted that the region 1q21 encodes the EDC, a region over-expressed early in CIS lesions (see above). Most prominently identified corresponding regions of copy number loss include loci within chromosomal arms 3p and 6p. This data agrees with previously published data for lung cancer, particularly amplification at 3q, 7q, 8q, and loss of 3p [146], [147], [148]. [Notably, a recent study describes genomic amplification and over-expression of transcription factor SOX2 encoded at 3q26.33 in lung squamous cell carcinoma [149]. Although we detect enhanced expression of SOX2 in CIS relative to both BE and PC, the tag abundance ratio falls marginally below the three-fold threshold/cut-off applied in this study, precluding this gene from the copy-number analysis presented here.] The data presented here suggests that frequent chromosomal gain/loss of specific loci, represents a significant mechanism for differential gene expression in early, preinvasive stages of squamous cell lung cancer. See **Table S12** and **Table S13** for raw data describing copy-number status for up-regulated genes and down-regulated genes, respectively.

### Summary

In this study we describe genes differentially regulated in early stages of squamous NSCLC development by way of SAGE profiling. Differentially expressed genes found in common between CIS and precancerous lesions relative to bronchial epithelium, are presumed to reflect early expression changes during CIS development. Those genes differentially expressed in CIS relative to the precancerous lesions/bronchial epithelium, and in invasive SCC relative to the precancerous lesions/bronchial epithelium, presumably reflect gene expression changes more instrumental to cancer initiation, and cancer cell invasion, respectively. In this study, data was analyzed primarily through the use of *Ingenuity Pathway Analysis*, complemented by literature searches pertaining to specific genes. Here we have described the up-regulation of genes associated with epidermal development, and the down-regulation of genes associated with mucociliary development, in both CIS and precancerous lesions relative to bronchial epithelium. Increased expression of genes associated with desmosomal cell-cell junctions, and epidermal barrier formation, would conceivably enhance tissue integrity, and may reflect a protective response to tissue damage occurring early in CIS lesions. Although genes associated with epidermal development are also elevated in SCC, those genes specifically associated with epidermal barrier formation and desmosomal structures, show relatively low expression in invasive SCC, suggesting further tissue architectural changes upon transition to invasive cancer. Our data also suggests that tissue remodeling/fibrosis is present in early stage CIS lesions, where it may reflect a cellular response to hypoxia/oxidative stress. Our analysis has identified up-regulation of genes associated with xenobiotic metabolism/detoxification in CIS and invasive SCC relative to bronchial epithelium and precancerous lesions, implying an enhanced requirement for protection against electrophile and/or oxidative stress upon the transition from precancer to CIS. Up-regulated genes specifying tissue fibrosis is a pronounced feature of the invasive cancer dataset, where it appears in association with acute phase immune components. Thus, the data presented here suggests that a fibrotic tissue response is initiated in early stage CIS, and is further developed in invasive cancer. Considering that many of these matrix components have signaling activities associated with regulation of cellular proliferation and migration similar to those described for EMT, the profibrotic phenotype described here may represent a defining component of advanced lung SCC. Additionally, by selecting SAGE tags showing extreme up-regulation among the various datasets, we have identified a small number of genes that may have potential as biomarkers for early diagnosis. Although some of these genes have previously been investigated as biomarkers for invasive cancer by other researchers, this is the first description of potential biomarkers for CIS. Lastly, a comparative analysis between differential gene expression in CIS lesions and invasive carcinoma with array CGH data from independent CIS specimens, suggests that copy number alterations plays a significant role in differential gene expression in CIS lesions.

## Supporting Information

Table S1 Most abundant 300 tags in bronchial epithelium, carcinoma-in-situ, and invasive cancer SAGE datasets.
(0.39 MB DOC)

Table S2 Up-regulated gene expression changes in common between carcinoma-in-situ and precancerous lesions relative to bronchial epithelium.
(0.29 MB DOC)

Table S3 Input for GATHER analysis of gene ontology for genes showing similar differential expression in CIS and PC.
(0.39 MB DOC)

Table S4 Down-regulated gene expression changes in common between carcinoma-in-situ and precancerous lesions relative to bronchial epithelium.
(0.97 MB DOC)

Table S5 Up-regulated gene expression changes in carcinoma-in-situ relative to bronchial epithelium and precancerous lesions.
(0.34 MB DOC)

Table S6 Up-regulated gene expression changes in invasive cancer relative to bronchial epithelium and precancerous lesions.
(0.35 MB DOC)

Table S7 Summary of real-time RT-PCR data for select genes within invasive cancer, bronchial epithelium, and lung parenchyma.
(0.03 MB DOC)

Table S8 Input for GATHER analysis of gene ontology for genes differentially expressed in cancer datasets relative to BE and PC.
(0.16 MB DOC)

Table S9 Down-regulated gene expression changes in carcinoma-in-situ relative to bronchial epithelium and precancerous lesions.
(0.14 MB DOC)

Table S10 Down-regulated gene expression changes in invasive cancer relative to bronchial epithelium and precancerous lesions.
(0.09 MB DOC)

Table S11 Microarray expression data for up-regulated genes with biomarker potential.
(0.19 MB DOC)

Table S12 Up-regulated genes in CIS and SCC analyzed for frequent copy-number gain (and loss) in CIS specimens.
(0.33 MB DOC)

Table S13 Down-regulated genes in CIS and SCC analyzed for frequent copy-number loss (and gain) in CIS specimens.
(0.14 MB DOC)

Figure S1 Gene ontology analysis as determined by the GATHER annotation tool for genes showing similar differential expression in CIS and PC. Those gene ontology annotations cited at a minimal depth of five and associated with a positive ln(Bayes factor) value are shown. The y-axis refers to the total number of genes per GO annotation. A. Up-regulated genes in CIS and PC relative to BE (151 genes analyzed). ln(Bayes factor) range 25.15–0.06 (left to right as indicated). B. Down-regulated genes in CIS and PC relative to BE (484 genes analyzed). ln(Bayes factor) range 8.04–0.12 (left to right as indicated). A positive Bayes factor indicates support for the hypothesis that an association of an annotation with the identified gene cluster is stronger than the association to other genes in the genome. See Table S3 for exact listing of gene symbols used as input for GATHER analysis.
(1.96 MB EPS)

Figure S2 IPA pathway graphical representation for genes commonly down-regulated in CIS and PC relative to BE. Results are displayed for 76 gene products characterized by either direct (solid lines) or indirect (broken lines) connections derived from analysis of 500 input molecules*. Lines indicate protein-protein binding interactions, and arrows refer to “acts on” interactions such as proteolysis, expression, and protein-DNA/RNA interactions. Gene products are positioned according to subcellular localization. Genes associated with cilia formation (IPA function: Cellular Assembly and Organization) are highlighted. (See Table S4 for tag data.) *To comply with the maximum input of 500 mapped IDs for IPA pathway analysis, 8 IDs from the BE over CIS_PC dataset were excluded from the total of 508 IPA mapped IDs. These included C10orf79, C13orf30, C16orf46, C1orf110, C1orf92, C22orf15, C5orf32, and C8orf40, chosen randomly from within the set of open reading frame genes.
(6.95 MB EPS)

Figure S3 Gene ontology analysis as determined by the GATHER annotation tool for genes up-regulated in cancer datasets relative to BE and PC. Those gene ontology annotations cited at a minimal depth of five and associated with a positive ln(Bayes factor) value are shown. The y-axis refers to the total number of genes per GO annotation. A. Up-regulated genes in CIS relative to BE and PC (138 genes analyzed). ln(Bayes factor) range 3.4–0.19 (left to right as indicated). B. Up-regulated genes in SCC relative to BE and PC (173 genes analyzed). ln(Bayes factor) range 9.66–0.13 (left to right as indicated). A positive Bayes factor indicates support for the hypothesis that an association of an annotation with the identified gene cluster is stronger than the association to other genes in the genome. See Table S8 for exact listing of gene symbols used as input for GATHER analysis.
(3.31 MB EPS)

Figure S4 Genes down-regulated in the CIS and invasive SCC datasets relative to BE and PC. A. Venn diagram of down-regulated SAGE tags and corresponding IPA mapped IDs for the CIS and SCC datasets. (See Table S9 and Table S10 for tag data.) B. IPA pathway graphical representation for the BE_PC over CIS dataset (50 unique IDs displayed in green; 26 shared IDs displayed in gray), and the BE_PC over SCC dataset (15 unique IDs displayed in red; 26 shared IDs displayed in gray). Gene products are positioned according to subcellular localization. Both direct connections (solid lines) and indirect connections (broken lines) among the individual gene products are shown; lines indicate protein-protein binding interactions, and arrows refer to “acts on” interactions such as proteolysis, expression, and protein-DNA/RNA interactions.
(6.31 MB EPS)

Figure S5 Gene ontology analysis as determined by the GATHER annotation tool for genes down-regulated in cancer datasets relative to BE and PC. Those gene ontology annotations cited at a minimal depth of five and associated with a positive ln(Bayes factor) value are shown. The y-axis refers to the total number of genes per GO annotation. A. Down-regulated genes in CIS relative to BE and PC (74 genes analyzed). ln(Bayes factor) range 2.23–0.06 (left to right as indicated). B. Down-regulated genes in SCC relative to BE and PC (39 genes analyzed). ln(Bayes factor) range 3.5–0.07 (left to right as indicated). A positive Bayes factor indicates support for the hypothesis that an association of an annotation with the identified gene cluster is stronger than the association to other genes in the genome. See Table S8 for exact listing of gene symbols used as input for GATHER analysis.
(2.74 MB EPS)

Figure S6 Validation of up-regulated gene expression in invasive SCC using microarray data. Microarray expression data for 53 squamous lung tumors (SqCC) was downloaded from NCBI Lung Cancer Dataset, GEO accession number GSE3141 [35], and microarray expression data for 67 bronchial brushings (Normal) was internally profiled. Results are shown above for seven genes up-regulated in the invasive SCC SAGE dataset relative to BE and PC (COL3A1, CST1, *GSTM3, *IGHG1, *NTRK2, SFTPC, and *SLCO1A2), and for two genes up-regulated in the CIS SAGE dataset relative to BE, PC, and invasive SCC (KRTDAP, SPRR2G). See Table S11 for raw microarray data. *Also up-regulated in CIS.
(1.75 MB EPS)

Text S1 Further description of the down-regulated genes displayed in Figure S2.
(0.14 MB DOC)

Text S2 Further description of the down-regulated genes displayed in Figure S4.
(0.08 MB DOC)
