# Supplementary material for: Transcriptome Profiles of Carcinoma-in-Situ and Invasive Non-Small Cell Lung Cancer as Revealed by SAGE
Source: PLoS One. 2010 Feb 11;5(2):e9162. doi: 10.1371/journal.pone.0009162 (PMC2820080; doi:10.1371/journal.pone.0009162)
Supplement: Table S1 — Most abundant 300 tags in bronchial epithelium, carcinoma-in-situ, and invasive cancer SAGE datasets. (0.39 MB DOC) [file pone.0009162.s001.doc]

**Table S1.** **Most abundant 300 tags in bronchial epithelium, carcinoma-in-situ, and invasive cancer SAGE datasets.**

| **BE Tag^1^** | **BE TPM^2^** | **BE Gene Symbol^3^** | **CIS Tag^4^** | **CIS TPM^5^** | **CIS Gene Symbol^3^** | **SCC Tag^6^** | **SCC TPM^7^** | **SCC Gene Symbol^3^** |
| --- | --- | --- | --- | --- | --- | --- | --- | --- |
| CTTTGAGTCC | 42246 | SCGB1A1 | GAAATAAAGC | 38019 | IGHG1 | GAAATAAAGC | 14716 | IGHG1 |
| ACTTTTTCAA | 13039 |  | ACTTTTTCAA | 10467 |  | ACTTTTTCAA | 7612 |  |
| CCTATCAGTA | 7191 | MSMB | CTTCCTTGCC | 10219 | KRT17 | ATAATTCTTT | 6480 | RPS29 |
| TTCATACACC | 6752 |  | AAAAAAAAAA | 8714 |  | TTCAATAAAA | 6089 | FAM102B |
| CACCTAATTG | 6487 | LOC100133315 | CTCCCCCAAG | 7043 | IGHG1 | GTTGTGGTTA | 5811 | B2M |
| GTTGTGGTTA | 5851 | B2M | GATCTCTTGG | 6836 | S100A2 | TACCATCAAT | 5759 | GAPDH |
| CCCATCGTCC | 5581 |  | ATAATTCTTT | 6202 | RPS29 | AAAAAAAAAA | 5670 |  |
| AAAAAAAAAA | 5032 |  | TTCAATAAAA | 5225 | FAM102B | TAGGTTGTCT | 5658 | TPT1 |
| AGCCCTACAA | 4419 |  | TAGGTTGTCT | 5120 | TPT1 | CTGGGTTAAT | 5516 | RPS19 |
| TAGGTTGTCT | 4083 | TPT1 | AAGGGAGCAC | 5039 | IGL@ | CCCATCGTCC | 5482 |  |
| AAGCTCGCCG | 3651 | SCGB3A1 | AAACCCCAAT | 4949 | IGL@ | CTTCCTTGCC | 5293 | KRT17 |
| TTCAATAAAA | 3509 | FAM102B | TACCTGCAGA | 4937 | S100A8 | TTGGGGTTTC | 5092 | FTH1 |
| GTGAAACCCC | 3311 | LEP | CTCCCCCAAA | 4772 | IGHG1 | TTCATACACC | 4910 |  |
| GTTCACATTA | 3311 | CD74 | CCACTGCACT | 4537 | ALPP | CAATAAATGT | 4181 | RPL37 |
| ATAATTCTTT | 3255 | RPS29 | CCCATCGTCC | 4525 |  | GTGAAACCCC | 3738 | LEP |
| TTGGGGTTTC | 3252 | FTH1 | TTCATACACC | 4451 |  | GAGGGAGTTT | 3707 | RPL27A |
| CTAAGACTTC | 3123 |  | GAGGGAGTTT | 4190 | RPL27A | CACCTAATTG | 3561 | LOC100133315 |
| CCACTGCACT | 3118 | ALPP | GTTGTGGTTA | 4183 | B2M | TAATAAAGGT | 3295 | RPS8 |
| TGTGGGAAAT | 3102 | SLPI | CTGGGTTAAT | 4074 | RPS19 | AAGGGAGCAC | 3229 | IGL@ |
| TTGGTCCTCT | 2930 | RPL41 | GTGAAACCCC | 3990 | LEP | GGATTTGGCC | 3205 | RPLP2 |
| TGATTTCACT | 2845 | hCG_1791940 | TTTCCTGCTC | 3458 | SPRR3 | GTTCACATTA | 3069 | CD74 |
| ACTAACACCC | 2678 |  | GGATTTGGCC | 3367 | RPLP2 | TTGGTCCTCT | 2999 | RPL41 |
| TGTGTTGAGA | 2619 | EEF1A1 | TAATAAAGGT | 3077 | RPS8 | CCACTGCACT | 2989 | ALPP |
| CCAAGGTGGC | 2567 | C20orf114 | TCTCCATACC | 3052 |  | GAAAAATGGT | 2921 | RPSA |
| CTGTACAGAC | 2497 | TUBB2C | TTGGGGTTTC | 3008 | FTH1 | GCATAATAGG | 2645 | RPL21 |
| TAATAAAGGT | 2475 | RPS8 | TACCATCAAT | 2940 | GAPDH | CCTGTAATCC | 2582 | RASA4 |
| CTCCACCCGA | 2413 | TFF3 | AAAGCACAAG | 2924 | KRT6A | CACAAACGGT | 2571 | RPS27 |
| TCTCCATACC | 2363 |  | GCATAATAGG | 2907 | RPL21 | GATCTCTTGG | 2471 | S100A2 |
| CCTGTAATCC | 2351 | RASA4 | TTGGTCCTCT | 2773 | RPL41 | TCAGATCTTT | 2416 | RPS4X |
| GAGGGAGTTT | 2251 | RPL27A | AGAAAGATGT | 2671 | ANXA1 | AGGCTACGGA | 2385 | RPL13A |
| AAAACATTCT | 2174 | LOC100132618 | CAATAAATGT | 2586 | RPL37 | AGGAAAGCTG | 2359 | RPL36 |
| TCAGATCTTT | 2127 | RPS4X | CCTGTAATCC | 2542 | RASA4 | GAAATACAGT | 2290 | NT5C |
| GCATAATAGG | 2083 | RPL21 | TAAAAAAAAA | 2246 | ELF5 | CTAAGACTTC | 2279 |  |
| GTGATCAGCT | 2036 | MUC5AC | GTGAAACCCT | 2225 | LILRB1 | ATGTGAAGAG | 2226 | SPARC |
| GTGAAACCCT | 1865 | LILRB1 | GAAAAATGGT | 2224 | RPSA | AGCCCTACAA | 2208 |  |
| GAAATACAGT | 1854 | NT5C | GAAAAAAAAA | 2185 | ITGB2 | TGGAAATGAC | 2140 | COL1A1 |
| CTGACCAGAG | 1850 | CAPS | GTGGCCACGG | 2184 | S100A9 | GTGGCCACGG | 2103 | S100A9 |
| GCTAACCCCT | 1810 | TPPP3 | CACCTAATTG | 2078 | LOC100133315 | AAGACAGTGG | 2094 | RPL37A |
| CCTCAGGATA | 1783 | TGFA | TGCACGTTTT | 1998 | RPL32 | GTGACCACGG | 2087 | GRIN2C |
| ATTTTCTAAA | 1775 | AGR2 | GTTCACATTA | 1958 | CD74 | CCTAGCTGGA | 2051 | PPIA |
| TTCACTGTGA | 1745 | LGALS3 | TCAGATCTTT | 1954 | RPS4X | TACCTGCAGA | 2039 | S100A8 |
| CTGGGTTAAT | 1673 | RPS19 | TAAGGAGCTG | 1920 | RPS26 | TTGGTGAAGG | 2006 | TMSB4X |
| GAAAAATGGT | 1608 | RPSA | AACTAAAAAA | 1865 | EPRS | GTGAAACCCT | 1982 | LILRB1 |
| AATGCTTTGT | 1564 | TUBA1A | TACCCTAAAA | 1830 |  | CTCCCCCAAG | 1969 | IGHG1 |
| ATTTGAGAAG | 1562 | RAD23B | AGGGCTTCCA | 1780 | RPL10 | CCCTGGGTTC | 1934 | FTL |
| GGATTTGGCC | 1560 | RPLP2 | ATTCTCCAGT | 1745 | RPL23 | TGCACGTTTT | 1903 | RPL32 |
| CAATTAAAAG | 1544 | XBP1 | AGGAAAGCTG | 1700 | RPL36 | CCAGAACAGA | 1887 | RPL30 |
| TAAAAAAAAA | 1499 | ELF5 | ATCCTTGCTG | 1693 | CSTA | AGGGCTTCCA | 1883 | RPL10 |
| TGCACGTTTT | 1491 | RPL32 | AGAAAAAAAA | 1642 | CRADD | TGTGTTGAGA | 1877 | EEF1A1 |
| CTCATAAGGA | 1490 |  | AGCCCTACAA | 1642 |  | AAAGCACAAG | 1822 | KRT6A |
| AGAAAGATGT | 1469 | ANXA1 | GCAGGGCCTC | 1600 | FXYD3 | TAAGGAGCTG | 1744 | RPS26 |
| GCAGGGCCTC | 1460 | FXYD3 | CCAGAACAGA | 1562 | RPL30 | AAGGTGGAGG | 1730 | RPL18A |
| TGCCCTCAGG | 1453 | LCN2 | CACAAACGGT | 1557 | RPS27 | TAAAAAAAAA | 1726 | ELF5 |
| CAAGCATCCC | 1438 |  | CCTAGCTGGA | 1531 | PPIA | GGGCTGGGGT | 1723 | RPL29 |
| CACAAACGGT | 1437 | RPS27 | CAAAAAAAAA | 1495 | ADA | AAACCCCAAT | 1692 | IGL@ |
| GCAGCGGCAG | 1421 | CTSW | GGGCTGGGGT | 1458 | RPL29 | ATTCTCCAGT | 1639 | RPL23 |
| TGCCTCACCT | 1398 | PLUNC | AGCACCTCCA | 1392 | EEF2 | AGCACCTCCA | 1639 | EEF2 |
| CAATAAATGT | 1370 | RPL37 | AAGACAGTGG | 1389 | RPL37A | GCCCCTGCTG | 1618 | KRT5 |
| TACATAATTA | 1317 | NCRNA00084 | AACTAACAAA | 1375 | RPS27A | GCAGCCATCC | 1615 | RPL28 |
| GGGCTGGGGT | 1314 | RPL29 | AGGCTACGGA | 1345 | RPL13A | CTCCCCCAAA | 1604 | IGHG1 |
| AGCTTAATGA | 1283 | LRRC16A | TTTCCTCTCA | 1312 | SFN | GTCTGGGGCT | 1541 | TAGLN2 |
| GGCCCAGGCC | 1268 | ALDH3A1 | GAAATACAGT | 1287 | NT5C | AGAAAAAAAA | 1532 | CRADD |
| GAAAAAAAAA | 1244 | ITGB2 | GAAGCAGGAC | 1223 | EFEMP2 | TGAGGGAATA | 1531 | TPI1 |
| GTAAAAAAAA | 1237 | ATF3 | CCATTGCACT | 1214 | SLC2A6 | AATAGGTCCA | 1464 | RPS25 |
| AAGACAGTGG | 1217 | RPL37A | AAAACATTCT | 1214 | LOC100132618 | GAAAAAAAAA | 1456 | ITGB2 |
| ATTCTCCAGT | 1213 | RPL23 | CTCATAAGGA | 1208 |  | TGATTTCACT | 1454 | hCG_1791940 |
| TACCATCAAT | 1177 | GAPDH | GCAGCCATCC | 1202 | RPL28 | GAAGCAGGAC | 1453 | EFEMP2 |
| AGGCTACGGA | 1150 | RPL13A | ACACAGCAAG | 1198 | EMID2 | GGCAAGCCCC | 1449 | RPL10A |
| GGAACAAACA | 1148 | CD24 | ACTCCAAAAA | 1184 | RPS15 | ATGGCTGGTA | 1408 | RPS2 |
| CAACTAATTC | 1134 | CLU | ACATCATCGA | 1178 | RPL12 | TGTACCTGTA | 1391 | TUBA1B |
| AAATAAAAGC | 1133 | EZR | GGCAAGCCCC | 1160 | RPL10A | GCAGGGCCTC | 1384 | FXYD3 |
| GAGGCCAAGA | 1122 | GSTA1 | CTAAGACTTC | 1155 |  | AACTAAAAAA | 1383 | EPRS |
| GCCTTCCAAT | 1120 | DDX5 | GCAAAAAAAA | 1144 | RCE1 | AAAACATTCT | 1343 | LOC100132618 |
| AATAGGTCCA | 1120 | RPS25 | GGTGGTGTCT | 1138 | GPX2 | TACCCTAAAA | 1339 |  |
| TACCTCTGAT | 1120 | S100P | AACCCGGGAG | 1118 | ZNF669 | GTGCACTGAG | 1310 | HLA-C |
| AGGTCCTAGC | 1087 | GSTP1 | GCCCCTGCTG | 1083 | KRT5 | CGCCGCCGGC | 1278 | RPL35 |
| AGCACCTCCA | 1084 | EEF2 | AATAGGTCCA | 1075 | RPS25 | TTTGGTTTTC | 1269 | COL1A2 |
| AAAGTTATTT | 1071 | FOXJ1 | GTAAGTGTAC | 1069 |  | ACATCATCGA | 1212 | RPL12 |
| GCGAAACCCT | 1059 | DKFZp761E198 | GTGTGGGGGG | 1061 | JUP | CTCATAAGGA | 1193 |  |
| GCTTTGATGA | 1056 | EPHX1 | TGCCCTCAGG | 1048 | LCN2 | TCTCCATACC | 1177 |  |
| ACCCTTGGCC | 1047 | HNRNPM | TGAGGGAATA | 1047 | TPI1 | TGGGCAAAGC | 1177 | EEF1G |
| TAAGGAGCTG | 1030 | RPS26 | CAATTAAAAG | 1044 | XBP1 | GGCCCAGGCC | 1123 | ALDH3A1 |
| GGAACTGTGA | 1023 | TSPAN1 | ATTATTTTTC | 1037 | RPL7 | AGAAAGATGT | 1116 | ANXA1 |
| GGCAAGCCCC | 999 | RPL10A | GACAAAAAAA | 1036 | TWIST2 | CATATCATTA | 1055 | IGFBP7 |
| AGTCAGGATA | 994 | CCDC78 | GCATTTAAAT | 1007 | EEF1B2 | TGGTGTTGAG | 1050 | RPS18 |
| TTTGGGCCTA | 986 | CRIP1 | AGCTTCTACC | 1006 | HCG9 | ACCAAAAACC | 1044 | COL1A1 |
| GTGAAGGCAG | 983 | RPS3A | GTAAAAAAAA | 994 | ATF3 | GGACCACTGA | 1043 | RPL3 |
| TTTGAAATGA | 966 | SAT1 | TTGGTGAAGG | 989 | TMSB4X | GAGCAGCGCC | 1041 | S100A7 |
| CAAAAAAAAA | 965 | ADA | GGAAAAAAAA | 976 | HGF | AACTAACAAA | 1015 | RPS27A |
| TGAGCTTGTG | 940 | MS4A8B | TTGGCCAGGC | 971 | NDUFV3 | CCCGTCCGGA | 1010 | RPL13 |
| CCTAGCTGGA | 939 | PPIA | GCCTTCCAAT | 968 | DDX5 | CTCCCAGCCA | 1009 | SFTPA2B |
| GGAATGCCTC | 934 | C2orf40 | GTGAAGGCAG | 963 | RPS3A | TTGGCCAGGC | 992 | NDUFV3 |
| CCAGAACAGA | 933 | RPL30 | CAATAAACTG | 960 | EIF1 | CCCCAGCCAG | 982 | RPS3 |
| AGGGCTTCCA | 931 | RPL10 | TTGAATCCCC | 952 | PI3 | ACATTCTTTT | 970 | GPNMB |
| AGGAAAGCTG | 928 | RPL36 | AACCCGGGAA | 948 |  | GACGACACGA | 965 | RPS28 |
| ATTGTTTATG | 916 | TRMT5 | AAAAATAAAG | 948 | ATP5A1 | CAAGCATCCC | 959 |  |
| TACCCTAAAA | 907 |  | GGACCACTGA | 942 | RPL3 | GCAAAAAAAA | 956 | RCE1 |
| CACTACTCAC | 903 |  | TTGTAATCGT | 934 | OAZ1 | GCTTTATTTG | 954 | ACTB |
| GGACCACTGA | 892 | RPL3 | CGCCGCCGGC | 918 | RPL35 | TTGTAATCGT | 948 | OAZ1 |
| GCAGCCATCC | 873 | RPL28 | GTCTGGGGCT | 913 | TAGLN2 | GCCCCCAATA | 930 | LGALS1 |
| AAGGAGATGG | 860 | RPL31 | TGTACCTGTA | 910 | TUBA1B | GTAAGTGTAC | 928 |  |
| CATATCATTA | 859 | IGFBP7 | GGCTTCTAAC | 903 | SPRR2E | ACTCCAAAAA | 927 | RPS15 |
| ACATCATCGA | 841 | RPL12 | GTACGTATTC | 877 | IGJ | GCATTTAAAT | 923 | EEF1B2 |
| GTGGTACAGG | 839 | PRDX5 | TGGGCAAAGC | 840 | EEF1G | TAATAAAGAA | 916 | KRT15 |
| AAAAATAAAG | 829 | ATP5A1 | TGTGTTGAGA | 839 | EEF1A1 | GTTCGTGCCA | 913 | RPL35A |
| GATAGTTGTG | 828 | HMGN3 | GTGCACTGAG | 832 | HLA-C | GGCTGGGGGC | 899 | PFN1 |
| CCATTGCACT | 814 | SLC2A6 | GACGACACGA | 828 | RPS28 | TGCCCTCAGG | 890 | LCN2 |
| GTGCACTGAG | 807 | HLA-C | GTCCCTGCCT | 811 | GSTM1 | CAAAAAAAAA | 889 | ADA |
| GCCTGCTGGG | 798 | GPX4 | AGGTCAGGAG | 809 | KIAA0101 | GTGAAGGCAG | 887 | RPS3A |
| TTTTTAATGT | 793 | H3F3A | CAAGCATCCC | 806 |  | CAGGCCCCAC | 883 | S100A11 |
| TTGGAGATCT | 785 | NDUFA4 | AAGGTGGAGG | 771 | RPL18A | AAGGAGATGG | 881 | RPL31 |
| GGAAAAAAAA | 782 | HGF | TGATTTCACT | 758 | hCG_1791940 | AAGTTGCTAT | 878 | PSAP |
| GGGCATCTCT | 781 | HLA-DRA | CCACAGGAGA | 754 | PERP | ACTAACACCC | 873 |  |
| GTGCTGAATG | 771 | MYL6 | ACTTACCTGC | 747 | COX6B1 | GTAAAAAAAA | 870 | ATF3 |
| TTGGCCAGGC | 770 | NDUFV3 | CACTTGCCCT | 744 | ACSS2 | AAAAATAAAG | 869 | ATP5A1 |
| TGCCCTCAAA | 766 | LCN2 | ATGGCTGGTA | 744 | RPS2 | GGGCATCTCT | 867 | HLA-DRA |
| TGTGCTAAAT | 765 | RPL34 | GGAATGTACG | 743 | ATP5G3 | AATCCTGTGG | 863 | RPL8 |
| TTGGTGAAGG | 764 | TMSB4X | GGGCATCTCT | 738 | HLA-DRA | ATTTGAGAAG | 861 | RAD23B |
| TGGGCAAAGC | 745 | EEF1G | AGAAGACGTT | 730 |  | GTGCTGAATG | 847 | MYL6 |
| ACACAGCAAG | 744 | EMID2 | GAATTTTATA | 722 | TSPO | TGTGCTAAAT | 841 | RPL34 |
| TCGAAGCCCC | 739 |  | ACATTCTTTT | 719 | GPNMB | GGGAAGCAGA | 839 | F11R |
| AAGGTGGAGG | 735 | RPL18A | TTTTCTGAAA | 716 | TXN | GCCGAGGAAG | 836 | RPS12 |
| CAGTTCTCTG | 734 | TMEM66 | AACCCAGGAG | 710 | GK5 | TGTGATCAGA | 826 | ATP5L |
| GACGACACGA | 731 | RPS28 | AACCTGGGAG | 708 | TRIM52 | TTGGAGATCT | 824 | NDUFA4 |
| TTTCAGAGAG | 728 | SRP9 | ACCTCCACTG | 698 | KRTDAP | CTCCTCACCT | 810 | BAK1 |
| ACTCCAAAAA | 713 | RPS15 | AGTGCAGGGA | 692 |  | TGCATCTGGT | 810 | HSPA5 |
| CGCTGGTTCC | 702 | RPL11 | AACGCGGCCA | 690 | MIF | GGAAAAAAAA | 810 | HGF |
| AACTAAAAAA | 687 | EPRS | TTGGAGATCT | 686 | NDUFA4 | GAAACAAGAT | 809 | PGK1 |
| TACTAGTCCT | 684 | HSP90AA1 | CTCCTCACCT | 682 | BAK1 | CTGACCTGTG | 799 | HLA-B |
| TTGTAATCGT | 679 | OAZ1 | GCAAAACCCC | 677 | FCF1 | TTGCTGACTT | 797 | COL6A1 |
| TACAGTATGT | 673 | GLUL | AATATGTGGG | 668 | COX6C | AGGTCAGGAG | 797 | KIAA0101 |
| CCCGTCCGGA | 672 | RPL13 | CGCTGGTTCC | 665 | RPL11 | TTACCTCCTT | 796 | FAM128A |
| ATGGCTGGTA | 672 | RPS2 | GTGCTGAATG | 663 | MYL6 | TGAAATAAAA | 793 | PLAC9 |
| ATTATTTTTC | 671 | RPL7 | TGTGATCAGA | 659 | ATP5L | ATCCTTGCTG | 791 | CSTA |
| GTAAGTGTAC | 670 |  | CAGCTATTTC | 652 | FABP5 | GCCTTCCAAT | 791 | DDX5 |
| CCCTGGGTTC | 665 | FTL | AGGTCTGCCA | 651 | AKR1C1 | CCATTGCACT | 786 | SLC2A6 |
| CAAGACCAGT | 659 | GSTA2 | AGGTCCTAGC | 650 | GSTP1 | AGCAGATCAG | 782 | S100A10 |
| CCTGCTGCAG | 653 | MUC5B | TTTGTAGAGG | 648 | PKP1 | ATTATTTTTC | 781 | RPL7 |
| TGGTGTTGAG | 645 | RPS18 | CCCCAGTTGC | 647 | CAPNS1 | AGAGGTGTAG | 774 |  |
| CCTCCAGCTA | 640 | KRT8 | AATCCTGTGG | 641 | RPL8 | AAGGAGCAAG | 750 | CES1 |
| AGAAAAAAAA | 639 | CRADD | AGAACCTTCC | 640 | HLA-A | CAATAAACTG | 743 | EIF1 |
| GTTCTGGTTT | 632 | ATPIF1 | TGAAAAAAAA | 636 | NCAM1 | AGCTCTCCCT | 742 | RPL17 |
| AGCTCTCCCT | 632 | RPL17 | CTTAATCCTG | 633 | SLC38A2 | AGAACCTTCC | 742 | HLA-A |
| GCCTGTATGA | 626 | RPS24 | TGTGCTAAAT | 633 | RPL34 | GACAAAAAAA | 735 | TWIST2 |
| GGGTTGGCTT | 622 |  | AGCAGATCAG | 631 | S100A10 | CGCTGGTTCC | 730 | RPL11 |
| AACCCGGGAG | 618 | ZNF669 | CCCGTCCGGA | 625 | RPL13 | CTCAACATCT | 726 | RPLP0 |
| GCCGAGGAAG | 614 | RPS12 | CTGTTGATTG | 620 | HNRNPA1 | ATTGTTTATG | 710 | TRMT5 |
| GTAGGGGTAA | 610 |  | AGCTCTCCCT | 620 | RPL17 | GGAATGTACG | 705 | ATP5G3 |
| GGGAAGCAGA | 609 | F11R | GTTCGTGCCA | 618 | RPL35A | TTTCCTCTCA | 698 | SFN |
| CCCCAGTTGC | 608 | CAPNS1 | TCTTAATGAA | 617 | EIF4A2 | ACTTACCTGC | 690 | COX6B1 |
| CGATTCTGGA | 606 | UFC1 | TTACCTCCTT | 612 | FAM128A | TTACCATATC | 682 | RPL39 |
| GCGAAACCCC | 604 | COQ10B | TGCCTCACCT | 608 | PLUNC | CCCCAGTTGC | 682 | CAPNS1 |
| ATAAACATTT | 602 | LRRC50 | ACAGCGGCAA | 604 | DSP | CAATTAAAAG | 676 | XBP1 |
| ATTTTCTTAA | 600 | RSPH9 | CTAAAAAAAA | 602 | TSKS | CCCAAGCTAG | 676 | HSPB1 |
| TTGTTGTTGA | 600 | CALM2 | CAGGCCCCAC | 601 | S100A11 | GCACAAGAAG | 671 | CENPL |
| AACTAACAAA | 599 | RPS27A | GGCCCAGGCC | 599 | ALDH3A1 | CCTCCAGCTA | 669 | KRT8 |
| GCATTTAAAT | 597 | EEF1B2 | CCCCAGCCAG | 598 | RPS3 | TTTGAAATGA | 667 | SAT1 |
| GACATCAAGT | 596 | KRT19 | GGCTGGGGGC | 590 | PFN1 | AACGCGGCCA | 666 | MIF |
| GGAAAGATGC | 586 | C9orf116 | GCACAAGAAG | 588 | CENPL | GACTCTTCAG | 661 | SERPINA3 |
| AGCTCTTGGA | 584 | SELENBP1 | TAAGTGGAAT | 588 | YWHAZ | GGCAAGAAGA | 658 | RPL27 |
| TAGACTAGCA | 568 | TSPAN3 | TTATAAAAGA | 585 |  | GGTGAGACAC | 658 | SLC25A6 |
| GTTATGGCTG | 563 | CYP4B1 | TTACCATATC | 577 | RPL39 | CCTCAGGATA | 656 | TGFA |
| GTCTGGGGCT | 561 | TAGLN2 | CCCTGGGTTC | 577 | FTL | ACCTGTATCC | 654 | IFITM3 |
| TCTTAATGAA | 560 | EIF4A2 | TGGTGTTGAG | 575 | RPS18 | TTCACTGTGA | 649 | LGALS3 |
| CTGACCTGTG | 560 | HLA-B | CTGTCACCCT | 571 | SPRR1A | AACCCGGGAG | 641 | ZNF669 |
| AGGTCAGGAG | 559 | KIAA0101 | GCGAAACCCC | 570 | COQ10B | GCTTTGATGA | 640 | EPHX1 |
| AGGTGGCAAG | 558 | LOC644075 | TGTATAAAAA | 568 | HSP90B1 | AGAACAAAAC | 637 | PRDX1 |
| AAGGAGCAAG | 557 | CES1 | GAAATCCAAA | 563 |  | TGTATAAAAA | 637 | HSP90B1 |
| CAATGTGTTA | 554 | NDUFA1 | ACTAACACCC | 562 |  | GAGACTCCTG | 633 | SLC2A1 |
| GTTGTCTTTG | 547 | NSMCE4A | ATTGTTTATG | 561 | TRMT5 | GAAGTCGGAA | 625 |  |
| TGTACCTGTA | 544 | TUBA1B | TTTCAGAGAG | 557 | SRP9 | TTATGTTTAA | 622 | LUM |
| CCCCCTGGAT | 542 | S100A6 | GAAACAAGAT | 554 | PGK1 | AACGAGGAAT | 619 |  |
| GCTAGGTTTA | 540 |  | GTGACCTCCT | 554 | COX8A | GTGACCTCCT | 615 | COX8A |
| GAAATGATGA | 536 | PFDN5 | AGGTGGCAAG | 554 | LOC644075 | ACCCGCCGGG | 612 | TNNC2 |
| AAGATTGGTG | 535 | CD9 | AGAACAAAAC | 550 | PRDX1 | AGGTCCTAGC | 602 | GSTP1 |
| CCACTGCTCT | 532 |  | ATTTGAGAAG | 547 | RAD23B | AATATGTGGG | 602 | COX6C |
| CTCCCAGCCA | 532 | SFTPA2B | AAGGAGATGG | 547 | RPL31 | GGGGAAATCG | 599 | TMSB10 |
| TTTGCACCTT | 531 | CTGF | GGGTTGGCTT | 542 |  | AAATAAAGAA | 598 | MGST1 |
| AAATAAAGAA | 531 | MGST1 | GACTCTGGTG | 542 | RPS15A | GCCTGTATGA | 596 | RPS24 |
| AATCCTGTGG | 526 | RPL8 | TACAGAGGGA | 541 | ZFAND5 | CACTTGCCCT | 589 | ACSS2 |
| TTTGCAAATA | 523 | PDZRN3 | CAATGTGTTA | 536 | NDUFA1 | CAATGTGTTA | 588 | NDUFA1 |
| AAGAAGATAG | 521 | RPL23A | GGGAAGCAGA | 530 | F11R | GGTGGTGTCT | 585 | GPX2 |
| AGAACAAAAC | 520 | PRDX1 | CCCAAGCTAG | 527 | HSPB1 | GTAATCCTGC | 583 |  |
| TTTGCTTTTG | 517 | AQP3 | CTGGGTGCCT | 527 | PSMB4 | AAGATTGGTG | 580 | CD9 |
| ACAGTGGGGA | 515 | PTGES3 | TACATAATTA | 523 | NCRNA00084 | GGTCCAGTGT | 579 | PGAM1 |
| TTTAACGGCC | 513 |  | TGAAATAAAA | 519 | PLAC9 | CTGTTGATTG | 569 | HNRNPA1 |
| AAGAAAACCT | 511 | AGR3 | AATTCAATTA | 517 | EIF4G1 | CTGTTGGTGA | 569 | RPS23 |
| CTTCCAGCTA | 511 | ANXA2 | TAAGTAGCAA | 514 | ITM2B | CCACAGGAGA | 569 | PERP |
| AAGTTGCTAT | 511 | PSAP | CCTGTAGTCC | 512 | MAFF | CTAGCCTCAC | 565 | ACTG1 |
| GCAAAAAAAA | 511 | RCE1 | GCGGAGGTGG | 504 | IGHG1 | TCGTCTTTAT | 561 | RPS7 |
| CAATAAACTG | 510 | EIF1 | TTTAACGGCC | 501 |  | GGCTTTACCC | 561 | EIF5A |
| CCCCAGCCAG | 509 | RPS3 | TGCCCTCAAA | 499 | LCN2 | CTGTACAGAC | 552 | TUBB2C |
| TTTATTGAAA | 508 | C6orf26 | TGCATCTGGT | 496 | HSPA5 | AGGTCTGCCA | 541 | AKR1C1 |
| CCTGTAGTCC | 506 | MAFF | GTGACCACGG | 491 | GRIN2C | AAGAAGATAG | 537 | RPL23A |
| TTTTTCAAGA | 501 | CXCL17 | TACTAATAAA | 487 | ROMO1 | CCTATCAGTA | 537 | MSMB |
| ACTTACCTGC | 499 | COX6B1 | CCTCAGGATA | 483 | TGFA | TTATAAAAGA | 536 |  |
| GGATGTTGCA | 493 | C20orf85 | TTGCTCAAAA | 479 |  | GAATTTTATA | 534 | TSPO |
| GAATTAACAT | 483 | YWHAE | CTCAACATCT | 476 | RPLP0 | CTAACTAGTT | 531 |  |
| CCTGCTTGTC | 483 | WFDC2 | GAGGCCAAGA | 470 | GSTA1 | CGCCGACGAT | 528 | IFI6 |
| TGTGATCAGA | 480 | ATP5L | CAAACTAACC | 466 | IGHG1 | GACTCTGGTG | 521 | RPS15A |
| ATAATAAAAG | 479 | CXCL3 | TGGAAATGAC | 463 | COL1A1 | TTCATTATAA | 518 | PTMA |
| CAGGCCCCAC | 478 | S100A11 | GCCTCCTCCC | 455 | EIF3K | CCCCCTGGAT | 517 | S100A6 |
| ATCTTTTAAA | 477 | CYB5A | TACAGTATGT | 454 | GLUL | CAAACTAACC | 516 | IGHG1 |
| TTACACCTGT | 477 | CETN2 | CAATAAAATT | 453 | TP63 | GACATCAAGT | 516 | KRT19 |
| TCTGTTTATC | 476 | SRP14 | TGTGGGAAAT | 453 | SLPI | CTAGCTTTTA | 515 |  |
| CTCCTCACCT | 476 | BAK1 | GAGCAGCGCC | 453 | S100A7 | TACATAATTA | 513 | NCRNA00084 |
| CGCCGCCGGC | 470 | RPL35 | TCAAAAAAAA | 452 | WIBG | AACTAATACT | 513 | C17orf45 |
| TCCAAGTCCG | 467 | TMEM190 | TAATGGTAAC | 451 | COX5A | ACCCTTGGCC | 510 | HNRNPM |
| GCAAAACCCC | 466 | FCF1 | GGAATAAATT | 449 | CYC1 | GAACACATCC | 509 | RPL19 |
| GACTGTGCCA | 462 | DYNLL1 | ATGAGCTGAC | 448 | CSTB | GTTGTCTTTG | 506 | NSMCE4A |
| TCAATAAATG | 459 | SCPEP1 | TTGGCCAGGA | 445 | POLR2J2 | TGTGGGAAAT | 505 | SLPI |
| GAACACATCC | 455 | RPL19 | CTGTACAGAC | 444 | TUBB2C | GTGTGGGGGG | 503 | JUP |
| GTGACCACGG | 446 | GRIN2C | TTAAAAAAAA | 444 | GSDMB | ACTGGGTCTA | 499 | NME1 |
| GCGCGGGGGC | 445 |  | CTGTTGGTGA | 444 | RPS23 | GCAAAACCCC | 499 | FCF1 |
| TGAGGGAATA | 443 | TPI1 | TCATTTTCCA | 443 | SLC6A8 | GGTCAGTCGG | 496 |  |
| CCTGGGAAGT | 441 | MUC1 | GGCAAGAAGA | 443 | RPL27 | TTTTTAATGT | 493 | H3F3A |
| TGAAAAAAAA | 441 | NCAM1 | AAGAAGATAG | 439 | RPL23A | GACTCACTTT | 491 | PPIB |
| TGTGCTAATA | 432 | PRKAR1A | TTTGGTTTTC | 439 | COL1A2 | ATTAAGAGGG | 487 | MAN2C1 |
| CAACATAATA | 431 | FAM154B | TGTAATCAAT | 438 | HNRNPA1 | ACACAGCAAG | 486 | EMID2 |
| CATTTGTAAT | 431 |  | CCTATCAGTA | 436 | MSMB | TTTCAGAGAG | 483 | SRP9 |
| TGTAGTTTGA | 429 | SKP1 | TTATGTTTAA | 433 | LUM | ATCAAGGGTG | 482 | RPL9 |
| AAATAAAAGA | 427 | SSR3 | GAGACTCCTG | 432 | SLC2A1 | GCGAAACCCC | 480 | COQ10B |
| TACCCTAGAA | 424 | PTPRJ | CCTATTTACT | 431 | COX4I1 | TTTCTAGTTT | 479 | LAPTM4A |
| GAAGCAGGAC | 420 | EFEMP2 | GCCTGCTGGG | 429 | GPX4 | AGTTTCCCAA | 477 | TMED9 |
| GAATGATTTC | 418 | C5orf32 | TACCCTAGAA | 429 | PTPRJ | TGTAATCAAT | 474 | HNRNPA1 |
| CTCATAGCAG | 417 | TPT1 | TGTTCTGGAG | 424 | GJA1 | GCCGTTCTTA | 472 |  |
| CACCTGTCAT | 417 | SLC44A4 | AAGGAGCAAG | 421 | CES1 | CCTATTTACT | 469 | COX4I1 |
| AAATTAAAAA | 417 | WDR70 | TTTTTAATGT | 419 | H3F3A | TAATGGTAAC | 468 | COX5A |
| TGTAATCAAT | 413 | HNRNPA1 | TGTATGTAAA | 419 | KLK10 | TACAGTATGT | 468 | GLUL |
| GCTTTTAAGG | 411 | RPS20 | TTCACTGTGA | 416 | LGALS3 | TCAATAAATG | 464 | SCPEP1 |
| TGAAGAGTCT | 411 | TTLL10 | TATGACTTAA | 416 | RAC1 | CCGTCCAAGG | 463 | RPS16 |
| TGAAAGTGTG | 411 | HSPH1 | GCCTGTATGA | 414 | RPS24 | CCAGGGGAGA | 461 | IFI27 |
| AGGGAGGCAG | 409 | SCGB1A1 | GCCGAGGAAG | 413 | RPS12 | CAAACCATCC | 460 | KRT18 |
| GACCCAAGAT | 409 | PIGR | GGTCCAGTGT | 412 | PGAM1 | TTCTTGTTTT | 460 | PRNP |
| GCAAGAAAGT | 407 | HBB | GCGAAACCCT | 412 | DKFZp761E198 | GTACGTATTC | 457 | IGJ |
| GGCAAGAAGA | 407 | RPL27 | GCAAAACCCT | 411 |  | AATTCAATTA | 456 | EIF4G1 |
| TTTCTAGTTT | 405 | LAPTM4A | TAAGGCTTAA | 410 | KLK10 | CTCATAGCAG | 454 | TPT1 |
| ATCAAGGGTG | 404 | RPL9 | TTTCTAGTTT | 410 | LAPTM4A | TAGAAAAATA | 451 | GPI |
| AATATGTGGG | 403 | COX6C | TAGAAAAATA | 408 | GPI | GGTTTGGCTT | 449 | UQCRH |
| AACCCAGGAG | 401 | GK5 | GGTTTGGCTT | 406 | UQCRH | AATGGATGAA | 448 |  |
| AAGAAAACTG | 399 | KIAA1522 | TAAAATGTAT | 404 | DSG3 | GTGTGTTTGT | 447 | TGFBI |
| TGCCCTCAGA | 397 |  | CCACTGTACT | 404 | ZNF726 | CACTTCAAGG | 447 | LY6E |
| GTATTGGCCT | 397 | TM9SF2 | GTGATGGTGT | 403 | XRCC6 | TCTTAATGAA | 446 | EIF4A2 |
| ATGTAAAAAA | 396 | LYZ | ATGTGAAGAG | 402 | SPARC | TAAGTAGCAA | 444 | ITM2B |
| ACCTCAGGAA | 396 | HDLBP | ATGAAAAGAA | 398 | MAL2 | CCTGTAGTCC | 443 | MAFF |
| ATTCCTTTAA | 394 | LRP11 | TGATTGTGAT | 398 | NTS | TGGTACACGT | 442 | NDUFA13 |
| GAATTTTATA | 392 | TSPO | TGATAATTCA | 398 | USMG5 | TTTGCACCTT | 440 | CTGF |
| GCTTTATTTG | 391 | ACTB | CGCCGACGAT | 398 | IFI6 | TTTCCTGCTC | 438 | SPRR3 |
| CTAGCTTTTA | 390 |  | ACTTTTTAAA | 397 |  | TGGGTGAGCC | 436 | CTSB |
| TGGCTGGGAA | 389 | VAMP8 | CTTCCAGCTA | 393 | ANXA2 | ATCGCTTTCT | 436 | APP |
| GACAAAAAAA | 388 | TWIST2 | AAATAAAGCA | 392 |  | AGCCTTTGTT | 436 | SERPINH1 |
| AGGGTGTTTT | 387 | DYRK1A | GAACACATCC | 391 | RPL19 | AACCCAGGAG | 434 | GK5 |
| AGACCCACAA | 387 |  | AGAGTCATAC | 391 | DSC2 | TGATAATTCA | 434 | USMG5 |
| GTGACCTCCT | 385 | COX8A | GCTTGAATAA | 391 | AKR1B10 | GTTCCCTGGC | 430 | FAU |
| TTTATTGAAT | 385 | CD164 | TAAATAATTT | 390 | HSPE1 | AAAGTCTAGA | 430 | CCND1 |
| TTTTCTGAAA | 385 | TXN | TTTGAAATGA | 389 | SAT1 | GCTTGAATAA | 429 | AKR1B10 |
| GTTCGTGCCA | 385 | RPL35A | TTGGTCAGGC | 383 | MARVELD2 | GTCCCTGCCT | 427 | GSTM1 |
| TTACCATATC | 384 | RPL39 | AATAAATGGA | 382 | C14orf2 | ATCTTGTTAC | 425 | FN1 |
| ATGAAACTTC | 384 | ASNS | CTTCTACTAA | 381 | FAM129B | TTCGGTTGGT | 423 |  |
| TTCATTATAA | 383 | PTMA | AACAGTCAAA | 381 | PKP3 | GCCTGCTGGG | 422 | GPX4 |
| CTGTTGGTGA | 383 | RPS23 | TCTGTTTATC | 380 | SRP14 | CTTAATCCTG | 421 | SLC38A2 |
| TCCGTGGTTG | 383 | BASP1 | ACCTGGAGGG | 378 | SBSN | TTACTAAATG | 420 | CANX |
| CTCAACATCT | 382 | RPLP0 | CTGACCTGTG | 373 | HLA-B | TTTTCTGAAA | 420 | TXN |
| CTAAAAAAAA | 381 | TSKS | AAAATAAAGC | 373 | NEIL2 | TGCCCTCAAA | 417 | LCN2 |
| TGAAATAAAA | 377 | PLAC9 | GTAAATATGG | 373 | DST | TTTAACGGCC | 412 |  |
| TGCATCTGGT | 376 | HSPA5 | GTTCCCTGGC | 372 | FAU | AAAAGAAACT | 409 | PABPC1 |
| TCAAAAAAAA | 372 | WIBG | CTCATAGCAG | 370 | TPT1 | ACAACTCAAT | 407 | BRI3 |
| TTAAAAAAAA | 371 | GSDMB | GCTTTATTTG | 367 | ACTB | TTTGTTAAAA | 405 | DDIT4 |
| CATTTGTCAA | 369 | SNTN | GGGGAAATCG | 367 | TMSB10 | TGGCCCCAGG | 403 | APOC1 |
| TACTAATAAA | 368 | ROMO1 | TTGGCAGCCC | 366 |  | AGTCTGATGT | 401 | C7orf59 |
| GTGTAATAAG | 365 | HNRNPA2B1 | GGGAAGGGAC | 361 | PPP2R1B | CAACTAATTC | 401 | CLU |
| GCAGAGGATG | 365 | C11orf88 | GAGAAACCCC | 361 | NCAPD2 | TCGAAGCCCC | 400 |  |
| TTCTGACATT | 363 | CCDC17 | GGGACGAGTG | 361 | TM4SF1 | GAATTAACAT | 399 | YWHAE |
| TTGGTTTTTG | 362 | CXCL6 | AGTCTGATGT | 360 | C7orf59 | CTAAAAAAAA | 396 | TSKS |
| GCCGTGTCCG | 361 | RPS6 | AGAATCGCTT | 360 | COPA | ATGATGATGA | 396 | SLC25A5 |
| CAGCAGAAGC | 361 | SERF2 | ATGTAAAAAA | 359 | LYZ | AAGGAACTTG | 395 | CMTM4 |
| ACAAAAACTA | 359 |  | GTTGTCTTTG | 358 | NSMCE4A | GCCTCCTCCC | 394 | EIF3K |
| TAATAAAGAA | 357 | KRT15 | CTCAGGAAAT | 357 | UCRC | TTGGTCAGGC | 390 | MARVELD2 |
| TAGATGTGAT | 357 | MORN2 | ATAGTAGCTT | 356 | FSCN1 | GAAATGTAAG | 389 | PCBP2 |
| CAACTTAGTT | 357 | MYL12B | CTAGCCTCAC | 355 | ACTG1 | TTGGAACAAT | 388 | ARHGAP19 |
| GCCAGTCTGT | 356 |  | AATCTTGTTT | 354 | DMKN | CGGCTGAATT | 387 | PGD |
| ATACTGTCAG | 356 | TMEM59 | TGGGGAGAGG | 354 | S100A14 | AATAAAGGCT | 387 | RHOC |
| GGGGTCAGGG | 354 | PYGB | GAAATAAAGT | 352 | NAMPT | GGGTTGGCTT | 384 |  |
| CTCGCGCTGG | 352 | CLDN3 | CAGGTTTCAT | 352 | CXCL14 | CTGATCTGTG | 382 | HLA-B |
| CCAGGAGGAA | 352 | HSPA8 | TAAAATGTTT | 349 | DSG1 | AACCTGGGAG | 378 | TRIM52 |
| TGCAGATTGC | 351 | PROM1 | ACAGTGGGGA | 347 | PTGES3 | TAAACTGTTT | 376 | RPS14 |
| TTGTCTGCCT | 351 | RHBDD2 | CCACTGCATT | 346 |  | ATGTCTTTTC | 373 | IGFBP4 |
| TGCCTGCACC | 350 | CST3 | GCTTTGATGA | 345 | EPHX1 | CTTTGAGTCC | 366 | SCGB1A1 |
| GTAATGTTTT | 348 | LOC390205 | TTGACACTTT | 345 | PIGY | AATAGAAATT | 363 | SPP1 |
| TACAGAGGGA | 348 | ZFAND5 | TAACTTGTGA | 342 | ITGAV | TAACCAATCA | 362 | RAB5C |
| TTACTAAATG | 347 | CANX | AAAAAACCCA | 342 | ENSA | ATAGTAGCTT | 361 | FSCN1 |
| GTGGAGGAGG | 347 | SPTA1 | GCCTTAACAA | 336 | NAMPT | GCGAAACCCT | 361 | DKFZp761E198 |
| ATGTTTTGTA | 346 | FLJ22167 | GTGAAACCTC | 335 | BFAR | AGGTGGCAAG | 357 | LOC644075 |
| GTTACGAAAG | 345 | C9orf24 | GGCTTTACCC | 335 | EIF5A | TATGACTTAA | 357 | RAC1 |
| AGAACCTTCC | 344 | HLA-A | TAATAAAGAA | 335 | KRT15 | CTTCCAGCTA | 356 | ANXA2 |
| GTCCAGGTGA | 343 | RSPH4A | ACTGGGTCTA | 334 | NME1 | CTCTAAGAAG | 355 | C1QA |
| GGCTGTATTT | 342 | CDH29 | GAAATAAAAG | 334 | EIF3B | GAGATAAATG | 354 | LY6D |
| TAATGGTAAC | 341 | COX5A | TCGAAGCCCC | 333 |  | GCCTACCCGA | 354 | TACSTD2 |
| GCAAAACCCT | 340 |  | TGCTAAAAAA | 332 | MYH9 | ACCTTTACTG | 353 | TFRC |
| AAAATAAAAA | 340 | PPOX | TAGGGCAATC | 332 | SUMO2 | TGTTCTGGAG | 353 | GJA1 |

^1^The most abundant 300 unique tags from the mean of 14 bronchial epithelial libraries.

^2^Average normalized tag counts, expressed as tags-per-million (TPM) for 14 bronchial epithelial libraries.

^3^Tag-to-gene mapping according to SAGE Genie, “Best Gene for Tag”, September 17, 2009 version. No entry is given for tags that map to transcript sequences within the databases of Mitochondria, Full-Length Set, UniGene Consensus, or Unclustered ESTs, all of which can be determined by manual mapping of individual tags.

^4^The most abundant 300 unique tags from the mean of the five carcinoma-in-situ libraries.

^5^Average normalized tag counts (TPM) for five carcinoma-in-situ libraries.

^6^The most abundant 300 unique tags from the mean of the six invasive cancer libraries.

^7^Average normalized tag counts (TPM) for six invasive cancer libraries.
