# Supplementary material for: Transcriptome Profiles of Carcinoma-in-Situ and Invasive Non-Small Cell Lung Cancer as Revealed by SAGE
Source: PLoS One. 2010 Feb 11;5(2):e9162. doi: 10.1371/journal.pone.0009162 (PMC2820080; doi:10.1371/journal.pone.0009162)
Supplement: Table S2 — Up-regulated gene expression changes in common between carcinoma-in-situ and precancerous lesions relative to bronchial epithelium. (0.29 MB DOC) [file pone.0009162.s002.doc]

**Table S2. Up-regulated gene expression changes in common between carcinoma-in-situ and precancerous lesions relative to bronchial epithelium.**

| **Tag^1^** | **BE Mean^2^** | **CIS Mean^3^** | **SCC Mean^4^** | **PC Av^5^** | **Gene Symbol^6^** |
| --- | --- | --- | --- | --- | --- |
| AAAGAGAAAG | 4 | 119 | 137 | 43 | ADM |
| AATTGCAAGA | 8 | 52 | 13 | 216 | APOBEC3A |
| TGTAGGTCAT | 14 | 81 | 108 | 50 | ARL4C |
| GCCTTAAAAA | 24 | 172 | 101 | 169 | ARL8B |
| TGAAAACTTT | 11 | 42 | 23 | 49 | ARL8B |
| ACATCGTTGT | 16 | 78 | 35 | 54 | ATP11B |
| GCAAGACCCC | 33 | 100 | 61 | 107 | C11orf76 |
| TATTTGTTGA | 14 | 52 | 18 | 98 | C3orf57 |
| TCCTGTAAAG | 22 | 111 | 144 | 293 | CAV1 |
| CGTTTAATCA | 16 | 135 | 106 | 60 | CCR2 |
| ATTTATTAAT | 7 | 44 | 47 | 175 | CD109 |
| ATATGTATAT | 57 | 206 | 120 | 384 | CD44 |
| AAGATTGGGG | 22 | 68 | 67 | 122 | CD44 |
| TGCAGATATT | 2 | 89 | 60 | 54 | CDKN3 |
| CGGGAGCGCT | 8 | 90 | 7 | 95 | CNFN |
| TGGAAATGAC | 9 | 463 | 2140 | 4521 | COL1A1 |
| ACCAAAAACC | 6 | 132 | 1044 | 862 | COL1A1 |
| TTTGGTTTTC | 2 | 439 | 1269 | 3047 | COL1A2 |
| CCACAGGGGA | 1 | 61 | 283 | 236 | COL3A1 |
| TTGCTGACTT | 17 | 124 | 797 | 631 | COL6A1 |
| GTGCTGATTC | 39 | 191 | 159 | 270 | COL7A1 |
| CGTGGGACAC | 0 | 95 | 14 | 67 | CRCT1 |
| ATCCTTGCTG | 68 | 1693 | 791 | 2038 | CSTA |
| CAGGTTTCAT | 3 | 352 | 195 | 85 | CXCL14 |
| ACGAGAGTGT | 0 | 72 | 3 | 131 | DEFB103A |
| TAAACCAAAT | 1 | 126 | 12 | 215 | DEFB4 |
| AGAGTCATAC | 2 | 391 | 40 | 86 | DSC2 |
| TAAAATGTTT | 94 | 349 | 128 | 302 | DSG1 |
| TAAAATGTAT | 5 | 404 | 71 | 158 | DSG3 |
| ACAGCGGCAA | 134 | 604 | 288 | 416 | DSP |
| GTAAATATGG | 25 | 373 | 196 | 102 | DST |
| GCACAGGCCA | 14 | 68 | 85 | 134 | EGFL7 |
| GCTGGGAGGG | 17 | 62 | 87 | 119 | EHD2 |
| TAATTTGCAT | 14 | 90 | 82 | 574 | EMP1 |
| AAACCAAAAA | 23 | 84 | 99 | 177 | ENG |
| AGAATAAAAA | 21 | 102 | 101 | 144 | ENO1 |
| AGGGCTGCAG | 12 | 149 | 39 | 54 | EPHA1 |
| TTTGAGAATA | 17 | 57 | 26 | 164 | EVI2B |
| GTGATGGGCT | 16 | 63 | 29 | 55 | EVPL |
| CAGCTATTTC | 52 | 652 | 323 | 309 | FABP5 |
| GCCCACACAG | 10 | 149 | 79 | 362 | FGFBP1 |
| ACATTTCATC | 12 | 59 | 55 | 41 | FOSL2 |
| GTAAGATTAG | 10 | 119 | 85 | 55 | FRMD6 |
| ATAGTAGCTT | 4 | 356 | 361 | 127 | FSCN1 |
| AGATAATGTT | 32 | 109 | 110 | 173 | FURIN |
| TTTTAACAAA | 17 | 57 | 46 | 129 | FYN |
| TGTTCTGGAG | 7 | 424 | 353 | 154 | GJA1 |
| ACACTTCTTT | 10 | 49 | 91 | 117 | GNG11 |
| ACATTCTTTT | 48 | 719 | 970 | 199 | GPNMB |
| AGCTTCTACC | 0 | 1006 | 99 | 283 | HCG9 |
| TAGATTTCAA | 20 | 76 | 90 | 62 | HIF1A |
| ATTTGTCCCA | 91 | 302 | 245 | 391 | HMGA1 |
| CGTGGGTGGG | 24 | 89 | 186 | 449 | HMOX1 |
| CCCAAGCTAG | 163 | 527 | 676 | 763 | HSPB1 |
| CAGATGCAAA | 39 | 165 | 160 | 126 | IFI27L2 |
| ATGTCTTTTC | 50 | 205 | 373 | 230 | IGFBP4 |
| GAAATAAAGC | 62 | 38019 | 14716 | 568 | IGHG1 |
| AAACCCCAAT | 18 | 4949 | 1692 | 280 | IGL@ |
| AGAAATAAGG | 8 | 49 | 19 | 41 | IPPK |
| ATTAGAAATT | 6 | 98 | 57 | 74 | ITGA6 |
| TCTCTTTAAT | 0 | 40 | 9 | 69 | IVL |
| GTGTGGGGGG | 108 | 1061 | 503 | 474 | JUP |
| TAAGGCTTAA | 29 | 410 | 98 | 195 | KLK10 |
| TGTATGTAAA | 27 | 419 | 71 | 148 | KLK10 |
| CACTCAATAA | 0 | 88 | 19 | 132 | KLK6 |
| TGGCTACTTA | 9 | 44 | 31 | 98 | KPNA2 |
| TAGCTGAGAC | 9 | 46 | 39 | 45 | KPNA2 |
| GATGTGCACG | 1 | 141 | 30 | 419 | KRT14 |
| CTTCCTTGCC | 224 | 10219 | 5293 | 4108 | KRT17 |
| GCCCCTGCTG | 141 | 1083 | 1618 | 844 | KRT5 |
| TTTTATCCTT | 16 | 86 | 96 | 86 | KRT5 |
| AAAGCACAAG | 17 | 2924 | 1822 | 4982 | KRT6A |
| CGAATGTCCT | 1 | 230 | 78 | 260 | KRT6B |
| GAAGCACAAG | 0 | 122 | 63 | 508 | KRT6C |
| GCCAGGAGCT | 7 | 156 | 76 | 103 | LAD1 |
| GTTTTTTTTA | 12 | 43 | 42 | 88 | LEPRE1 |
| GCCCCCAATA | 62 | 283 | 930 | 1466 | LGALS1 |
| TAAACCTGCT | 15 | 91 | 63 | 390 | LGALS7B |
| TTTCAATAGA | 11 | 51 | 85 | 93 | LIMD2 |
| GGAGGGGGCT | 29 | 126 | 152 | 136 | LMNA |
| TGGGTCTGAA | 31 | 106 | 66 | 141 | LRRC8A |
| GTGGAATAAA | 13 | 48 | 105 | 60 | LTBP2 |
| TTATGTTTAA | 15 | 433 | 622 | 1571 | LUM |
| TGTTTTATAA | 11 | 41 | 32 | 78 | MAD2L1 |
| TAAAATAAGG | 13 | 55 | 75 | 52 | MAFB |
| GATGGAATGT | 11 | 56 | 55 | 90 | MAL2 |
| CAAATAAAAG | 28 | 117 | 75 | 130 | MALL |
| CCCCCTGCCC | 33 | 118 | 117 | 110 | MAP7D1 |
| GTGCTATTAT | 9 | 56 | 41 | 65 | MCL1 |
| TAAGTGAACA | 5 | 71 | 68 | 634 | MFAP1 |
| TGCAGTCACT | 1 | 82 | 223 | 526 | MMP1 |
| GGAAATGTCA | 5 | 111 | 311 | 212 | MMP2 |
| TTCTATTTCA | 40 | 137 | 242 | 274 | MSN |
| TGCTAAAAAA | 84 | 332 | 242 | 306 | MYH9 |
| GCCTTAACAA | 66 | 336 | 258 | 298 | NAMPT |
| AGGGTGGTGA | 2 | 56 | 8 | 98 | NCCRP1 |
| TACCTGCAAA | 4 | 68 | 31 | 72 | NCF1 |
| CCAGGGCAAC | 29 | 167 | 50 | 392 | NCRNA00084 |
| GGCTGGTCTG | 26 | 112 | 168 | 184 | NCRNA00152 |
| GGAAGGGGAG | 47 | 140 | 128 | 158 | NFKB2 |
| CCCTTGACCC | 16 | 62 | 30 | 54 | PCDH1 |
| TTGAATCCCC | 42 | 952 | 115 | 193 | PI3 |
| TTTGTAGAGG | 15 | 648 | 239 | 153 | PKP1 |
| AACAGTCAAA | 73 | 381 | 137 | 235 | PKP3 |
| AATAAAAGTG | 19 | 59 | 28 | 128 | PLCB3 |
| CAGAAGTGTC | 16 | 50 | 37 | 71 | PPM1F |
| AGCAGGGCTC | 41 | 205 | 223 | 214 | PPP1R14B |
| GGGAAGGGAC | 3 | 361 | 128 | 118 | PPP2R1B |
| TTCTTGTTTT | 77 | 267 | 460 | 300 | PRNP |
| GGGATGGAAG | 17 | 62 | 58 | 72 | PRR5 |
| GTTCTCTTTA | 8 | 42 | 18 | 116 | PUS7L |
| CCAATAAAGT | 28 | 83 | 176 | 214 | RBP1 |
| TTAACCCCTC | 65 | 221 | 312 | 365 | RNASE1 |
| AGAAATACCA | 60 | 210 | 235 | 280 | RPL22L1 |
| GTCACTGCCT | 18 | 63 | 52 | 70 | RPS6KA4 |
| TGGGGAGAGG | 114 | 354 | 282 | 912 | S100A14 |
| AGCAGGAGCA | 23 | 71 | 76 | 479 | S100A16 |
| GATCTCTTGG | 215 | 6836 | 2471 | 8390 | S100A2 |
| GAGCAGCGCC | 0 | 453 | 1041 | 492 | S100A7 |
| TACCTGCAGA | 87 | 4937 | 2039 | 6914 | S100A8 |
| GTGGCCACGG | 81 | 2184 | 2103 | 2584 | S100A9 |
| ACCTGGAGGG | 0 | 378 | 12 | 129 | SBSN |
| CTGAGGCCTG | 22 | 125 | 93 | 69 | SDC1 |
| CAATTTTGCA | 6 | 48 | 12 | 50 | SELL |
| TAAATAAAGA | 26 | 110 | 56 | 616 | SERPINB13 |
| CATTGTAAAT | 22 | 94 | 54 | 133 | SERPINB5 |
| TAAAAATGTT | 7 | 48 | 178 | 316 | SERPINE1 |
| TTTCCTCTCA | 63 | 1312 | 698 | 1473 | SFN |
| GTTATAATAC | 21 | 173 | 100 | 131 | SGK1 |
| GCAGGAAGTC | 2 | 145 | 30 | 230 | SLC16A7 |
| ATGATGATGA | 97 | 307 | 396 | 293 | SLC25A5 |
| CTTAATCCTG | 125 | 633 | 421 | 556 | SLC38A2 |
| GTGAAGTCTT | 18 | 97 | 101 | 59 | SLC3A2 |
| ACTCCATAGA | 14 | 42 | 26 | 58 | SMAP1 |
| GACTTTTAAA | 28 | 154 | 116 | 111 | SMC4 |
| ATGTGAAGAG | 18 | 402 | 2226 | 878 | SPARC |
| CTGTCACCCT | 4 | 571 | 61 | 416 | SPRR1A |
| CCCTTGAGGA | 0 | 307 | 65 | 924 | SPRR1B |
| ATGATCCCTG | 0 | 277 | 48 | 165 | SPRR2A |
| TTTCCTGCTC | 7 | 3458 | 438 | 5415 | SPRR3 |
| TTTTCCTTTT | 16 | 56 | 32 | 48 | SRL |
| TCACCAAAAA | 10 | 65 | 61 | 273 | STAB1 |
| TTTCATTGCC | 29 | 93 | 67 | 143 | TACC1 |
| ACTCAATAAA | 16 | 50 | 50 | 98 | TACC3 |
| GTGTGTTTGT | 36 | 148 | 447 | 455 | TGFBI |
| TCTCTGATGC | 17 | 78 | 259 | 255 | TIMP2 |
| GGGACGAGTG | 42 | 361 | 217 | 428 | TM4SF1 |
| GATTGGGGAT | 20 | 100 | 79 | 124 | TMEM106C |
| CCCCTTATTT | 10 | 50 | 40 | 46 | TMEM132A |
| GGAGAGGGCA | 15 | 70 | 59 | 56 | TMEM164 |
| TAAGCAGATA | 1 | 59 | 18 | 137 | TMPRSS11A |
| ACTTTTTGGC | 14 | 44 | 21 | 48 | TNRC6A |
| CAATAAAATT | 22 | 453 | 149 | 89 | TP63 |
| GCTGCCCTTG | 69 | 237 | 289 | 296 | TUBA1C |
| GAGGAGGGTG | 12 | 151 | 44 | 60 | TUBA4A |
| TTTCTTCCCT | 13 | 72 | 73 | 56 | TUFT1 |
| ATGTAGAGTG | 8 | 73 | 78 | 60 | TYMS |
| ACTCCTACTT | 15 | 62 | 58 | 59 | UPK1B |
| TTCTGCTCTT | 7 | 81 | 156 | 76 | VWF |
| AATGTGAGTC | 63 | 216 | 240 | 282 | YWHAG |
| TAAGTGGAAT | 183 | 588 | 329 | 695 | YWHAZ |
| CTTTATTCCA | 1 | 77 | 233 | 1719 |  |
| TTCGGTTGGT | 4 | 67 | 423 | 637 |  |
| TCAAAAGACC | 52 | 194 | 81 | 557 |  |
| TATTGTTACT | 27 | 119 | 19 | 391 |  |
| ACAGTGATGA | 6 | 232 | 60 | 341 |  |
| GTAGACACCT | 48 | 182 | 75 | 286 |  |
| GATACTGCCT | 0 | 198 | 88 | 227 |  |
| GGTCACTGAG | 17 | 143 | 74 | 223 |  |
| AACCCGGGAA | 19 | 948 | 62 | 217 |  |
| GCTTTTTTTG | 3 | 70 | 18 | 200 |  |
| TGCGCCTTTA | 42 | 162 | 100 | 173 |  |
| GCGGCCAGTA | 46 | 154 | 101 | 146 |  |
| CCACGAGGTG | 10 | 72 | 30 | 129 |  |
| ACTAATCGTT | 32 | 108 | 105 | 108 |  |
| GATCTGTGGC | 6 | 85 | 45 | 82 |  |
| CATTTCTTTT | 2 | 56 | 10 | 81 |  |
| GTAAAGACTG | 12 | 40 | 29 | 80 |  |
| ATCACCAAGT | 14 | 51 | 36 | 79 |  |
| CTTTCCCTCA | 1 | 41 | 14 | 76 |  |
| CTGCTAAAAG | 0 | 54 | 28 | 68 |  |
| AGTAGCCGTG | 9 | 40 | 42 | 68 |  |
| TGCTACGAAA | 15 | 55 | 56 | 67 |  |
| GGCGTTTAGA | 1 | 109 | 81 | 63 |  |
| TAAGTTGTCT | 18 | 84 | 35 | 56 |  |
| AGCGACAAAC | 16 | 121 | 108 | 48 |  |
| TTGCGTTGCG | 3 | 52 | 24 | 48 |  |
| GATTTCTTGG | 0 | 44 | 3 | 44 |  |
| AACATAATCT | 13 | 107 | 70 | 44 |  |
| TTAATTACAG | 12 | 56 | 39 | 41 |  |

^1^Tags with a three-fold or greater abundance in average normalized tag counts in both CIS and PC relative to BE; a minimal average abundance of 40 TPM in both CIS and PC (190 tags in total).

^2^Average normalized tag counts, expressed as tags per million (TPM) for 14 bronchial epithelial libraries.

^3^Average normalized tag counts (TPM) for five carcinoma-in-situ libraries.

^4^Average normalized tag counts (TPM) for six invasive cancer libraries.

^5^Average normalized tag counts (TPM) for two precancerous libraries.

^6^Tag-to-gene mapping was according to SAGE Genie, “Best Gene for Tag”, September 17, 2009 version. No entry is given for tags that map to transcript sequences within the database of Unclustered ESTs.
