# Supplementary material for: Transcriptome Profiles of Carcinoma-in-Situ and Invasive Non-Small Cell Lung Cancer as Revealed by SAGE
Source: PLoS One. 2010 Feb 11;5(2):e9162. doi: 10.1371/journal.pone.0009162 (PMC2820080; doi:10.1371/journal.pone.0009162)
Supplement: Table S3 — Input for GATHER analysis of gene ontology for genes showing similar differential expression in CIS and PC. (0.39 MB DOC) [file pone.0009162.s003.doc]

**Table S3. Input for GATHER analysis of gene ontology for genes showing similar differential expression in CIS and PC.**

| **Up-regulated: CIS_PC over BE^1^** | **Down-regulated: BE over CIS_PC^2^** |
| --- | --- |
| ADM | ABCA13 |
| APOBEC3A | ADCK4 |
| ARL7 (ARL4C) | ADRA2A |
| ARL10C (ARL8B) | ADSSL1 |
| ATP11B | FLJ12484 (AEN) |
| ADMP (C3orf57) | FLJ23598 (AGBL2) |
| CAV1 | HAG3 (AGR3) |
| CCR2 | AHSA1 |
| CD109 | AK1 |
| CD44 | AK7 |
| CDKN3 | AKAP14 |
| CNFN | AKAP2 |
| COL1A1 | AKAP9 |
| COL1A2 | AKR7A2 |
| COL3A1 | ALDH1L1 |
| COL6A1 | ALDH3B1 |
| COL7A1 | AMY2B |
| NICE-1 (CRCT1) | ANKMY1 |
| CSTA | ANKRD12 |
| CXCL14 | Lrp2bp (ANKRD37) |
| DEFB103A | AP2A2 |
| DEFB4 | APBB1 |
| DSC2 | APOBEC3G |
| DSG1 | MGC4825 (APOO) |
| DSG3 | APPL2 |
| DSP | ARHGAP18 |
| DST | ARL3 |
| EGFL7 | ARMC3 |
| EHD2 | ARMC4 |
| EMP1 | ASB3 |
| ENG | ATF7IP2 |
| ENO1 | ATPIF1 |
| EPHA1 | OAZI (AZIN1) |
| EVI2B | EPPB9 (B9D1) |
| EVPL | MGC4093 (B9D2) |
| FABP5 | BAHD1 |
| FGFBP1 | BAIAP3 |
| FOSL2 | BBS5 |
| C14ORF31 (FRMD6) | BCAS1 |
| FSCN1 | VMD2L2 (BEST4) |
| FURIN | BRD3 |
| FYN | DKFZp564B167 (BRP44) |
| GJA1 | BTBD3 |
| GNG11 | C10orf107 |
| GPNMB | C10orf63 |
| HCG9 | C10orf79 |
| HIF1A | C10orf81 |
| HMGA1 | C11orf16 |
| HMOX1 | MGC4707 (C11orf49) |
| HSPB1 | FLJ25219 (C11orf52) |
| TLH29 (IFI27L2) | FLJ21827 (C11orf60) |
| IGFBP4 | MGC13040 (C11orf70) |
| IGHG1 | FLJ46266 (C11orf88) |
| IGL@ | FLJ40919 (C13orf30) |
| C9ORF12 (IPPK) | C14orf142 |
| ITGA6 | MGC16028 (C14orf179) |
| IVL | C14orf45 |
| JUP | C14orf50 |
| KLK10 | C15orf26 |
| KLK6 | C16orf46 |
| KPNA2 | C16orf48 |
| KRT14 | MGC45438 (C16orf89) |
| KRT17 | FLJ11724 (C17orf72) |
| KRT5 | NOR1 (C1orf102) |
| KRT6A | MGC48998 (C1orf110) |
| KRT6B | DKFZp547I048 (C1orf173) |
| KRT6C | MGC34837 (C1orf87) |
| LAD1 | FLJ32884 (C1orf92) |
| LEPRE1 | C20orf114 |
| LGALS1 | C20orf26 |
| GAL7 (LGALS7B) | C20orf85 |
| MGC10986 (LIMD2) | C20orf96 |
| LMNA | C21orf59 |
| LRRC8 (LRRC8A) | C21orf63 |
| LTBP2 | C22orf15 |
| LUM | C2orf30 |
| MAD2L1 | ECRG4 (C2orf40) |
| MAFB | C3orf15 |
| MAL2 | C5orf15 |
| MALL | C6orf118 |
| FLJ10350 (MAP7D1) | C6orf97 |
| MCL1 | FLJ25903 (C7orf41) |
| MFAP1 | FLJ21062 (C7orf63) |
| MMP1 | C9orf116 |
| MMP2 | C9orf117 |
| MSN | C9orf24 |
| MYH9 | C9orf72 |
| PBEF (NAMPT) | C9orf9 |
| NCF1 | C9orf98 |
| TncRNA (NCRNA00084) | CALM1 |
| MGC4677 (NCRNA00152) | CALM2 |
| NFKB2 | CALML4 |
| PCDH1 | CAPN13 |
| PI3 | CAPS |
| PKP1 | CAPS2 |
| PKP3 | MGC26610 (CAPSL) |
| PLCB3 | CARS |
| PPM1F | CASC1 |
| PPP1R14B | H63 (CASC4) |
| PPP2R1B | CAT |
| PRNP | CBY (CBY1) |
| PRR5 | KIAA1345 (CC2D2A) |
| DKFZp434G1415 (PUS7L) | MGC15407 (CCDC104) |
| RBP1 | MGC35338 (CCDC108) |
| RNASE1 | HSPC065 (CCDC113) |
| RPS6KA4 | DKFZp434I099 (CCDC135) |
| S100A14 | C10orf80 (CCDC147) |
| S100A16 | FLJ33084 (CCDC17) |
| S100A2 | NESG1 (CCDC19) |
| S100A7 | DKFZp434A128 (CCDC39) |
| S100A8 | NYD-SP28 (CCDC65) |
| S100A9 | DKFZp434C171 (CCDC69) |
| UNQ698 (SBSN) | JFP10 (CCDC78) |
| SDC1 | CCL15 |
| SELL | UNG2 (CCNO) |
| SERPINB13 | CD59 |
| SERPINB5 | CDC42EP4 |
| SERPINE1 | PRO34300 (CDH29) |
| SFN | CDK2 |
| SGK1 | CDS1 |
| SLC16A7 | CEACAM6 |
| SLC25A5 | CELSR1 |
| SLC38A2 | FLJ37464 (CES8) |
| SLC3A2 | CETN2 |
| SMAP1 | CHST9 |
| SMC4L1 (SMC4) | CITED2 |
| SPARC | CKB |
| SPRR1A | CLIC6 |
| SPRR1B | CLMN |
| SPRR2A | CLU |
| SPRR3 | CLUAP1 |
| SRL | CNKSR1 |
| STAB1 | COL21A1 |
| TACC1 | COQ3 |
| TACC3 | COQ4 |
| TGFBI | CP |
| TIMP2 | CPD |
| TM4SF1 | CPSF2 |
| MGC5576 (TMEM106C) | CRY2 |
| HSPA5BP1 (TMEM132A) | FLJ22490 (CSPP1) |
| FLJ20173 (TMEM164) | CTGF |
| TNRC6A | CTSH |
| TP63 | CTSW |
| TUBA6 (TUBA1C) | VCC1 (CXCL17) |
| TUBA1 (TUBA4A) | CXCL6 |
| TUFT1 | CYB561 |
| TYMS | CYB5 (CYB5A) |
| UPK1B | CYB5R1 |
| VWF | NCB5OR (CYB5R4) |
| YWHAG | CYP1B1 |
| YWHAZ | CYP2B7P1 |
|  | CYP4B1 |
|  | CYP4X1 |
|  | DDAH1 |
|  | DDOST |
|  | DEFB124 |
|  | DEGS2 |
|  | DHRS3 |
|  | DIO2 |
|  | DIRAS2 |
|  | DLEC1 |
|  | EGFL9 (DLK2) |
|  | DNAH2 |
|  | DNAH5 |
|  | DNAH9 |
|  | DNAI1 |
|  | DNAI2 |
|  | DNAJA4 |
|  | TSARG5 (DNAJB13) |
|  | DNAJB2 |
|  | DNALI1 |
|  | DOC2A |
|  | DTX3 |
|  | DYDC2 |
|  | DHC2 (DYNC2H1) |
|  | DLC8 (DYNLL1) |
|  | DNCL2B (DYNLRB2) |
|  | TCTEL1 (DYNLT1) |
|  | DZIP1L |
|  | DZIP3 |
|  | FLJ11767 (EFCAB1) |
|  | MGC12458 (EFCAB2) |
|  | KIAA1799 (EFCAB7) |
|  | EFEMP1 |
|  | EFHC1 |
|  | ENPP5 |
|  | EPB41L4B |
|  | EPHX1 |
|  | SPFH1 (ERLIN1) |
|  | CVL (EZR) |
|  | F11R |
|  | FABP4 |
|  | MGC33692 (FAM116B) |
|  | DKFZp666G057 (FAM154B) |
|  | CGI-62 (FAM164A) |
|  | FLJ23093 (FAM164C) |
|  | HSD46 (FAM166A) |
|  | KIAA0423 (FAM179B) |
|  | PL48 (FAM65B) |
|  | FLJ25333 (FAM81B) |
|  | FLJ44299 (FAM92B) |
|  | FARP1 |
|  | FASTK |
|  | FBXO15 |
|  | FBXW9 |
|  | FDXR |
|  | KIAA1937 (FHAD1) |
|  | FLJ22167 |
|  | FLJ43663 |
|  | FOLR1 |
|  | FOXJ1 |
|  | FTO |
|  | FLJ22688 (FUZ) |
|  | GALC |
|  | GAS2L2 |
|  | GBP1 |
|  | GFM2 |
|  | GLB1L |
|  | GLT8D1 |
|  | GMPR2 |
|  | GP73 (GOLM1) |
|  | GOLSYN |
|  | FLJ23091 (GPR177) |
|  | GSTA2 |
|  | HAGH |
|  | HAGHL |
|  | HBB |
|  | HLA-B |
|  | HMGCL |
|  | HMGN3 |
|  | HOXA2 |
|  | HRASLS2 |
|  | HS3ST6 |
|  | HSPN (HSP90AA1) |
|  | HSPBP1 |
|  | HSPH1 |
|  | ALS2CR14 (ICA1L) |
|  | IDS |
|  | IFIT3 |
|  | SLB (IFT172) |
|  | NGD5 (IFT52) |
|  | HIPPI (IFT57) |
|  | CDV1 (IFT81) |
|  | TG737 (IFT88) |
|  | IGFBP5 |
|  | IGFBP7 |
|  | IK |
|  | IL1F10 |
|  | DVS27 (IL33) |
|  | FLJ10569 (INTS10) |
|  | IQCA (IQCA1) |
|  | IQCD |
|  | IRX3 |
|  | KAL1 |
|  | KCNE1 |
|  | KCTD12 |
|  | KIAA0746 |
|  | KIAA1377 |
|  | KIAA1529 |
|  | KIAA1688 |
|  | KIAA1797 |
|  | KIF3A |
|  | KIF3B |
|  | KIF9 |
|  | LILRB4 |
|  | WINS1 (LINS1) |
|  | FLJ35473 (LOC100132288) |
|  | LOC339047 |
|  | LOC387885 |
|  | LOC390205 |
|  | LOC400891 |
|  | LOC440335 |
|  | LRIG1 |
|  | LRP11 |
|  | LRRC16 (LRRC16A) |
|  | LRRC18 |
|  | LRRC26 |
|  | FLJ23553 (LRRC46) |
|  | DKFZp586M1120 (LRRC48) |
|  | DKFZp434K1815 (LRWD1) |
|  | LXN |
|  | LYVE1 |
|  | MAP1A |
|  | MAP6 |
|  | MAPK1 |
|  | ERK8 (MAPK15) |
|  | MAPRE3 |
|  | MDH1B |
|  | MED25 |
|  | METRN |
|  | MIPEP |
|  | MLF1 |
|  | MOPT (MORN2) |
|  | C9orf18 (MORN5) |
|  | MRPS31 |
|  | MS4A8B |
|  | MSMB |
|  | MUC5AC |
|  | MZF1 |
|  | N4BP2 |
|  | NAPSA |
|  | NBEA |
|  | FLJ36032 (NCRNA00166) |
|  | MGC10527 (NDUFAF3) |
|  | NECAB1 |
|  | NEIL1 |
|  | NFX1 |
|  | CARD7 (NLRP1) |
|  | NME5 |
|  | NPHP1 |
|  | RFRP (NPVF) |
|  | NR5A1 |
|  | NUCB2 |
|  | NUDT4 |
|  | FLJ22709 (OCEL1) |
|  | OR7E47P |
|  | OXTR |
|  | P2RY6 |
|  | PH-4 (P4HTM) |
|  | PAPD4 |
|  | PARG |
|  | PCDH9 |
|  | PCSK1N |
|  | PCSK5 |
|  | PCYT2 |
|  | PDC |
|  | PDE4A |
|  | PDGFRA |
|  | PDZRN3 |
|  | PECI |
|  | PIGR |
|  | PITPNM1 |
|  | PKIB |
|  | PKIG |
|  | HRASLS3 (PLA2G16) |
|  | PNMA1 |
|  | POLR2I |
|  | PRDX5 |
|  | PROM1 |
|  | PRSS23 |
|  | PSCA |
|  | PSENEN |
|  | PTPRN2 |
|  | PYGB |
|  | RAB36 |
|  | RABL4 |
|  | RABL5 |
|  | RAGE |
|  | RBBP4 |
|  | RBM24 |
|  | REC8 |
|  | DKFZP434i092 (RGS22) |
|  | RIF1 |
|  | RLIM |
|  | C9orf76 (RMI1) |
|  | FLJ35757 (RNF190) |
|  | ROPN1L |
|  | RP1 |
|  | DPCD (RP11-529I10.4) |
|  | RPS27L |
|  | RRAD |
|  | TSGA2 (RSPH1) |
|  | RSHL3 (RSPH4A) |
|  | C6orf206 (RSPH9) |
|  | RTDR1 |
|  | RUVBL1 |
|  | RUVBL2 |
|  | SCGB1A1 |
|  | SCGB3A1 |
|  | SPC21 (SEC11C) |
|  | SELENBP1 |
|  | SFRS5 |
|  | SFTPA2B |
|  | SFTPB |
|  | SFTPC |
|  | SLC22A4 |
|  | SLC25A4 |
|  | SLC34A2 |
|  | CTL4 (SLC44A4) |
|  | SLC9A3R2 |
|  | FLJ31952 (SLFN13) |
|  | SLPI |
|  | SMPD2 |
|  | SNAPC3 |
|  | SNRPN |
|  | SNTB1 |
|  | SOD1 |
|  | SOX30 |
|  | SPA17 |
|  | SPAG1 |
|  | SPAG16 |
|  | SPAG6 |
|  | SPATA4 |
|  | SPATS1 |
|  | SPCS1 |
|  | SPDEF |
|  | SPY1 (SPDYA) |
|  | C20orf28 (SPEF1) |
|  | FLJ23164 (SPEF2) |
|  | SPTA1 |
|  | SRI |
|  | SSBP4 |
|  | SSR3 |
|  | ST6GAL1 |
|  | GENEX3414 (STBD1) |
|  | STMP3 (STEAP3) |
|  | STK33 |
|  | C10orf24 (STOX1) |
|  | SYNE1 |
|  | SYNGAP1 |
|  | TAX1BP1 |
|  | TBC1D8 |
|  | TCEAL8 |
|  | FLJ21127 (TCTN1) |
|  | FLJ12975 (TCTN2) |
|  | TEKT1 |
|  | TEKT2 |
|  | TFF3 |
|  | TGFA |
|  | TGM2 |
|  | FLJ10916 (THNSL2) |
|  | TEGT (TMBIM6) |
|  | TMEM14B |
|  | MDAC1 (TMEM190) |
|  | TMEM45B |
|  | C1orf8 (TMEM59) |
|  | XTP3 (TMEM66) |
|  | MGC26979 (TMEM67) |
|  | PRO180 (TMEM77) |
|  | TMF1 |
|  | TMPRSS3 |
|  | MGC17791 (TNFAIP8L1) |
|  | CGI-38 (TPPP3) |
|  | TRAF3IP1 |
|  | TRIP13 |
|  | TSHR |
|  | TSNAXIP1 |
|  | TSPAN1 |
|  | TM4-A (TSPAN3) |
|  | CO-029 (TSPAN8) |
|  | TSPYL4 |
|  | TTC18 |
|  | DKFZp434H0115 (TTC25) |
|  | NYD-SP14 (TTC29) |
|  | FLJ36119 (TTLL10) |
|  | C20orf125 (TTLL9) |
|  | TUBA3 (TUBA1A) |
|  | TUBA4 (TUBA4B) |
|  | TUBB2 (TUBB2C) |
|  | TUSC3 |
|  | UBA52 |
|  | UBD |
|  | UBXD3 (UBXN10) |
|  | SOC (UBXN11) |
|  | UCP2 |
|  | UFC1 |
|  | UNC119B |
|  | VPS13B |
|  | C20orf102 (VSTM2L) |
|  | FLJ40941 (VWA3A) |
|  | WDR19 |
|  | WDR34 |
|  | WDR49 |
|  | WDR52 |
|  | WDR54 |
|  | FLJ10300 (WDR60) |
|  | FLJ30067 (WDR63) |
|  | FLJ25955 (WDR69) |
|  | FLJ10233 (WDR70) |
|  | FLJ23129 (WDR78) |
|  | C16orf15 (WDR90) |
|  | WFDC2 |
|  | WRB |
|  | XIST |
|  | XRN2 |
|  | FLJ23049 (ZBBX) |
|  | ZC3HDC8 (ZC3H8) |
|  | ZDHHC1 |
|  | ZMYND10 |
|  | ZNF214 |
|  | ZNF451 |
|  | ZNF569 |
|  | ZSCAN10 |
|  | ZNF447 (ZSCAN18) |

^1^Gene symbol; 151 genes analyzed; ^2^Gene symbol; 484 genes analyzed.

Gene symbols enclosed in brackets as cited by SAGE Genie tag-to-gene mapping, but not recognized by GATHER.
