# Supplementary material for: Transcriptome Profiles of Carcinoma-in-Situ and Invasive Non-Small Cell Lung Cancer as Revealed by SAGE
Source: PLoS One. 2010 Feb 11;5(2):e9162. doi: 10.1371/journal.pone.0009162 (PMC2820080; doi:10.1371/journal.pone.0009162)
Supplement: Table S4 — Down-regulated gene expression changes in common between carcinoma-in-situ and precancerous lesions relative to bronchial epithelium. (0.97 MB DOC) [file pone.0009162.s004.doc]

**Table S4. Down-regulated gene expression changes in common between carcinoma-in-situ and precancerous lesions relative to bronchial epithelium.**

| **Tag^1^** | **BE Mean^2^** | **CIS Mean^3^** | **SCC Mean^4^** | **PC Av^5^** | **Gene Symbol^6^** |
| --- | --- | --- | --- | --- | --- |
| AATGTGTTTA | 269 | 66 | 38 | 35 | ABCA13 |
| TCGTTATGCA | 42 | 14 | 13 | 14 | ADCK4 |
| TTGCTAATGA | 41 | 4 | 6 | 0 | ADRA2A |
| GTTGCGTGTC | 51 | 6 | 8 | 2 | ADSSL1 |
| CCTGAAAACT | 72 | 5 | 13 | 0 | AEN |
| TATTACCCAG | 71 | 3 | 4 | 2 | AGBL2 |
| AAGAAAACCT | 511 | 19 | 33 | 22 | AGR3 |
| TTTTCTGCTG | 226 | 68 | 109 | 63 | AHSA1 |
| GACAGCTGAG | 217 | 33 | 59 | 44 | AK1 |
| ACTTGTTATC | 64 | 4 | 2 | 0 | AK7 |
| GAGTCATTCC | 108 | 3 | 6 | 0 | AKAP14 |
| AGAAAGAAAA | 145 | 27 | 23 | 35 | AKAP2 |
| TTTTGCCTAA | 69 | 13 | 14 | 19 | AKAP9 |
| CATTATTTTT | 58 | 13 | 14 | 2 | AKR7A2 |
| TCTGCATCTT | 106 | 9 | 23 | 3 | ALDH1L1 |
| CTGCTCATCC | 188 | 0 | 11 | 12 | ALDH3B1 |
| CTGAATCTAA | 327 | 12 | 18 | 37 | AMY2B |
| CTTTGTAGCA | 83 | 6 | 7 | 18 | ANKMY1 |
| ACAAAGAAAA | 65 | 19 | 12 | 0 | ANKRD12 |
| CCTATAATAT | 81 | 23 | 22 | 5 | ANKRD37 |
| GTTGCATCCC | 65 | 2 | 6 | 8 | AP2A2 |
| GGCACCTCTG | 86 | 14 | 17 | 12 | APBB1 |
| CAGAGCTGTG | 58 | 3 | 11 | 0 | APOBEC3G |
| TTGCTATTTA | 157 | 46 | 54 | 25 | APOO |
| GGGCTTGGTA | 94 | 27 | 20 | 16 | APPL2 |
| ATACTTTTAG | 42 | 0 | 4 | 6 | ARHGAP18 |
| AACTGGGTCT | 52 | 4 | 12 | 6 | ARL3 |
| TTCCATCCAG | 76 | 1 | 8 | 0 | ARMC3 |
| TGATAAGATG | 104 | 4 | 12 | 0 | ARMC4 |
| CAATTAAAGC | 61 | 1 | 3 | 0 | ASB3 |
| ATCCCTGCAC | 47 | 4 | 8 | 10 | ATF7IP2 |
| GTTCTGGTTT | 632 | 137 | 197 | 134 | ATPIF1 |
| TGAATGATAC | 97 | 24 | 23 | 10 | AZIN1 |
| AGGAAACTGG | 74 | 7 | 16 | 9 | B9D1 |
| GGAGGTGCTC | 138 | 16 | 23 | 25 | B9D2 |
| TAAAATATAT | 48 | 16 | 25 | 14 | BAHD1 |
| TCTCTAGATT | 185 | 8 | 17 | 17 | BAIAP3 |
| TTCGGTTTAA | 82 | 12 | 17 | 5 | BBS5 |
| TAAACTGTAT | 91 | 9 | 8 | 2 | BCAS1 |
| TGAAACGTGC | 46 | 1 | 6 | 6 | BEST4 |
| AAGGATTCAC | 59 | 5 | 17 | 0 | BRD3 |
| ACTTAAGGAA | 113 | 38 | 97 | 26 | BRP44 |
| TTAAATGCAA | 133 | 45 | 44 | 39 | BTBD3 |
| GATCCTTTAG | 48 | 0 | 2 | 6 | C10orf107 |
| AACATTTCAA | 51 | 2 | 1 | 3 | C10orf63 |
| AATAAATGTG | 241 | 4 | 17 | 7 | C10orf79 |
| AATTGCTGTT | 126 | 1 | 6 | 2 | C10orf79 |
| TGGGTATTGC | 89 | 8 | 9 | 10 | C10orf81 |
| AGTGACATTT | 188 | 6 | 9 | 0 | C11orf16 |
| TCTGGTAAAA | 77 | 19 | 18 | 18 | C11orf49 |
| AGGTTAGGTG | 40 | 1 | 6 | 0 | C11orf52 |
| GAGGTTAGAT | 88 | 7 | 25 | 15 | C11orf60 |
| TCTTAATGTT | 66 | 4 | 4 | 2 | C11orf70 |
| GCAGAGGATG | 365 | 8 | 18 | 2 | C11orf88 |
| TGCAACTACA | 52 | 8 | 5 | 0 | C12orf75 |
| ATTGTAAAGA | 84 | 2 | 1 | 0 | C13orf30 |
| TACCTTAAGA | 107 | 11 | 16 | 11 | C14orf142 |
| AAGAAATGCA | 84 | 11 | 17 | 8 | C14orf179 |
| TTAGCTGAGA | 258 | 9 | 27 | 7 | C14orf45 |
| CCCTAAGTAA | 42 | 0 | 0 | 2 | C14orf50 |
| TGATTAGATA | 63 | 3 | 4 | 0 | C15orf26 |
| GCTGTGGTCT | 47 | 5 | 8 | 8 | C16orf46 |
| GCTGCAAAGG | 101 | 21 | 16 | 24 | C16orf48 |
| CCAGCTGCCT | 60 | 1 | 5 | 0 | C16orf89 |
| ATGGTTTCCG | 174 | 2 | 7 | 2 | C17orf72 |
| CATCCAGCAG | 47 | 0 | 6 | 5 | C17orf72 |
| GCCTGCCTTT | 85 | 6 | 7 | 2 | C17orf97 |
| GCCCAGAATG | 70 | 1 | 6 | 5 | C1orf102 |
| GTCTATAAAG | 70 | 14 | 5 | 6 | C1orf110 |
| CAGTTAAGTG | 64 | 5 | 15 | 0 | C1orf133 |
| TGAATTTAAC | 108 | 1 | 5 | 0 | C1orf173 |
| CCTGCTAACC | 44 | 0 | 1 | 0 | C1orf189 |
| ATCCAGACAG | 86 | 1 | 9 | 0 | C1orf192 |
| CCTCCCACCC | 61 | 2 | 3 | 2 | C1orf194 |
| TTTCTCCCCA | 44 | 1 | 1 | 2 | C1orf87 |
| TATTGGAGCT | 92 | 1 | 5 | 2 | C1orf88 |
| TGCAGATTTT | 60 | 1 | 4 | 0 | C1orf88 |
| GCATTCTTCC | 74 | 0 | 3 | 2 | C1orf92 |
| CCAAGGTGGC | 2567 | 78 | 297 | 53 | C20orf114 |
| GTCCTGTTGG | 66 | 2 | 5 | 0 | C20orf114 |
| TTCCTGTGCA | 74 | 1 | 3 | 0 | C20orf26 |
| GGATGTTGCA | 493 | 8 | 29 | 0 | C20orf85 |
| GGCCGCCCTC | 58 | 1 | 6 | 0 | C20orf96 |
| GAGTGAAAGA | 153 | 26 | 23 | 11 | C21orf59 |
| TCATTCAACA | 57 | 9 | 23 | 19 | C21orf63 |
| AGCAAAGCCC | 57 | 6 | 4 | 0 | C22orf15 |
| GGAGTTGTCC | 44 | 14 | 17 | 12 | C2orf30 |
| GGAATGCCTC | 934 | 20 | 42 | 16 | C2orf40 |
| TGGAACCGGA | 47 | 1 | 7 | 0 | C2orf81 |
| TGTAATGAAA | 109 | 4 | 2 | 3 | C3orf15 |
| AGGAATGGTA | 47 | 16 | 11 | 13 | C4orf3 |
| GTTGACTTAC | 50 | 8 | 19 | 6 | C5orf15 |
| GAATGATTTC | 418 | 52 | 118 | 110 | C5orf32 |
| ATTAGTTTCT | 62 | 1 | 2 | 0 | C6orf118 |
| TATACAGTCC | 177 | 3 | 11 | 2 | C6orf97 |
| TAATTACCAT | 66 | 16 | 5 | 18 | C7orf41 |
| CATTTGCTTA | 114 | 4 | 4 | 0 | C7orf57 |
| AGCCAAATGG | 45 | 1 | 6 | 5 | C7orf63 |
| AACAGCTTTA | 68 | 12 | 29 | 6 | C8orf40 |
| TTAAAATTTA | 41 | 12 | 6 | 0 | C8orf83 |
| GGAAAGATGC | 586 | 34 | 48 | 25 | C9orf116 |
| CTTCTGAGGG | 183 | 0 | 12 | 0 | C9orf117 |
| GCCCACCCAA | 50 | 0 | 3 | 0 | C9orf117 |
| ATGAGAGTGG | 135 | 0 | 14 | 0 | C9orf135 |
| GTTACGAAAG | 345 | 3 | 22 | 0 | C9orf24 |
| TGTAAACATT | 106 | 27 | 20 | 30 | C9orf72 |
| ACAGAAGCCA | 85 | 5 | 11 | 0 | C9orf9 |
| GAGATCCAGG | 42 | 5 | 11 | 10 | C9orf98 |
| ACAAACTTAG | 252 | 66 | 68 | 50 | CALM1 |
| ACTTGGAGCC | 230 | 72 | 63 | 58 | CALM1 |
| GGTGGAGTGT | 46 | 10 | 10 | 3 | CALM1 |
| TTGTTGTTGA | 600 | 124 | 182 | 162 | CALM2 |
| GTATGAGTAG | 309 | 21 | 50 | 24 | CALML4 |
| TTTCCCATAA | 52 | 3 | 11 | 2 | CAPN13 |
| CTGACCAGAG | 1850 | 23 | 96 | 44 | CAPS |
| CAGAGGCCAG | 258 | 5 | 26 | 2 | CAPS |
| AGAAGAGGCT | 53 | 0 | 6 | 3 | CAPS2 |
| TGGCTCCAAA | 195 | 5 | 7 | 2 | CAPSL |
| TAGGTTCGTG | 270 | 76 | 75 | 58 | CARS |
| GTGAAAGACA | 87 | 1 | 8 | 0 | CASC1 |
| CAATTACCTG | 60 | 8 | 20 | 0 | CASC4 |
| GCTTAATGTT | 135 | 27 | 37 | 27 | CAT |
| GATGAGCGGC | 63 | 12 | 15 | 6 | CBY1 |
| TAAAAACTAC | 73 | 8 | 6 | 6 | CC2D2A |
| AGAAAGGATA | 55 | 12 | 5 | 2 | CCDC104 |
| CCGCTAGGGG | 124 | 5 | 3 | 3 | CCDC108 |
| TTTTGCAATA | 238 | 7 | 11 | 11 | CCDC113 |
| ATTTTCCTGT | 109 | 2 | 4 | 0 | CCDC135 |
| AGATGAGAAA | 43 | 1 | 11 | 0 | CCDC147 |
| GAGGCGAAGT | 66 | 2 | 1 | 2 | CCDC153 |
| TAGCCTGTGG | 41 | 3 | 4 | 0 | CCDC157 |
| TTCTGACATT | 363 | 7 | 18 | 2 | CCDC17 |
| CCAATGAGCT | 51 | 3 | 11 | 0 | CCDC19 |
| TGCCCAACAC | 63 | 2 | 3 | 5 | CCDC39 |
| GAGTTTAAAA | 70 | 6 | 2 | 0 | CCDC65 |
| CGGCACCTTA | 139 | 43 | 31 | 30 | CCDC69 |
| AGTCAGGATA | 994 | 33 | 62 | 25 | CCDC78 |
| AAAAAGCTGA | 57 | 0 | 6 | 0 | CCL15 |
| AGGAAGCTGA | 277 | 20 | 63 | 25 | CCNO |
| AGCTGTCTCA | 150 | 49 | 44 | 50 | CD59 |
| TGACTGGCAG | 147 | 34 | 33 | 11 | CD59 |
| GCGACTGTGG | 115 | 7 | 5 | 0 | CDC42EP4 |
| GGCTGTATTT | 342 | 3 | 22 | 2 | CDH29 |
| GGTAAAAATA | 40 | 4 | 17 | 5 | CDK2 |
| TAGTCTGGAG | 97 | 26 | 15 | 11 | CDS1 |
| AAGGATAAAA | 209 | 14 | 37 | 56 | CEACAM6 |
| ACTTTAACAG | 60 | 11 | 15 | 6 | CELSR1 |
| GAGCAAACTT | 53 | 5 | 4 | 2 | CES8 |
| TTACACCTGT | 477 | 25 | 41 | 15 | CETN2 |
| CTAGGATGAT | 71 | 13 | 7 | 13 | CHP2 |
| AATATATTTT | 97 | 13 | 8 | 11 | CHST9 |
| AAAAGATACT | 65 | 15 | 32 | 9 | CITED2 |
| CACCCCTGAT | 292 | 79 | 53 | 87 | CKB |
| ATAAATGATT | 236 | 8 | 29 | 58 | CLIC6 |
| TGTATTCTTA | 142 | 4 | 21 | 47 | CLMN |
| CAACTAATTC | 1134 | 229 | 401 | 107 | CLU |
| TTCACAATAC | 66 | 6 | 6 | 3 | CLUAP1 |
| AGGGCTACTT | 70 | 11 | 25 | 10 | CNKSR1 |
| CTTGTTATTC | 57 | 5 | 9 | 6 | COL21A1 |
| TAAAACTCTA | 46 | 1 | 2 | 0 | COL28A1 |
| AAGAAAAGAA | 94 | 23 | 18 | 12 | COQ3 |
| CTGGGATGTC | 133 | 34 | 35 | 45 | COQ4 |
| TCTCCAACAA | 165 | 26 | 86 | 37 | CP |
| CACTGTGCCT | 40 | 8 | 20 | 3 | CPD |
| CTCCAAAAAA | 58 | 13 | 15 | 7 | CPSF2 |
| GGGAAGAGAT | 54 | 11 | 10 | 17 | CRY2 |
| ATGTAACTAC | 120 | 11 | 16 | 6 | CSPP1 |
| TTTGCACCTT | 531 | 144 | 440 | 117 | CTGF |
| GACCACGAAT | 138 | 36 | 99 | 39 | CTSH |
| GCAGCGGCAG | 1421 | 6 | 6 | 81 | CTSW |
| TTTTTCAAGA | 501 | 72 | 99 | 106 | CXCL17 |
| ACAGTGTCTG | 59 | 2 | 12 | 3 | CXCL17 |
| TTGGTTTTTG | 362 | 115 | 180 | 102 | CXCL6 |
| TGGGAAAACT | 79 | 21 | 29 | 0 | CYB561 |
| ATCTTTTAAA | 477 | 72 | 154 | 95 | CYB5A |
| TACGCTTGGT | 40 | 13 | 9 | 7 | CYB5R1 |
| ATCTCAACTT | 61 | 15 | 12 | 6 | CYB5R4 |
| AATGCTTTTA | 55 | 2 | 9 | 16 | CYP1B1 |
| ACCTCCCCAC | 52 | 3 | 6 | 10 | CYP2B7P1 |
| GTTATGGCTG | 563 | 15 | 53 | 35 | CYP4B1 |
| CCCTGACCAA | 48 | 12 | 5 | 3 | CYP4X1 |
| TATACCAATC | 111 | 14 | 21 | 34 | DDAH1 |
| AAGGAGAAGG | 47 | 16 | 59 | 13 | DDOST |
| AATATGAGTA | 89 | 2 | 2 | 2 | DEFB124 |
| ACCTGGGTGC | 152 | 7 | 10 | 31 | DEGS2 |
| CTTCTGCTGG | 47 | 4 | 44 | 12 | DHRS3 |
| CTTTGAGACC | 44 | 1 | 0 | 3 | DIO2 |
| TATATAAAAT | 58 | 1 | 4 | 10 | DIRAS2 |
| ATGAAACGCT | 53 | 3 | 6 | 2 | DKFZP434B2016 |
| CACTTTTACT | 128 | 3 | 6 | 7 | DLEC1 |
| CTGCCAGCAG | 45 | 5 | 8 | 5 | DLK2 |
| AGCCTGGACA | 69 | 1 | 7 | 0 | DNAH2 |
| AGAGTTAGGA | 140 | 0 | 9 | 0 | DNAH5 |
| GTCTAAGCTT | 89 | 1 | 6 | 0 | DNAH9 |
| AGCACTCACA | 78 | 1 | 5 | 0 | DNAH9 |
| TGACCCCACT | 70 | 0 | 5 | 0 | DNAI1 |
| ATCGACCCTC | 83 | 2 | 4 | 9 | DNAI2 |
| CAGGTAAGGT | 162 | 7 | 16 | 19 | DNAJA4 |
| ACTGTGGTGG | 75 | 3 | 6 | 2 | DNAJB13 |
| CCGCTGCTTG | 59 | 19 | 17 | 14 | DNAJB2 |
| ACTTTATGAC | 70 | 2 | 8 | 8 | DNAL1 |
| ATTAATTTCC | 96 | 2 | 4 | 6 | DNALI1 |
| GCAGCTGGGC | 86 | 5 | 5 | 2 | DOC2A |
| ATCATTCCCT | 100 | 27 | 32 | 2 | DPY30 |
| GAGCCGGCTG | 49 | 11 | 12 | 0 | DTX3 |
| TGAACATTTG | 169 | 9 | 8 | 0 | DYDC2 |
| TGGTCAGTCT | 49 | 1 | 6 | 0 | DYNC2H1 |
| GACTGTGCCA | 462 | 116 | 146 | 65 | DYNLL1 |
| AGTATTAAAA | 335 | 3 | 18 | 5 | DYNLRB2 |
| AATTCAGTGA | 126 | 18 | 32 | 41 | DYNLT1 |
| GGTGGGAAGG | 54 | 7 | 9 | 0 | DZIP1L |
| TCTCTGGTTT | 142 | 15 | 18 | 18 | DZIP3 |
| TCTTCCTGAT | 50 | 0 | 4 | 0 | EFCAB1 |
| TAAAGATCTT | 48 | 2 | 5 | 0 | EFCAB2 |
| GTATGTACTT | 44 | 10 | 6 | 0 | EFCAB7 |
| CAAGGGTAAG | 118 | 38 | 79 | 37 | EFEMP1 |
| GAGAAGGCAA | 153 | 7 | 11 | 5 | EFHC1 |
| AATATACTTT | 48 | 4 | 2 | 6 | ENPP5 |
| TTAGGCCCTC | 53 | 8 | 9 | 16 | EPB41L4B |
| GCTTTGATGA | 1056 | 345 | 640 | 166 | EPHX1 |
| TGTGAGGAGT | 49 | 16 | 28 | 8 | ERLIN1 |
| CTTAGAGGGG | 86 | 11 | 34 | 22 | EZR |
| GCTGGAGCGC | 51 | 12 | 29 | 16 | F11R |
| ATTTAGCAAG | 47 | 2 | 15 | 2 | FABP4 |
| CTGGGCCCCA | 41 | 1 | 1 | 6 | FAM116B |
| CAACATAATA | 431 | 5 | 29 | 2 | FAM154B |
| AAGCATAAAA | 64 | 17 | 13 | 12 | FAM164A |
| AGCAGGCTCC | 50 | 2 | 6 | 2 | FAM164C |
| GATGTGGACA | 159 | 38 | 27 | 25 | FAM166A |
| ATGCTCTGGG | 129 | 3 | 7 | 0 | FAM166B |
| GTATATTTTG | 194 | 3 | 5 | 11 | FAM179A |
| GGCTTACAAG | 53 | 8 | 13 | 6 | FAM179B |
| ACCGCAGGCT | 276 | 7 | 14 | 3 | FAM183A |
| TAAGTTAAAA | 211 | 20 | 13 | 12 | FAM65B |
| ACTCTCTCAG | 60 | 3 | 4 | 2 | FAM81B |
| GAGGAGGCCC | 205 | 4 | 18 | 0 | FAM92B |
| GCTTCCCAGC | 44 | 12 | 14 | 11 | FARP1 |
| CTTCGAGTCC | 117 | 2 | 0 | 10 | FASTK |
| GGAAACCCTT | 42 | 2 | 4 | 2 | FBXO15 |
| TTGGTGCTTG | 220 | 47 | 38 | 72 | FBXW9 |
| CTGGAAATAA | 108 | 36 | 43 | 32 | FDXR |
| TGACTCTTCT | 73 | 1 | 7 | 2 | FHAD1 |
| ATGTTTTGTA | 346 | 11 | 27 | 16 | FLJ22167 |
| GTGGTTCATT | 62 | 2 | 6 | 3 | FLJ22167 |
| TCCCTATTAG | 94 | 29 | 15 | 13 | FLJ36644 |
| TAATACTCCA | 96 | 9 | 11 | 0 | FLJ43663 |
| GTCGGGCCTC | 166 | 16 | 36 | 6 | FOLR1 |
| AAAGTTATTT | 1071 | 13 | 63 | 31 | FOXJ1 |
| TCAGTGCTCT | 45 | 9 | 6 | 3 | FTO |
| GGGCTGTTAG | 46 | 3 | 11 | 3 | FUZ |
| TTGTGTGATT | 51 | 9 | 14 | 3 | GALC |
| TGATTCTGAA | 77 | 2 | 3 | 13 | GAS2L2 |
| CAATGATGCA | 58 | 2 | 24 | 8 | GBP1 |
| CTGTTCATCT | 54 | 9 | 12 | 3 | GFM2 |
| GAGTGGGCAG | 60 | 1 | 8 | 0 | GLB1L |
| GAAAAAGATG | 40 | 8 | 13 | 6 | GLT8D1 |
| GCCTTGGTCT | 65 | 3 | 9 | 0 | GMPR2 |
| TTTTGTTAAT | 44 | 14 | 26 | 11 | GMPR2 |
| TTTAATTTGT | 192 | 22 | 44 | 64 | GOLM1 |
| ATGAAAATAA | 76 | 10 | 9 | 15 | GOLSYN |
| AGAAGATTTA | 47 | 15 | 27 | 6 | GPR177 |
| CAAGACCAGT | 659 | 2 | 10 | 3 | GSTA2 |
| CGGGCCGTGC | 45 | 11 | 17 | 0 | HAGH |
| AGGGCCACCT | 62 | 3 | 9 | 0 | HAGHL |
| GCAAGAAAGT | 407 | 34 | 161 | 15 | HBB |
| CCAGCCATCT | 107 | 2 | 8 | 2 | hCG_17324 |
| CTGACTTGTG | 55 | 0 | 82 | 0 | HLA-B |
| GAGGTTCTTC | 40 | 13 | 13 | 11 | HMGCL |
| GATAGTTGTG | 828 | 163 | 164 | 174 | HMGN3 |
| CTTTTTGGAA | 100 | 24 | 27 | 14 | HMGN3 |
| CATATTTTTT | 44 | 13 | 23 | 3 | HOXA2 |
| AATCTTTATT | 44 | 5 | 6 | 5 | HRASLS2 |
| CTGGGCAGAG | 99 | 6 | 6 | 15 | HS3ST6 |
| TACTAGTCCT | 684 | 181 | 149 | 225 | HSP90AA1 |
| CTCTCACTCT | 96 | 32 | 38 | 5 | HSPBP1 |
| GGGGACGGGA | 63 | 9 | 21 | 8 | HSPBP1 |
| TGAAAGTGTG | 411 | 85 | 93 | 44 | HSPH1 |
| TGCTCTATCT | 44 | 5 | 6 | 6 | ICA1L |
| CATCAACAAT | 43 | 8 | 13 | 6 | IDS |
| GTTTAGAGGG | 135 | 14 | 33 | 38 | IFIT3 |
| GACCAGCGGC | 68 | 6 | 5 | 2 | IFT172 |
| AGCTCTGGAA | 48 | 11 | 18 | 14 | IFT52 |
| GTGATTATGA | 311 | 23 | 35 | 23 | IFT57 |
| TCTTTTCTGT | 72 | 12 | 8 | 2 | IFT81 |
| TTAAACCTTG | 86 | 3 | 9 | 2 | IFT88 |
| GATAGCACAG | 43 | 10 | 31 | 0 | IGFBP5 |
| CATATCATTA | 859 | 288 | 1055 | 206 | IGFBP7 |
| GCTGTGGATA | 127 | 5 | 21 | 22 | IK |
| GATTTCAGCT | 45 | 5 | 4 | 13 | IL1F10 |
| TGCATCTGTG | 86 | 24 | 76 | 15 | IL33 |
| TTTTAGAGCA | 44 | 4 | 4 | 12 | IL33 |
| GCACAGAGCC | 42 | 11 | 12 | 3 | INTS10 |
| TACACTGTAT | 61 | 7 | 7 | 3 | IQCA1 |
| TTTTTATTTA | 169 | 37 | 44 | 45 | IQCD |
| TCCGTGTATA | 85 | 18 | 29 | 9 | IRX3 |
| TTAGAGTTTG | 78 | 10 | 27 | 13 | KAL1 |
| TACTGTTCTA | 67 | 0 | 2 | 0 | KCNE1 |
| CCCAAACTTT | 99 | 9 | 24 | 21 | KCTD12 |
| CCAAGTATTT | 45 | 10 | 17 | 9 | KCTD12 |
| CTGCTAAGGT | 99 | 29 | 44 | 16 | KIAA0746 |
| TAATTTACCC | 103 | 2 | 4 | 0 | KIAA1377 |
| AATAAACACT | 139 | 4 | 5 | 3 | KIAA1529 |
| GACCCTGAAA | 75 | 12 | 9 | 17 | KIAA1688 |
| CCTCCTAAGA | 43 | 6 | 21 | 6 | KIAA1797 |
| GTTAAATAAG | 71 | 9 | 8 | 12 | KIF3A |
| GCTCCTTGAA | 48 | 12 | 17 | 0 | KIF3B |
| AAATGATCAG | 52 | 1 | 4 | 6 | KIF9 |
| AGATTGAGGG | 292 | 5 | 12 | 3 | LDLRAD1 |
| AAAATCAAAT | 74 | 3 | 6 | 3 | LDLRAD1 |
| GGAAAATGGG | 76 | 12 | 15 | 20 | LILRB4 |
| CTGGAGGCTG | 135 | 9 | 21 | 25 | LINS1 |
| AGGAGCGGGG | 96 | 29 | 72 | 27 | LOC100129700 |
| AGATATTCAA | 236 | 3 | 13 | 5 | LOC100131176 |
| GCCCATCGTA | 100 | 24 | 22 | 5 | LOC100132288 |
| CACCTAATTG | 6487 | 2078 | 3561 | 1715 | LOC100133315 |
| GAAATGTTTC | 53 | 9 | 28 | 10 | LOC339047 |
| CTGGCCGGCC | 65 | 0 | 9 | 3 | LOC387885 |
| GTAATGTTTT | 348 | 9 | 16 | 2 | LOC390205 |
| ACCGCATTTA | 45 | 0 | 3 | 0 | LOC400891 |
| GGCACCGTGC | 247 | 12 | 37 | 24 | LOC440335 |
| TCCTCTAAAT | 197 | 5 | 16 | 0 | LOC645757 |
| TGCTCTGAAT | 166 | 46 | 36 | 41 | LOC727751 |
| TGATTATTAA | 85 | 1 | 2 | 0 | LOC728196 |
| ACCCTCTGTG | 71 | 15 | 18 | 12 | LOC728855 |
| TAAAATATTG | 172 | 29 | 64 | 15 | LRIG1 |
| ATTCCTTTAA | 394 | 48 | 72 | 47 | LRP11 |
| AGCTTAATGA | 1283 | 1 | 7 | 126 | LRRC16A |
| ACTCAAATAA | 53 | 0 | 6 | 0 | LRRC18 |
| TATGACAGAG | 171 | 2 | 9 | 0 | LRRC23 |
| CCTTTGCCCT | 54 | 10 | 9 | 8 | LRRC26 |
| TCACTTGGGG | 158 | 5 | 6 | 5 | LRRC46 |
| CAGTCTGATT | 132 | 1 | 5 | 0 | LRRC46 |
| CAGAGCGAAC | 102 | 1 | 7 | 0 | LRRC48 |
| ATAAACATTT | 602 | 10 | 29 | 11 | LRRC50 |
| GTTTGACAAT | 77 | 2 | 3 | 6 | LRTOMT |
| ACAGGCCAAG | 60 | 5 | 8 | 2 | LRTOMT |
| GTGAACACAG | 48 | 7 | 14 | 10 | LRWD1 |
| TCTATTGATG | 65 | 6 | 13 | 0 | LXN |
| TCTACAGAAA | 120 | 3 | 10 | 6 | LYVE1 |
| TGGGCTTGCC | 41 | 10 | 7 | 8 | MAP1A |
| TACAGTAGTC | 159 | 5 | 10 | 0 | MAP6 |
| CTTTGGGTCC | 159 | 2 | 1 | 23 | MAPK1 |
| TCTGCCCCCA | 310 | 11 | 14 | 3 | MAPK15 |
| TGTGAGCCGC | 52 | 2 | 2 | 2 | MAPK15 |
| TGAGGGATGG | 89 | 19 | 9 | 9 | MAPRE3 |
| CAAGGCAATT | 56 | 0 | 4 | 0 | MDH1B |
| TTTGTTTCAT | 51 | 1 | 2 | 0 | MDH1B |
| GATCTCATCT | 159 | 46 | 46 | 32 | MED25 |
| GGCTGGAGCC | 99 | 15 | 12 | 12 | METRN |
| TAGATAATGA | 63 | 8 | 19 | 15 | MIPEP |
| AAGGTCCTAG | 97 | 15 | 24 | 14 | MLF1 |
| TAGATGTGAT | 357 | 14 | 33 | 18 | MORN2 |
| AGAATAAAGA | 77 | 18 | 20 | 11 | MORN3 |
| AGTGGATCAC | 205 | 0 | 10 | 0 | MORN5 |
| GAAATTTTTA | 322 | 45 | 37 | 26 | MRPS31 |
| TGAGCTTGTG | 940 | 14 | 63 | 11 | MS4A8B |
| CCTATCAGTA | 7191 | 436 | 537 | 85 | MSMB |
| GTGATCAGCT | 2036 | 116 | 218 | 133 | MUC5AC |
| CTTCCTGTGA | 50 | 3 | 4 | 9 | MUCL1 |
| GTCAGAACAC | 58 | 8 | 8 | 11 | MZF1 |
| CTTTTGCCCT | 96 | 7 | 9 | 26 | N4BP2 |
| CGCAGCGGGT | 63 | 1 | 222 | 0 | NAPSA |
| TTTTATTGCA | 47 | 4 | 3 | 0 | NBEA |
| AGAGCCCTAC | 74 | 1 | 5 | 0 | NCRNA00166 |
| AAGGCCGAGT | 62 | 17 | 25 | 13 | NDUFAF3 |
| TTATTTTCTA | 141 | 1 | 15 | 6 | NECAB1 |
| TGAAAAATGC | 64 | 17 | 22 | 19 | NEIL1 |
| AGCCCCAGAG | 42 | 9 | 10 | 5 | NFX1 |
| AATGTTAAAT | 74 | 1 | 3 | 6 | NLRP1 |
| TGTCAGATAT | 107 | 2 | 8 | 0 | NME5 |
| AACTATCTTA | 71 | 1 | 2 | 3 | NME5 |
| AACCTTTTGA | 49 | 2 | 4 | 3 | NPHP1 |
| CATTCACCAT | 70 | 13 | 24 | 21 | NPVF |
| TGCCCCTGCC | 43 | 0 | 1 | 0 | NR5A1 |
| ATCAACTGGA | 199 | 17 | 29 | 11 | NUCB2 |
| AAAGGAGAGA | 54 | 13 | 13 | 8 | NUCB2 |
| GTCTCATTTG | 194 | 44 | 54 | 39 | NUDT4 |
| AGTGACAGAG | 192 | 14 | 9 | 50 | NWD1 |
| TATTAGACAC | 62 | 7 | 20 | 2 | OCEL1 |
| TATTAGACAA | 52 | 6 | 12 | 6 | OCEL1 |
| ACCGTGCGCG | 121 | 4 | 14 | 0 | ODF3B |
| TATTTCCCTA | 53 | 0 | 2 | 0 | OR7E47P |
| ACTACTAAGG | 49 | 2 | 0 | 0 | OXTR |
| CTTTGAGTCA | 211 | 1 | 1 | 19 | P2RY6 |
| TTATTTATTG | 275 | 22 | 35 | 30 | P4HTM |
| TGTTTTCAGG | 50 | 12 | 13 | 12 | PAPD4 |
| CTGATTTGTA | 80 | 16 | 24 | 6 | PARG |
| CAACAAAATG | 47 | 6 | 4 | 5 | PCDH9 |
| ATCTGAAGCA | 82 | 16 | 18 | 0 | PCSK1N |
| TACATTTCAA | 70 | 7 | 14 | 11 | PCSK5 |
| GCTGACGGAA | 52 | 9 | 17 | 8 | PCYT2 |
| AGAAAATAAA | 110 | 7 | 14 | 16 | PDC |
| GTGCACTGTG | 42 | 12 | 18 | 6 | PDE4A |
| TGATTGGTGG | 170 | 56 | 60 | 27 | PDGFRA |
| TTTGCAAATA | 523 | 10 | 32 | 7 | PDZRN3 |
| TCCAAGGAAG | 53 | 14 | 16 | 3 | PECI |
| GACCCAAGAT | 409 | 53 | 101 | 28 | PIGR |
| TGAATTAAAG | 78 | 2 | 6 | 0 | PIH1D2 |
| AAAATAAACG | 210 | 39 | 45 | 66 | PITPNM1 |
| CCTATTAAAT | 63 | 7 | 7 | 21 | PKIB |
| GAGCTCCACA | 125 | 17 | 45 | 22 | PKIG |
| TTCTCAAGAA | 40 | 6 | 17 | 6 | PLA2G16 |
| GAGAGGTTGA | 78 | 21 | 33 | 6 | PNMA1 |
| CGCCTTTACT | 59 | 17 | 26 | 20 | POLR2I |
| GTGGTACAGG | 839 | 120 | 204 | 127 | PRDX5 |
| GAGAACCTCT | 55 | 9 | 10 | 15 | PRDX5 |
| TGCAGATTGC | 351 | 49 | 55 | 13 | PROM1 |
| CCTGTCTGCA | 48 | 8 | 27 | 15 | PRSS23 |
| TATGTCTGCA | 44 | 8 | 6 | 5 | PSCA |
| TCCCACGTTC | 197 | 16 | 39 | 40 | PSENEN |
| GTATTGTAAT | 53 | 6 | 1 | 0 | PTPRN2 |
| GGGGTCAGGG | 354 | 100 | 78 | 48 | PYGB |
| GTGGACTGTC | 85 | 4 | 12 | 0 | RAB36 |
| ACGAGCTGGA | 206 | 54 | 38 | 12 | RABL4 |
| TTTCTCGGTG | 43 | 5 | 8 | 0 | RABL5 |
| TCTTACCAGT | 78 | 6 | 9 | 5 | RAGE |
| TTGAAATATA | 64 | 12 | 11 | 15 | RBBP4 |
| ACTCTCCTGT | 44 | 1 | 6 | 3 | RBM24 |
| GAGATGCCTT | 44 | 2 | 5 | 13 | REC8 |
| AGAAGCTCCA | 59 | 1 | 3 | 2 | RGS22 |
| ACAATACAAA | 84 | 2 | 6 | 2 | RIF1 |
| TCTTGATCAG | 67 | 0 | 0 | 6 | RLIM |
| GATTTGTATT | 57 | 1 | 1 | 0 | RMI1 |
| TATTCATTCA | 49 | 2 | 1 | 0 | RNF190 |
| ATAGGTCTTT | 196 | 7 | 10 | 3 | ROPN1L |
| GAACACTATT | 46 | 1 | 2 | 0 | RP1 |
| GTCATATTTC | 151 | 23 | 23 | 11 | RP11-529I10.4 |
| CTCAGACAGT | 76 | 20 | 36 | 23 | RPS27L |
| CAGATTTTTG | 328 | 52 | 60 | 27 | RRAD |
| GATGAGGGAA | 208 | 5 | 11 | 5 | RSPH1 |
| GTCCAGGTGA | 343 | 8 | 24 | 0 | RSPH4A |
| TATTCTTAAA | 50 | 1 | 3 | 0 | RSPH4A |
| ATTTTCTTAA | 600 | 7 | 24 | 22 | RSPH9 |
| GAGGTGGAGA | 46 | 1 | 4 | 0 | RTDR1 |
| AAGTGAGATG | 99 | 22 | 26 | 15 | RUVBL1 |
| GACACCTCCT | 223 | 50 | 100 | 46 | RUVBL2 |
| CTTTGAGTCC | 42246 | 98 | 366 | 2547 | SCGB1A1 |
| AGGGAGGCAG | 409 | 14 | 13 | 12 | SCGB1A1 |
| GAAAAAATAG | 251 | 0 | 2 | 10 | SCGB1A1 |
| AAGCTCGCCG | 3651 | 142 | 115 | 16 | SCGB3A1 |
| AAAAAGATAA | 104 | 17 | 8 | 2 | SEC11C |
| AGCTCTTGGA | 584 | 73 | 170 | 168 | SELENBP1 |
| AGACAAGCTG | 63 | 20 | 29 | 19 | SFRS5 |
| CTCCCAGCCA | 532 | 5 | 1009 | 0 | SFTPA2B |
| AGGACACCAA | 61 | 4 | 143 | 5 | SFTPB |
| ATGGGATGGC | 47 | 0 | 173 | 0 | SFTPB |
| GCCGTGAGCA | 288 | 50 | 118 | 28 | SFTPC |
| TTAGTCAAGG | 155 | 2 | 7 | 2 | SLC22A4 |
| TATTTTGTAT | 55 | 18 | 26 | 11 | SLC25A4 |
| CCTGCCCCGC | 51 | 1 | 35 | 0 | SLC34A2 |
| CACCTGTCAT | 417 | 15 | 46 | 60 | SLC44A4 |
| TTTCCACTTA | 102 | 15 | 35 | 24 | SLC9A3R2 |
| CGCGCTCTCA | 61 | 11 | 14 | 0 | SLC9A3R2 |
| GGAGCACACA | 89 | 20 | 7 | 5 | SLFN13 |
| TGTGGGAAAT | 3102 | 453 | 505 | 771 | SLPI |
| TGCTAGGAAG | 95 | 17 | 12 | 32 | SMPD2 |
| GAAAAATCAA | 107 | 17 | 26 | 15 | SNAPC3 |
| CCGCCTCCGG | 67 | 15 | 29 | 6 | SNRPN |
| AATATTATGT | 44 | 1 | 4 | 2 | SNTB1 |
| CATTTGTCAA | 369 | 6 | 34 | 18 | SNTN |
| TCAGATCTGC | 140 | 6 | 8 | 0 | SNTN |
| TTTGCAGAAG | 89 | 3 | 3 | 0 | SNTN |
| AAAAAGCAGA | 212 | 56 | 117 | 70 | SOD1 |
| CAAAATACTG | 51 | 17 | 15 | 17 | SOX30 |
| CCACTTGAAG | 66 | 3 | 5 | 3 | SPA17 |
| AGTTTGTTTA | 62 | 0 | 7 | 6 | SPA17 |
| AAAAATAATC | 50 | 2 | 2 | 0 | SPA17 |
| TTGAAAGCTC | 148 | 29 | 44 | 35 | SPAG1 |
| TGTGGGAGTA | 95 | 18 | 14 | 15 | SPAG16 |
| CATTTTTACT | 207 | 2 | 9 | 7 | SPAG6 |
| TAGAGCCACC | 74 | 0 | 4 | 0 | SPATA18 |
| GACAAGAAAC | 43 | 8 | 12 | 8 | SPATA4 |
| TGACTGTTCT | 44 | 3 | 0 | 0 | SPATS1 |
| ATTCAGCACC | 63 | 12 | 43 | 21 | SPCS1 |
| GTGCAGGGAG | 101 | 12 | 18 | 24 | SPDEF |
| GGCTTTAGGG | 217 | 44 | 75 | 51 | SPDYA |
| GGGGAATCTG | 63 | 1 | 4 | 6 | SPEF1 |
| TACAAGGAAG | 52 | 1 | 1 | 0 | SPEF2 |
| GTGGAGGAGG | 347 | 2 | 2 | 12 | SPTA1 |
| AATGTAATCA | 144 | 21 | 44 | 45 | SRI |
| AGCGTGTGAT | 173 | 9 | 34 | 40 | SSBP4 |
| AAATAAAAGA | 427 | 9 | 33 | 34 | SSR3 |
| GGACCCTCTC | 66 | 22 | 15 | 22 | ST6GAL1 |
| TAATCTTCTC | 78 | 4 | 8 | 3 | STBD1 |
| CTCTGGAAAT | 46 | 8 | 15 | 10 | STEAP3 |
| TGTTCGTGAG | 44 | 2 | 1 | 0 | STK33 |
| TAAGAATTGA | 45 | 3 | 5 | 10 | STOX1 |
| GAAGGCTTTA | 92 | 14 | 18 | 5 | SYNE1 |
| TTTAATAAAC | 74 | 16 | 9 | 17 | SYNGAP1 |
| TGCAGACCCA | 85 | 27 | 21 | 16 | TAX1BP1 |
| CTTTCAATGT | 84 | 25 | 26 | 23 | TBC1D8 |
| TTGTAAAGTA | 76 | 15 | 19 | 12 | TCEAL8 |
| CTGGACTGGG | 47 | 4 | 6 | 3 | TCTN1 |
| CCTGGCTGTA | 43 | 1 | 7 | 0 | TCTN2 |
| GGGTCTGGGC | 200 | 6 | 11 | 2 | TEKT1 |
| TAGACATAAA | 55 | 3 | 7 | 0 | TEKT1 |
| GACACGCGGC | 65 | 1 | 2 | 0 | TEKT2 |
| CTCCACCCGA | 2413 | 302 | 298 | 80 | TFF3 |
| CCTCAGGATA | 1783 | 483 | 656 | 418 | TGFA |
| TATATTTTCT | 166 | 17 | 76 | 26 | TGM2 |
| TTGGGAATCC | 74 | 20 | 40 | 22 | THNSL2 |
| AAGAAATCTG | 198 | 38 | 37 | 12 | TM4SF19 |
| CCACCCCGAA | 269 | 77 | 101 | 73 | TMBIM6 |
| AGCTTATTGA | 111 | 30 | 54 | 28 | TMEM14B |
| ACTATCTCTA | 79 | 3 | 39 | 24 | TMEM173 |
| TCCAAGTCCG | 467 | 7 | 40 | 16 | TMEM190 |
| GATGAATCCG | 52 | 13 | 5 | 2 | TMEM45B |
| AAGAAGCAGG | 223 | 69 | 91 | 54 | TMEM59 |
| CAGTTCTCTG | 734 | 191 | 213 | 106 | TMEM66 |
| AGAATAGCTT | 110 | 30 | 36 | 31 | TMEM67 |
| TTTTGTGTGC | 66 | 6 | 11 | 6 | TMEM67 |
| CTCTGAGTCC | 148 | 0 | 2 | 3 | TMEM77 |
| GATCTGATTG | 75 | 20 | 20 | 22 | TMF1 |
| ATTTCTTTGT | 44 | 2 | 2 | 3 | TMPRSS3 |
| GATCCTGATT | 72 | 3 | 13 | 6 | TNFAIP8L1 |
| GCTAACCCCT | 1810 | 47 | 96 | 93 | TPPP3 |
| ACCCTGAATG | 42 | 1 | 9 | 11 | TRAF3IP1 |
| TTACCTTACC | 147 | 28 | 42 | 32 | TRIP13 |
| AAATAAATTT | 41 | 12 | 14 | 0 | TSHR |
| AGCCAGGCCT | 41 | 2 | 4 | 0 | TSNAXIP1 |
| GGAACTGTGA | 1023 | 40 | 248 | 307 | TSPAN1 |
| AAATTATAAA | 173 | 3 | 4 | 3 | TSPAN19 |
| TAGACTAGCA | 568 | 125 | 181 | 118 | TSPAN3 |
| CCAAAGCTAT | 78 | 1 | 12 | 16 | TSPAN8 |
| TGCTTGAAGG | 94 | 9 | 19 | 11 | TSPYL4 |
| GGACTCTGAG | 78 | 1 | 3 | 3 | TTC18 |
| GGATTTTATT | 72 | 2 | 4 | 3 | TTC25 |
| TTACAGATAA | 130 | 0 | 2 | 5 | TTC29 |
| TGAAGAGTCT | 411 | 7 | 28 | 7 | TTLL10 |
| TTCATAGGTT | 62 | 0 | 4 | 0 | TTLL9 |
| AATGCTTTGT | 1564 | 72 | 254 | 170 | TUBA1A |
| GATAGTGTGG | 226 | 4 | 15 | 6 | TUBA4B |
| CTGTACAGAC | 2497 | 444 | 552 | 314 | TUBB2C |
| AAGATAAACT | 78 | 16 | 32 | 11 | TUSC3 |
| TACTGAAAAT | 63 | 15 | 17 | 7 | TUSC3 |
| GCTCCCTGTA | 44 | 1 | 4 | 2 | UBA52 |
| ATTTTTACTA | 105 | 19 | 96 | 3 | UBD |
| CTCTTACCTG | 98 | 3 | 4 | 2 | UBXN10 |
| ACTTCTCCTT | 44 | 1 | 1 | 0 | UBXN10 |
| GATGCCTCTG | 142 | 11 | 13 | 20 | UBXN11 |
| CTTTCCTTTT | 183 | 6 | 26 | 34 | UCP2 |
| CGATTCTGGA | 606 | 84 | 134 | 85 | UFC1 |
| AGACAGTAAT | 162 | 48 | 38 | 41 | UNC119B |
| AGTTTAAATG | 55 | 17 | 18 | 14 | VPS13B |
| ATCCAGTCTG | 300 | 9 | 18 | 6 | VSTM2L |
| ACTTTAACTG | 62 | 1 | 2 | 0 | VWA3A |
| TAACCTTGCT | 53 | 5 | 7 | 0 | WDR19 |
| CTGCAGGCCC | 56 | 10 | 9 | 15 | WDR34 |
| GCCAGGACTC | 180 | 6 | 9 | 6 | WDR38 |
| GGATAATTTA | 62 | 3 | 6 | 0 | WDR49 |
| TGTGCCTTTC | 42 | 3 | 4 | 2 | WDR52 |
| GTGAGTGTGT | 205 | 21 | 25 | 18 | WDR54 |
| AAAATGTATC | 41 | 9 | 8 | 6 | WDR60 |
| CTGAACATAT | 58 | 0 | 5 | 0 | WDR63 |
| ACAAAGTTAT | 51 | 0 | 2 | 0 | WDR69 |
| AAATTAAAAA | 417 | 56 | 59 | 40 | WDR70 |
| GATTTAGAGC | 116 | 5 | 8 | 0 | WDR78 |
| GGCATTTATT | 148 | 46 | 44 | 37 | WDR90 |
| CCTGCTTGTC | 483 | 26 | 183 | 94 | WFDC2 |
| ATGCATTGTT | 93 | 12 | 8 | 17 | WRB |
| GGTGACCACC | 76 | 0 | 2 | 0 | XIST |
| CTGATTTTTA | 52 | 18 | 46 | 5 | XRN2 |
| TCATCACACT | 51 | 0 | 3 | 0 | ZBBX |
| TAATAAAATA | 59 | 4 | 11 | 8 | ZC3H8 |
| CGGACCTTCA | 43 | 4 | 10 | 6 | ZDHHC1 |
| CCAAGGGAAT | 245 | 2 | 18 | 3 | ZMYND10 |
| AAATTATATT | 58 | 0 | 4 | 0 | ZNF214 |
| CAAATATAAA | 73 | 2 | 2 | 3 | ZNF451 |
| CTTATTATAC | 40 | 3 | 6 | 2 | ZNF569 |
| TAGCTTCTTC | 204 | 67 | 50 | 67 | ZNF789 |
| GCCAGAGGAG | 44 | 4 | 3 | 2 | ZSCAN10 |
| TAGTAAAGGC | 103 | 25 | 41 | 31 | ZSCAN18 |
| ACTAACACCC | 2678 | 562 | 873 | 539 |  |
| GCTAGGTTTA | 540 | 105 | 155 | 111 |  |
| CCACTGCTCT | 532 | 67 | 56 | 67 |  |
| GCGCGGGGGC | 445 | 25 | 8 | 6 |  |
| AGACCCACAA | 387 | 109 | 127 | 71 |  |
| GCCAGTCTGT | 356 | 4 | 0 | 0 |  |
| CTTGAGTCCA | 328 | 0 | 0 | 13 |  |
| TCTCTCTGGA | 243 | 3 | 15 | 2 |  |
| GCTACACAAT | 234 | 16 | 15 | 11 |  |
| CTAGGAAAAT | 225 | 6 | 35 | 0 |  |
| TTAGCTTGTT | 219 | 57 | 78 | 28 |  |
| CTTTGAGCCC | 214 | 3 | 2 | 10 |  |
| CTTGGAGTCC | 205 | 1 | 3 | 0 |  |
| TCTTTTTGTA | 197 | 2 | 9 | 42 |  |
| GCTTTGAGTC | 179 | 1 | 2 | 3 |  |
| CTTAAATCTG | 178 | 48 | 57 | 30 |  |
| AGGAGGGTTT | 168 | 1 | 1 | 10 |  |
| CCTTGAGTCC | 164 | 1 | 1 | 13 |  |
| GAGCCCTGGT | 160 | 5 | 10 | 6 |  |
| CTTTGAGTTC | 157 | 0 | 2 | 10 |  |
| GCAGCCTTGC | 133 | 0 | 0 | 0 |  |
| CGCCTGCCCT | 124 | 0 | 6 | 3 |  |
| TCAAGTTTTC | 120 | 31 | 18 | 25 |  |
| GGTCACTGTG | 117 | 2 | 8 | 8 |  |
| TGGGGGCCTC | 117 | 2 | 8 | 0 |  |
| GCAAAGAGTG | 102 | 1 | 6 | 0 |  |
| TAAACAGCAA | 101 | 1 | 7 | 0 |  |
| CTTTGAATCC | 99 | 0 | 0 | 6 |  |
| CCATACAGAC | 93 | 27 | 29 | 6 |  |
| CATTTGGAAC | 91 | 6 | 7 | 2 |  |
| ATAGATATGG | 91 | 2 | 7 | 0 |  |
| TCCTTATTAA | 90 | 21 | 7 | 15 |  |
| GCTATCAGTA | 88 | 6 | 14 | 0 |  |
| TGGAGCTATG | 87 | 12 | 14 | 15 |  |
| TATCCCGGAA | 86 | 16 | 6 | 19 |  |
| GCAGCCTCAT | 86 | 1 | 1 | 0 |  |
| CTTACAAGCA | 82 | 22 | 41 | 0 |  |
| TTTTGAGTCC | 78 | 0 | 0 | 6 |  |
| AGCAACAATA | 75 | 0 | 2 | 2 |  |
| CTTTTGAGTC | 73 | 0 | 0 | 0 |  |
| TCTTAAAAAA | 73 | 12 | 9 | 16 |  |
| GGACCTTTAT | 73 | 5 | 9 | 2 |  |
| GAGGATTCCA | 72 | 3 | 2 | 0 |  |
| AATATACTAG | 71 | 1 | 2 | 0 |  |
| CTGTGATGCA | 70 | 0 | 9 | 2 |  |
| TGAATGTATT | 70 | 3 | 10 | 2 |  |
| TGTGAAGATT | 69 | 5 | 8 | 19 |  |
| TATCCCTGGT | 68 | 0 | 2 | 2 |  |
| GAAATACGTA | 67 | 0 | 3 | 0 |  |
| GTTTGAGTCC | 67 | 0 | 1 | 0 |  |
| CAACACTTCA | 66 | 6 | 13 | 15 |  |
| TAATATAACA | 65 | 1 | 3 | 3 |  |
| TGATTCTGCC | 64 | 0 | 3 | 0 |  |
| TCCCCTATTA | 63 | 8 | 9 | 2 |  |
| TGACATTAAA | 62 | 10 | 11 | 13 |  |
| CTGTTGTTGG | 61 | 18 | 31 | 0 |  |
| TAGTAGAGTG | 61 | 11 | 16 | 16 |  |
| GCGTGGGTGG | 60 | 0 | 7 | 3 |  |
| CTTTTACTAT | 60 | 6 | 8 | 3 |  |
| TGCTATAAGG | 60 | 2 | 6 | 0 |  |
| GCACCATAAT | 60 | 5 | 19 | 6 |  |
| AATGCTTGTG | 60 | 4 | 6 | 0 |  |
| TGTTGTATGA | 59 | 1 | 1 | 0 |  |
| TGACTGTAGC | 59 | 0 | 2 | 0 |  |
| GTGCTATGCC | 58 | 7 | 14 | 3 |  |
| TATAGTTGGA | 57 | 0 | 2 | 0 |  |
| AGGAAGACTG | 57 | 18 | 11 | 15 |  |
| CTAAGCGCAG | 57 | 15 | 11 | 5 |  |
| CTGCAAATAA | 56 | 2 | 6 | 0 |  |
| CACTTTTAAA | 56 | 5 | 1 | 0 |  |
| TGGACAAGCT | 56 | 1 | 21 | 5 |  |
| GCTCTTAGAG | 56 | 1 | 13 | 3 |  |
| TCTCTATTAA | 55 | 10 | 15 | 16 |  |
| CAATAGTCAA | 53 | 6 | 6 | 0 |  |
| ATGCATTGCA | 50 | 3 | 2 | 0 |  |
| GCACTGAGCA | 50 | 1 | 6 | 3 |  |
| TTTGTTGCTG | 49 | 5 | 4 | 0 |  |
| GGCCCACTGT | 49 | 4 | 3 | 3 |  |
| CATTATTTCT | 49 | 1 | 5 | 0 |  |
| TTGATGTACC | 48 | 0 | 2 | 0 |  |
| TACTCCTAGG | 48 | 2 | 2 | 3 |  |
| TTTCATACAC | 48 | 9 | 12 | 2 |  |
| TCAGTATGTG | 47 | 1 | 2 | 0 |  |
| AAGTTTTCCT | 46 | 8 | 10 | 16 |  |
| GCTTTGCTCT | 46 | 2 | 5 | 6 |  |
| TATTATAAAA | 46 | 14 | 33 | 15 |  |
| TAAAAACAAT | 46 | 0 | 4 | 2 |  |
| GTTTGAATCC | 46 | 0 | 1 | 0 |  |
| CATCTGAAAT | 45 | 1 | 3 | 0 |  |
| TCTCATTTAG | 45 | 0 | 2 | 0 |  |
| CAGAGCAATT | 45 | 0 | 3 | 0 |  |
| CAAAGAGGGT | 44 | 0 | 4 | 0 |  |
| CCATTGAAAA | 44 | 3 | 4 | 6 |  |
| ATGATAATGG | 44 | 9 | 3 | 11 |  |
| CTTATCAGTA | 44 | 1 | 6 | 0 |  |
| CTGTGAGTCC | 43 | 0 | 0 | 0 |  |
| CTTAGAGTCC | 43 | 0 | 0 | 3 |  |
| CTTTAAGTCC | 42 | 0 | 0 | 0 |  |
| ACTGGCTAAA | 42 | 0 | 3 | 0 |  |
| TTTTCAGATG | 42 | 1 | 2 | 5 |  |
| AGCATCTCAA | 41 | 0 | 0 | 0 |  |
| TGATAAATTA | 41 | 3 | 3 | 2 |  |
| CAATCTTGTA | 41 | 0 | 2 | 0 |  |
| AAAACAAGTC | 40 | 3 | 8 | 13 |  |
| TAATCCAAAA | 40 | 6 | 8 | 6 |  |
| GGCAAAATTA | 40 | 9 | 10 | 6 |  |

^1^Tags with a three-fold or greater decrease in average normalized tag counts in both CIS and PC relative to BE; a minimal abundance of 40 TPM in BE (678 tags in total).

^2^Average normalized tag counts, expressed as tags per million (TPM) for 14 bronchial epithelial libraries.

^3^Average normalized tag counts (TPM) for five carcinoma-in-situ libraries.

^4^Average normalized tag counts (TPM) for six invasive cancer libraries.

^5^Average normalized tag counts (TPM) for two precancerous libraries.

^6^Tag-to-gene mapping was according to SAGE Genie, “Best Gene for Tag”, September 17, 2009 version. No entry is given for tags that map to transcript sequences within the database of Unclustered ESTs.
