# Supplementary material for: Transcriptome Profiles of Carcinoma-in-Situ and Invasive Non-Small Cell Lung Cancer as Revealed by SAGE
Source: PLoS One. 2010 Feb 11;5(2):e9162. doi: 10.1371/journal.pone.0009162 (PMC2820080; doi:10.1371/journal.pone.0009162)
Supplement: Table S5 — Up-regulated gene expression changes in carcinoma-in-situ relative to bronchial epithelium and precancerous lesions. (0.34 MB DOC) [file pone.0009162.s005.doc]

**Table S5. Up-regulated gene expression changes in carcinoma-in-situ relative to bronchial epithelium and precancerous lesions.**

| **Tag^1^** | **BE Mean^2^** | **CIS Mean^3^** | **SCC Mean^4^** | **PC Av^5^** | **Gene Symbol^6^** |
| --- | --- | --- | --- | --- | --- |
| TTTGCTGAAC | 37 | 149 | 80 | 46 | ABCC5 |
| GCTTGAATAA | 57 | 391 | 429 | 37 | AKR1B10 |
| AGGTCTGCCA | 217 | 651 | 541 | 77 | AKR1C1 |
| AGGTCTACCA | 88 | 271 | 153 | 2 | AKR1C1 |
| GAGAGCTTTG | 54 | 167 | 225 | 35 | AKR1C3 |
| GAGGGCTTTG | 25 | 128 | 95 | 0 | AKR1C3 |
| CCTTGAGTAC | 7 | 45 | 47 | 10 | ALDOC |
| GGAATAAAGC | 5 | 295 | 92 | 0 | APOC2 |
| GGAGCTGGCC | 4 | 95 | 42 | 3 | ARTN |
| TAGGATGGGG | 40 | 247 | 160 | 77 | ATP1B3 |
| GGGGGTCACC | 41 | 189 | 132 | 44 | ATP5G1 |
| GAAAGATGCT | 21 | 78 | 56 | 20 | BEX2 |
| TTTGACAATA | 22 | 83 | 35 | 24 | BNIP2 |
| AGTCTGCTGG | 0 | 152 | 16 | 25 | C10orf99 |
| GGGCCCCAAA | 22 | 133 | 139 | 39 | C19orf48 |
| TGTTCTATTA | 0 | 55 | 4 | 8 | C2orf63 |
| AACAATAAAA | 17 | 79 | 47 | 20 | C2orf88 |
| TTTCCAGCAC | 1 | 75 | 3 | 0 | C6orf15 |
| GGAGGTGTGG | 19 | 177 | 21 | 18 | CCDC34 |
| CTACAAGACG | 3 | 61 | 24 | 5 | CD27 |
| TGAGTATTAA | 6 | 118 | 124 | 8 | CHST2 |
| CAATGAGCAA | 8 | 45 | 18 | 7 | CHST3 |
| TTAAAAGCCT | 80 | 301 | 328 | 60 | CKS1B |
| AGCAAGAAAC | 4 | 70 | 16 | 2 | CLDND1 |
| ACAACAGACA | 0 | 46 | 8 | 0 | CLK2P |
| TTTGTTTCTG | 2 | 97 | 1 | 9 | CTAG1B |
| CAGGTTTCAT | 3 | 352 | 195 | 85 | CXCL14 |
| GGGAAATCTG | 11 | 52 | 3 | 3 | CYP26A1 |
| GCGCATCAAA | 5 | 64 | 7 | 13 | DAPL1 |
| CAAGGGCCCA | 22 | 175 | 114 | 3 | DERL3 |
| CTTTCTTTGA | 17 | 73 | 116 | 24 | DKK3 |
| AGAGTCATAC | 2 | 391 | 40 | 86 | DSC2 |
| GGGTATTGGT | 0 | 61 | 8 | 8 | DSC2 |
| GACACTAGAG | 0 | 62 | 11 | 13 | DSC3 |
| GTACAGAAAT | 0 | 42 | 2 | 10 | DSG1 |
| GTAAATATGG | 25 | 373 | 196 | 102 | DST |
| AACCAATACA | 5 | 112 | 97 | 22 | ECE2 |
| TGTACATTCT | 19 | 78 | 86 | 24 | EFNA1 |
| GGCTTAAAAA | 13 | 50 | 33 | 7 | EIF4EBP2 |
| GTTTTCTTTC | 20 | 70 | 57 | 3 | ETNK2 |
| TTACTTCCCC | 4 | 51 | 110 | 5 | FADS2 |
| CTGGGCCCCT | 0 | 71 | 25 | 0 | FAM116B |
| CAAGCTCTGC | 0 | 44 | 1 | 2 | FAM20B |
| TAAAATGTTG | 7 | 69 | 119 | 17 | FAM43A |
| TGATCTCCAA | 23 | 77 | 61 | 21 | FASN |
| CCGTGGTCGT | 46 | 137 | 144 | 30 | FBL |
| TATCTGCCAA | 9 | 48 | 51 | 5 | FBXO27 |
| GAGAGATGCC | 0 | 60 | 14 | 19 | FETUB |
| AATTTCCAGT | 4 | 102 | 20 | 0 | FGFBP2 |
| TACAGATCAC | 5 | 75 | 57 | 5 | FZD7 |
| GCCACCCCGT | 22 | 85 | 106 | 21 | G6PD |
| GTAGCGCACG | 12 | 65 | 24 | 22 | GLTP |
| GATTTCTTTG | 9 | 83 | 63 | 9 | GPC3 |
| ACATTCTTTT | 48 | 719 | 970 | 199 | GPNMB |
| GGTGGTGTCT | 124 | 1138 | 585 | 160 | GPX2 |
| GTCCCTGCCT | 124 | 811 | 427 | 57 | GSTM1 |
| TGCCGTTTTG | 2 | 192 | 94 | 2 | GSTM3 |
| GGAGTTTAGT | 0 | 44 | 12 | 0 | GSTM3 |
| CAGGCCCTTT | 11 | 42 | 24 | 2 | GSTM4 |
| AGCTTCTACC | 0 | 1006 | 99 | 283 | HCG9 |
| TATAATGTGC | 4 | 48 | 13 | 2 | IGF2BP3 |
| GAAATAAAGC | 62 | 38019 | 14716 | 568 | IGHG1 |
| CTCCCCCAAG | 33 | 7043 | 1969 | 32 | IGHG1 |
| CTCCCCCAAA | 29 | 4772 | 1604 | 19 | IGHG1 |
| GCGGAGGTGG | 2 | 504 | 176 | 3 | IGHG1 |
| CAAACTAACC | 10 | 466 | 516 | 2 | IGHG1 |
| AGGCTCTGCA | 0 | 190 | 57 | 3 | IGHG1 |
| AAATGATCCC | 2 | 89 | 134 | 6 | IGHG1 |
| CGTGGTGGTG | 5 | 57 | 54 | 0 | IGHG1 |
| CCACGTGAAG | 0 | 50 | 15 | 0 | IGHG1 |
| CCTTCCAGGT | 0 | 44 | 10 | 0 | IGHG1 |
| GTACGTATTC | 28 | 877 | 457 | 22 | IGJ |
| GAAGCCCCAG | 0 | 300 | 101 | 0 | IGKC |
| AGGGTCCCCG | 1 | 266 | 97 | 0 | IGKC |
| GAAACCCCAG | 6 | 214 | 131 | 11 | IGKC |
| AGGGTCCCTG | 0 | 164 | 37 | 0 | IGKC |
| CAAGCTCTAC | 4 | 69 | 96 | 0 | IGKC |
| AAGGGAGCAC | 31 | 5039 | 3229 | 32 | IGL@ |
| AAACCCCAAT | 18 | 4949 | 1692 | 280 | IGL@ |
| CCTAAGTGAC | 12 | 53 | 31 | 15 | IL23A |
| GTGCGCCGGA | 1 | 47 | 15 | 15 | IQCF6 |
| AATCTGCGCC | 28 | 136 | 82 | 45 | ISG15 |
| GAAACTAGGA | 16 | 189 | 62 | 19 | KCNS3 |
| TGACAATAAA | 0 | 73 | 4 | 5 | KPRP |
| ACCTCCACTG | 0 | 698 | 0 | 10 | KRTDAP |
| TTCCCTTACC | 1 | 226 | 2 | 17 | LCE3D |
| GCCAGGTTGC | 2 | 41 | 49 | 13 | LEPREL1 |
| AGAAGATGTT | 0 | 117 | 31 | 2 | LOC100132634 |
| ATGGCAGAAG | 7 | 56 | 48 | 15 | LOC284889 |
| TCCTGGCAAA | 4 | 62 | 15 | 15 | LOC389904 |
| GACTCGCTCC | 5 | 76 | 30 | 8 | LY6K |
| TAAGAGAAAT | 0 | 54 | 9 | 0 | MAGEA9 |
| ACTTTAGCCT | 9 | 43 | 32 | 10 | MAP4K1 |
| CGGATTATCC | 14 | 52 | 37 | 6 | MCM2 |
| CTGCACTTAC | 45 | 225 | 225 | 61 | MCM7 |
| AATGTAGCTG | 4 | 41 | 3 | 5 | MIAT |
| TTGAAACTGT | 0 | 67 | 61 | 0 | MID1 |
| AACGCGGCCA | 163 | 690 | 666 | 232 | MIF |
| GAAATAAGCA | 0 | 65 | 37 | 3 | MUC17 |
| GAAATAAAGT | 50 | 352 | 275 | 102 | NAMPT |
| GGACTTTCCT | 5 | 98 | 121 | 15 | NDRG1 |
| CAAGCCACAG | 0 | 52 | 64 | 5 | NDUFA4L2 |
| GAAAAATTTA | 66 | 259 | 277 | 88 | NGFRAP1 |
| TGTGCCACTA | 23 | 72 | 67 | 24 | NMD3 |
| AGAAGACATT | 1 | 40 | 15 | 2 | NOC3L |
| GCAGAATAGA | 10 | 54 | 20 | 0 | NOTCH3 |
| TTAAATTAAT | 2 | 51 | 48 | 0 | NTRK2 |
| TGATTGTGAT | 24 | 398 | 17 | 3 | NTS |
| AATCTCTCAA | 16 | 318 | 13 | 0 | NTS |
| TGCAACTGTA | 4 | 47 | 20 | 15 | NXF4 |
| ATGCAGCCAT | 28 | 146 | 128 | 36 | ODC1 |
| CTGCCATTAA | 2 | 89 | 4 | 0 | OR14J1 |
| GTTAAATGCA | 9 | 51 | 58 | 2 | OXCT1 |
| TTTTATATCA | 14 | 55 | 26 | 6 | PERP |
| CGTGACCTGG | 0 | 112 | 45 | 3 | PHYHIP |
| TTGAATCCCC | 42 | 952 | 115 | 193 | PI3 |
| TGTGGAAACC | 67 | 200 | 101 | 30 | PIM2 |
| TTTGTAGAGG | 15 | 648 | 239 | 153 | PKP1 |
| TGGGTGAAAA | 1 | 45 | 5 | 3 | PLA2G4E |
| TTAGTTTTTA | 4 | 57 | 88 | 11 | PLAT |
| GTCAAAAAAA | 30 | 116 | 23 | 15 | POLI |
| GACTTTTCTG | 5 | 69 | 16 | 2 | POU2AF1 |
| GGGAAGGGAC | 3 | 361 | 128 | 118 | PPP2R1B |
| CTGGGTGCCT | 0 | 527 | 172 | 3 | PSMB4 |
| CATCCTGCTG | 64 | 208 | 209 | 47 | PSMD2 |
| ACGGAACAAT | 3 | 43 | 37 | 3 | PTGDS |
| GAATAAAGCA | 1 | 147 | 79 | 9 | PTPLB |
| TGTATACAAT | 9 | 44 | 42 | 0 | RAB6A |
| TAAATAAATG | 21 | 64 | 18 | 15 | RAD1 |
| CTGGGCGCCC | 0 | 169 | 91 | 6 | RHD |
| ATTAAAGTCA | 4 | 41 | 5 | 6 | RIMS3 |
| AAGAAACGAG | 0 | 40 | 4 | 10 | RNASE7 |
| TAATTTTTAA | 4 | 45 | 26 | 14 | RRM2 |
| GACTCTTCAG | 15 | 57 | 661 | 12 | SERPINA3 |
| TGCTTTATTG | 5 | 68 | 6 | 2 | SLC16A14 |
| GAGACTCCTG | 79 | 432 | 633 | 27 | SLC2A1 |
| GACTGTTGCT | 14 | 69 | 22 | 10 | SLC44A1 |
| TCATTTTCCA | 27 | 443 | 139 | 7 | SLC6A8 |
| AGTGCTCACT | 3 | 118 | 94 | 11 | SLC6A8 |
| GAAATAAGGC | 2 | 246 | 77 | 0 | SLCO1A2 |
| GGTTGAAAAA | 11 | 317 | 348 | 15 | SNAR-E |
| CTGATTTTTG | 11 | 64 | 13 | 8 | SPAG4 |
| GGCTTCTAAC | 0 | 903 | 35 | 31 | SPRR2E |
| AGCTTCCACC | 1 | 91 | 1 | 20 | SPRR2F |
| GTCAAGCCCA | 0 | 165 | 1 | 2 | SPRR2G |
| GTGATGTAAG | 22 | 123 | 295 | 11 | SRXN1 |
| AGACTAACAC | 22 | 76 | 39 | 10 | TESK1 |
| GAAGTTTTTT | 17 | 76 | 56 | 12 | THOC3 |
| ACGCCTACTG | 0 | 61 | 1 | 2 | TKTL1 |
| ACCCGCCGGG | 69 | 249 | 612 | 84 | TNNC2 |
| CAATAAAATT | 22 | 453 | 149 | 89 | TP63 |
| ACTGTCTCCA | 9 | 50 | 50 | 6 | TPD52L1 |
| AGTATGTATG | 11 | 49 | 110 | 7 | TRIB3 |
| CCAACAAGAA | 4 | 68 | 169 | 17 | TSPAN7 |
| CTCCCCCAGC | 0 | 43 | 7 | 0 | TTBK1 |
| TAACCAAGAG | 0 | 42 | 0 | 0 | TTR |
| CTGGCGAGCG | 13 | 57 | 82 | 10 | UBE2S |
| GAAATGAAGC | 0 | 82 | 30 | 0 | UTRN |
| TAGATTCAAC | 13 | 41 | 46 | 12 | VKORC1L1 |
| TGGCAGTCTG | 1 | 57 | 11 | 7 | ZNF277 |
| GGAGACTTTT | 24 | 86 | 49 | 12 | ZNF302 |
| CTGGGTGCCC | 19 | 331 | 43 | 44 | ZNF335 |
| AACCCGGGAA | 19 | 948 | 62 | 217 |  |
| AGAAGACGTT | 1 | 730 | 240 | 0 |  |
| AGTGCAGGGA | 1 | 692 | 194 | 29 |  |
| GAAATCCAAA | 32 | 563 | 69 | 42 |  |
| TTGCTCAAAA | 23 | 479 | 74 | 57 |  |
| AAATAAAGCA | 1 | 392 | 174 | 0 |  |
| GGAAATAAAG | 3 | 309 | 134 | 2 |  |
| TTGCTCACAA | 60 | 285 | 61 | 65 |  |
| TTGCTCACAC | 36 | 250 | 69 | 66 |  |
| GAGATAAAGC | 0 | 250 | 78 | 3 |  |
| ACCCTGCCAA | 58 | 200 | 156 | 56 |  |
| GAAATAGAGC | 0 | 190 | 57 | 0 |  |
| GAAATAAACC | 0 | 183 | 38 | 0 |  |
| GCTCCCCCAA | 0 | 174 | 25 | 0 |  |
| GAAGTAAAGC | 1 | 173 | 43 | 6 |  |
| GCTCCGAGCG | 54 | 171 | 178 | 15 |  |
| GATCTCGCAA | 51 | 170 | 102 | 48 |  |
| GAGTTTATTC | 1 | 170 | 35 | 0 |  |
| CTCCCCCCAA | 1 | 144 | 28 | 0 |  |
| AGCGCTGATT | 0 | 125 | 10 | 0 |  |
| GAAATAAAAC | 3 | 124 | 41 | 15 |  |
| GCGTGCTCTC | 33 | 115 | 184 | 15 |  |
| GAAACAAAGC | 1 | 95 | 20 | 0 |  |
| GAAATTAAGC | 0 | 89 | 25 | 6 |  |
| TGCTGTGTCC | 9 | 86 | 46 | 20 |  |
| AAACCCCCAA | 1 | 83 | 8 | 3 |  |
| GGAGGCGTGG | 0 | 83 | 19 | 0 |  |
| TAAATGTGCA | 4 | 79 | 46 | 9 |  |
| TATGCCTTTT | 14 | 71 | 35 | 20 |  |
| TTCCCCCAAG | 0 | 70 | 12 | 0 |  |
| GGTGGGTTTA | 1 | 69 | 25 | 2 |  |
| GCGCACCGGA | 4 | 65 | 3 | 20 |  |
| CTTCCTTGCT | 2 | 65 | 34 | 18 |  |
| CTCCCCAAAA | 0 | 62 | 25 | 0 |  |
| TGAAATAAAC | 17 | 59 | 34 | 15 |  |
| CCGACGGGCG | 19 | 58 | 237 | 12 |  |
| CTGAAGATTT | 5 | 58 | 5 | 5 |  |
| TATAATACCT | 5 | 58 | 22 | 10 |  |
| CACAGGCATC | 0 | 58 | 0 | 0 |  |
| GAAATAAAGA | 2 | 57 | 12 | 2 |  |
| TGCCTGTGGC | 17 | 55 | 31 | 2 |  |
| TTCCCCCAAA | 0 | 53 | 13 | 2 |  |
| GTAGTTACTG | 17 | 51 | 31 | 3 |  |
| CCAGGGCCAG | 0 | 50 | 17 | 0 |  |
| TGAATGTCAC | 2 | 50 | 46 | 0 |  |
| CTTCCCTGCC | 0 | 48 | 18 | 11 |  |
| AGGGTGTCCT | 0 | 48 | 7 | 0 |  |
| TTGCTCAAAC | 0 | 48 | 4 | 0 |  |
| GAAAATAAGC | 0 | 47 | 25 | 0 |  |
| GAAATAATGC | 0 | 46 | 3 | 0 |  |
| CAAATAAAGC | 1 | 46 | 15 | 0 |  |
| GCAATAAAGC | 1 | 46 | 6 | 5 |  |
| TTGACTCAGC | 0 | 46 | 6 | 12 |  |
| AGGGGAGCAC | 0 | 45 | 45 | 0 |  |
| GAATTAAAGC | 0 | 45 | 22 | 0 |  |
| TCAACACAGT | 5 | 44 | 21 | 13 |  |
| CCCCCCCAAG | 0 | 43 | 12 | 0 |  |
| ACGTGTGTAA | 0 | 43 | 1 | 10 |  |
| GATATAAAGC | 1 | 43 | 11 | 0 |  |
| TTCCAAAGCA | 1 | 41 | 4 | 6 |  |
| AGGAAGTCTT | 1 | 41 | 1 | 0 |  |
| CTAATGTTGG | 0 | 41 | 6 | 0 |  |
| GAAATACCCA | 1 | 40 | 1 | 0 |  |

^1^Tags with a three-fold or greater abundance in average normalized tag counts in CIS relative to both BE and PC; a minimal abundance of 40 TPM in CIS (225 tags in total).

^2^Average normalized tag counts, expressed as tags per million (TPM) for 14 bronchial epithelial libraries.

^3^Average normalized tag counts (TPM) for five carcinoma-in-situ libraries.

^4^Average normalized tag counts (TPM) for six invasive cancer libraries.

^5^Average normalized tag counts (TPM) for two precancerous libraries.

^6^Tag-to-gene mapping according to SAGE Genie “Best Gene for Tag”, September 17, 2009 version. No entry is given for tags that map to transcript sequences within the databases of Full-Length Set, UniGene Consensus, or Unclustered ESTs.
