# Supplementary material for: Transcriptome Profiles of Carcinoma-in-Situ and Invasive Non-Small Cell Lung Cancer as Revealed by SAGE
Source: PLoS One. 2010 Feb 11;5(2):e9162. doi: 10.1371/journal.pone.0009162 (PMC2820080; doi:10.1371/journal.pone.0009162)
Supplement: Table S6 — Up-regulated gene expression changes in invasive cancer relative to bronchial epithelium and precancerous lesions. (0.35 MB DOC) [file pone.0009162.s006.doc]

**Table S6. Up-regulated gene expression changes in invasive cancer relative to bronchial epithelium and precancerous lesions.**

| **Tag^1^** | **BE Mean^2^** | **CIS Mean^3^** | **SCC Mean^4^** | **PC Av^5^** | **Gene Symbol^6^** |
| --- | --- | --- | --- | --- | --- |
| TCTTGATTTA | 7 | 29 | 122 | 38 | A2M |
| GAAAAATAAA | 20 | 10 | 87 | 3 | ABCA4 |
| AAGATCAAGA | 4 | 14 | 43 | 0 | ACTA1 |
| AAAGAGAAAG | 4 | 119 | 137 | 43 | ADM |
| GCTTGAATAA | 57 | 391 | 429 | 37 | AKR1B10 |
| GAGAGCTTTG | 54 | 167 | 225 | 35 | AKR1C3 |
| GAGGGCTTTG | 25 | 128 | 95 | 0 | AKR1C3 |
| CCTTGAGTAC | 7 | 45 | 47 | 10 | ALDOC |
| CCGTCATCCT | 18 | 43 | 55 | 14 | ALG3 |
| CTATAGGAGA | 17 | 23 | 67 | 2 | ANTXR1 |
| GGAATAAAGC | 5 | 295 | 92 | 0 | APOC2 |
| CGACCCCACG | 48 | 44 | 196 | 65 | APOE |
| TTGGAACAAT | 28 | 21 | 388 | 118 | ARHGAP19 |
| GGAGCTGGCC | 4 | 95 | 42 | 3 | ARTN |
| GGGGGTCACC | 41 | 189 | 132 | 44 | ATP5G1 |
| GCCTGTCCCT | 0 | 5 | 43 | 10 | BGN |
| GGGCCCCAAA | 22 | 133 | 139 | 39 | C19orf48 |
| GAAATTTGAA | 17 | 44 | 61 | 12 | C1orf56 |
| AAATCAATAC | 57 | 72 | 258 | 68 | C1QC |
| TTCTGTGCTG | 4 | 21 | 140 | 34 | C1R |
| ACTGAAAGAA | 2 | 33 | 148 | 13 | C1S |
| CAGCATCTAA | 73 | 184 | 299 | 90 | C20orf24 |
| ACAAGTACCC | 4 | 20 | 68 | 16 | C5orf13 |
| TCATCTTCAA | 16 | 29 | 86 | 3 | CALR |
| GAGGGTTTAG | 7 | 9 | 40 | 0 | CCL20 |
| GACCCGCTGG | 0 | 18 | 66 | 12 | CCL26 |
| AAAAATCGGC | 34 | 50 | 104 | 25 | CCL5 |
| ACAGCCTGCA | 8 | 22 | 47 | 16 | CDC25B |
| GAAGGAAGAA | 15 | 26 | 58 | 20 | CDK4 |
| CCCGCATAGA | 4 | 22 | 45 | 13 | CDKN2A |
| TGAGTATTAA | 6 | 118 | 124 | 8 | CHST2 |
| TTAAAAGCCT | 80 | 301 | 328 | 60 | CKS1B |
| TGGATATCAG | 44 | 75 | 218 | 31 | CLDN1 |
| TTTTTCTATT | 5 | 38 | 70 | 6 | CLDN1 |
| CGTTTTATAT | 5 | 11 | 43 | 6 | CLDN1 |
| GACTCTGGGA | 58 | 96 | 248 | 31 | CLPTM1L |
| ATATGGATTT | 27 | 29 | 99 | 18 | CLPTM1L |
| TTTGTGTTGT | 0 | 11 | 63 | 17 | COL12A1 |
| GATATGTTAT | 3 | 14 | 40 | 2 | COL17A1 |
| GACTTTGGAA | 1 | 2 | 45 | 7 | COL1A1 |
| GATGAGGAGA | 2 | 18 | 321 | 39 | COL1A2 |
| GATCAGGCCA | 0 | 13 | 284 | 7 | COL3A1 |
| GACCGCAGGA | 1 | 46 | 285 | 27 | COL4A1 |
| TGATTCTGTT | 0 | 1 | 46 | 7 | COL5A1 |
| ACTTTAGATG | 0 | 18 | 159 | 35 | COL6A3 |
| TTTACAAAGA | 14 | 18 | 43 | 12 | CPE |
| GAGAGAGACT | 0 | 1 | 127 | 2 | CRP |
| TTCATAGCTG | 18 | 58 | 82 | 27 | CRTAP |
| GTACACACCC | 0 | 0 | 78 | 0 | CST1 |
| AGTGTCTGTG | 9 | 14 | 63 | 3 | CYR61 |
| ACTTATTATG | 3 | 50 | 120 | 30 | DCN |
| ACCGGGAGGT | 16 | 35 | 65 | 19 | DDX49 |
| CAAGGGCCCA | 22 | 175 | 114 | 3 | DERL3 |
| CTTTCTTTGA | 17 | 73 | 116 | 24 | DKK3 |
| CAGCTCCGCT | 14 | 19 | 41 | 6 | DUT |
| AACCAATACA | 5 | 112 | 97 | 22 | ECE2 |
| TGTACATTCT | 19 | 78 | 86 | 24 | EFNA1 |
| GCAAAATAAC | 4 | 18 | 41 | 11 | EIF5A |
| GGTGAGCGTG | 17 | 81 | 114 | 30 | EPHB3 |
| TTTATTATTG | 2 | 9 | 43 | 2 | ESM1 |
| TTACTTCCCC | 4 | 51 | 110 | 5 | FADS2 |
| TAAAATGTTG | 7 | 69 | 119 | 17 | FAM43A |
| CCGTGGTCGT | 46 | 137 | 144 | 30 | FBL |
| GCCTCAGTTC | 5 | 13 | 94 | 0 | FBLN1 |
| ACAGAATGCC | 2 | 34 | 65 | 16 | FBLN1 |
| CAGCTGGCCA | 4 | 15 | 49 | 11 | FBLN1 |
| TGCAATATGC | 4 | 31 | 74 | 21 | FBN1 |
| TATCTGCCAA | 9 | 48 | 51 | 5 | FBXO27 |
| AGTGGTGGCT | 23 | 40 | 95 | 20 | FMOD |
| ATCTTGTTAC | 11 | 8 | 425 | 109 | FN1 |
| TTTTCCTGTA | 15 | 15 | 46 | 7 | FNDC3B |
| TTCCCCCTTC | 7 | 9 | 54 | 17 | FOXD4 |
| TACAGATCAC | 5 | 75 | 57 | 5 | FZD7 |
| GCCACCCCGT | 22 | 85 | 106 | 21 | G6PD |
| CGGGTAGTAT | 17 | 13 | 58 | 17 | GAA |
| GTGCTCATTC | 16 | 7 | 59 | 5 | GDF15 |
| GAAAAGCCTT | 62 | 179 | 338 | 113 | GGH |
| GGAGAGGAAG | 11 | 19 | 60 | 6 | GLIS2 |
| GATTTCTTTG | 9 | 83 | 63 | 9 | GPC3 |
| ACATTCTTTT | 48 | 719 | 970 | 199 | GPNMB |
| GGTGGTGTCT | 124 | 1138 | 585 | 160 | GPX2 |
| GTGACCACGG | 446 | 491 | 2087 | 575 | GRIN2C |
| GTCCCTGCCT | 124 | 811 | 427 | 57 | GSTM1 |
| TGCCGTTTTG | 2 | 192 | 94 | 2 | GSTM3 |
| GCCACCCCCT | 1 | 5 | 40 | 0 | H19 |
| CTGAACTGCA | 0 | 0 | 133 | 2 | HLA-G |
| GAGGAAGAAG | 18 | 30 | 65 | 18 | HSP90B1 |
| GACGACTGAC | 11 | 20 | 64 | 9 | IFI16 |
| CGCCGACGAT | 165 | 398 | 528 | 89 | IFI6 |
| ACCATTGGAT | 24 | 39 | 122 | 18 | IFITM1 |
| GAAATAAAGC | 62 | 38019 | 14716 | 568 | IGHG1 |
| CTCCCCCAAG | 33 | 7043 | 1969 | 32 | IGHG1 |
| CTCCCCCAAA | 29 | 4772 | 1604 | 19 | IGHG1 |
| CAAACTAACC | 10 | 466 | 516 | 2 | IGHG1 |
| GCGGAGGTGG | 2 | 504 | 176 | 3 | IGHG1 |
| AAATGATCCC | 2 | 89 | 134 | 6 | IGHG1 |
| AGGCTCTGCA | 0 | 190 | 57 | 3 | IGHG1 |
| CGTGGTGGTG | 5 | 57 | 54 | 0 | IGHG1 |
| GTACGTATTC | 28 | 877 | 457 | 22 | IGJ |
| GAAACCCCAG | 6 | 214 | 131 | 11 | IGKC |
| GAAGCCCCAG | 0 | 300 | 101 | 0 | IGKC |
| AGGGTCCCCG | 1 | 266 | 97 | 0 | IGKC |
| CAAGCTCTAC | 4 | 69 | 96 | 0 | IGKC |
| AAGGGAGCAC | 31 | 5039 | 3229 | 32 | IGL@ |
| AAACCCCAAT | 18 | 4949 | 1692 | 280 | IGL@ |
| TCCTCTTTCC | 8 | 8 | 51 | 0 | IL32 |
| CAACTGCCCC | 12 | 27 | 40 | 8 | IRF6 |
| ACCCACGTCA | 28 | 31 | 83 | 15 | JUNB |
| GAAACTAGGA | 16 | 189 | 62 | 19 | KCNS3 |
| GAAAAGGGTT | 15 | 24 | 81 | 23 | LAPTM4B |
| TCTTGTGCAT | 33 | 240 | 306 | 86 | LDHA |
| GCCAGGTTGC | 2 | 41 | 49 | 13 | LEPREL1 |
| GAACCATTCA | 12 | 39 | 61 | 0 | LOC100131354 |
| ATGGCAGAAG | 7 | 56 | 48 | 15 | LOC284889 |
| TCGTAATAGT | 11 | 37 | 46 | 6 | LOC284889 |
| GAGCAAAGGA | 5 | 38 | 87 | 20 | LOC440731 |
| CCGAGGCTTG | 12 | 38 | 92 | 27 | LOC440995 |
| CCTAAAGGAG | 16 | 9 | 48 | 3 | LRRC59 |
| ATTAAGAGGG | 55 | 131 | 487 | 59 | MAN2C1 |
| CTGCACTTAC | 45 | 225 | 225 | 61 | MCM7 |
| GACCACCTTT | 8 | 36 | 126 | 35 | MFAP2 |
| GGTTGGCAGG | 12 | 36 | 116 | 27 | MFGE8 |
| TTGAAACTGT | 0 | 67 | 61 | 0 | MID1 |
| CAGGAGACCC | 0 | 12 | 124 | 7 | MMP11 |
| CTCTGTAAGT | 2 | 19 | 54 | 12 | MMP12 |
| TGTGTTGTCA | 6 | 41 | 51 | 14 | MTHFD2 |
| CAGGACCTGG | 0 | 28 | 51 | 12 | MYH7B |
| CGCAGCGGGT | 63 | 1 | 222 | 0 | NAPSA |
| GGACTTTCCT | 5 | 98 | 121 | 15 | NDRG1 |
| CAAGCCACAG | 0 | 52 | 64 | 5 | NDUFA4L2 |
| GAAAAATTTA | 66 | 259 | 277 | 88 | NGFRAP1 |
| AACCCTTGGG | 21 | 37 | 67 | 3 | NIPSNAP1 |
| GGTGGTACAC | 5 | 30 | 50 | 5 | NME4 |
| GGAAGTTCAA | 25 | 66 | 86 | 16 | NT5C3L |
| TTAAATTAAT | 2 | 51 | 48 | 0 | NTRK2 |
| GAAGATGTGG | 18 | 37 | 54 | 11 | NUCKS1 |
| ATGCAGCCAT | 28 | 146 | 128 | 36 | ODC1 |
| GTTAAATGCA | 9 | 51 | 58 | 2 | OXCT1 |
| GAGGAAGGCT | 13 | 33 | 42 | 14 | PGK1 |
| CGTGACCTGG | 0 | 112 | 45 | 3 | PHYHIP |
| TTAGTTTTTA | 4 | 57 | 88 | 11 | PLAT |
| GGAAGCTAAG | 6 | 8 | 101 | 2 | POSTN |
| CTGGGTGCCT | 0 | 527 | 172 | 3 | PSMB4 |
| CATCCTGCTG | 64 | 208 | 209 | 47 | PSMD2 |
| GGAGGTAGGG | 35 | 54 | 115 | 35 | PTK7 |
| GAATAAAGCA | 1 | 147 | 79 | 9 | PTPLB |
| AGGAGGGAGG | 15 | 40 | 58 | 18 | PYCR1 |
| TGTATACAAT | 9 | 44 | 42 | 0 | RAB6A |
| ATAATAAAGC | 22 | 66 | 250 | 81 | RARRES2 |
| CTGGGCGCCC | 0 | 169 | 91 | 6 | RHD |
| GAGAGGGCAG | 13 | 12 | 45 | 7 | RNF26 |
| CCTGAGGGTA | 14 | 18 | 44 | 9 | RPL8 |
| TGAAGTCACT | 13 | 56 | 90 | 30 | RPLP0 |
| AGCTCCCAGA | 9 | 15 | 58 | 7 | SEC62 |
| GACTCTTCAG | 15 | 57 | 661 | 12 | SERPINA3 |
| ATTTCTTCAA | 3 | 14 | 67 | 6 | SFRP2 |
| AATATTTTTA | 4 | 20 | 66 | 7 | SFRP2 |
| ATTGATGTGT | 34 | 9 | 116 | 11 | SFTPA2B |
| ATGGGATGGC | 47 | 0 | 173 | 0 | SFTPB |
| CACTCAAAGA | 6 | 0 | 86 | 0 | SFTPB |
| GCCGTGAACA | 0 | 0 | 129 | 6 | SFTPC |
| CTGCCAATAT | 36 | 0 | 120 | 0 | SFTPD |
| ATCACAGTGT | 14 | 42 | 85 | 28 | SHMT2 |
| GAGACTCCTG | 79 | 432 | 633 | 27 | SLC2A1 |
| GACCCACTAC | 6 | 30 | 74 | 11 | SLC3A2 |
| GAGACTGCAA | 28 | 39 | 98 | 18 | SLC40A1 |
| TCATTTTCCA | 27 | 443 | 139 | 7 | SLC6A8 |
| AGTGCTCACT | 3 | 118 | 94 | 11 | SLC6A8 |
| GAAATAAGGC | 2 | 246 | 77 | 0 | SLCO1A2 |
| GGTTGAAAAA | 11 | 317 | 348 | 15 | SNAR-E |
| CCCCCTCCGG | 11 | 30 | 78 | 15 | SNRPB |
| TTAGTGTCGT | 1 | 33 | 116 | 39 | SPARC |
| AATAGAAATT | 21 | 40 | 363 | 12 | SPP1 |
| ATATTAAATC | 3 | 21 | 53 | 3 | SRP68 |
| GTGATGTAAG | 22 | 123 | 295 | 11 | SRXN1 |
| TTCTTCTCGT | 9 | 24 | 59 | 3 | SUMO3 |
| ACAGGCTACG | 8 | 12 | 59 | 12 | TAGLN |
| GTGCAGGCTC | 20 | 18 | 60 | 0 | TAP1 |
| ACCTTTACTG | 40 | 115 | 353 | 80 | TFRC |
| GAAGTTTTTT | 17 | 76 | 56 | 12 | THOC3 |
| ACATTCCAAG | 4 | 12 | 74 | 21 | TIMP3 |
| TTATTTATGA | 10 | 9 | 55 | 8 | TIMP3 |
| ATGTGAAGAA | 24 | 59 | 98 | 21 | TMEM109 |
| ACCCGCCGGG | 69 | 249 | 612 | 84 | TNNC2 |
| ACTGTCTCCA | 9 | 50 | 50 | 6 | TPD52L1 |
| GTGCCCGTGC | 1 | 8 | 70 | 6 | TPI1 |
| GACCAGGCCC | 7 | 5 | 53 | 9 | TPM2 |
| AGTATGTATG | 11 | 49 | 110 | 7 | TRIB3 |
| GGCATCAGGG | 37 | 49 | 116 | 17 | TSKU |
| CCAACAAGAA | 4 | 68 | 169 | 17 | TSPAN7 |
| TAGATAATGG | 7 | 31 | 71 | 12 | TUBB |
| CTGGCGAGCG | 13 | 57 | 82 | 10 | UBE2S |
| CGTCTTTAAA | 0 | 12 | 50 | 10 | UBE2W |
| GAGATGAAAT | 5 | 11 | 43 | 8 | UCK2 |
| TCCAAATCGA | 21 | 14 | 121 | 0 | VIM |
| TAGATTCAAC | 13 | 41 | 46 | 12 | VKORC1L1 |
| GGAAAAATTA | 9 | 19 | 43 | 15 | ZNF207 |
| AGAGGTGTAG | 31 | 36 | 774 | 64 |  |
| GAAGTCGGAA | 22 | 69 | 625 | 29 |  |
| AACGAGGAAT | 70 | 182 | 619 | 66 |  |
| CTAACTAGTT | 75 | 183 | 531 | 96 |  |
| GGTCAGTCGG | 43 | 66 | 496 | 15 |  |
| GCCGTTCTTA | 94 | 214 | 472 | 62 |  |
| AATGGATGAA | 12 | 27 | 448 | 12 |  |
| GCTTTCTCAC | 78 | 247 | 330 | 106 |  |
| CCACGGGATT | 0 | 10 | 250 | 6 |  |
| AGAAGACGTT | 1 | 730 | 240 | 0 |  |
| CACCTCCTAT | 1 | 0 | 239 | 0 |  |
| CCGACGGGCG | 19 | 58 | 237 | 12 |  |
| AGTGCAGGGA | 1 | 692 | 194 | 29 |  |
| GCGTGCTCTC | 33 | 115 | 184 | 15 |  |
| GCTCCGAGCG | 54 | 171 | 178 | 15 |  |
| AAATAAAGCA | 1 | 392 | 174 | 0 |  |
| TGGCGTACGG | 16 | 40 | 144 | 20 |  |
| GGAAATAAAG | 3 | 309 | 134 | 2 |  |
| TCTAAGTACG | 24 | 46 | 120 | 11 |  |
| ATTTGAGAGT | 18 | 47 | 112 | 29 |  |
| AGGAAAGGTT | 1 | 0 | 102 | 0 |  |
| GAGATAAAGC | 0 | 250 | 78 | 3 |  |
| CAGGGGTGAC | 7 | 37 | 72 | 12 |  |
| GAAATAGAGC | 0 | 190 | 57 | 0 |  |
| CAACCAGTAA | 1 | 22 | 54 | 5 |  |
| TTGGCGGGTC | 13 | 29 | 51 | 12 |  |
| TGAACAGCAG | 2 | 4 | 48 | 0 |  |
| TGAATGTCAC | 2 | 50 | 46 | 0 |  |
| TAAATGTGCA | 4 | 79 | 46 | 9 |  |
| AGGGGAGCAC | 0 | 45 | 45 | 0 |  |
| TTTGTGTTAC | 2 | 3 | 45 | 7 |  |
| GAAGTAAAGC | 1 | 173 | 43 | 6 |  |
| CTAAGAAAGT | 2 | 1 | 43 | 0 |  |
| TTAGGTGATG | 0 | 34 | 42 | 0 |  |
| CAAGGTACAC | 1 | 6 | 41 | 0 |  |

^1^Tags with a three-fold or greater abundance in average normalized tag counts in SCC relative to both BE and PC; a minimal abundance of 40 TPM in SCC (232 tags in total).

^2^Average normalized tag counts expressed as tags per million (TPM) for 14 BE libraries.

^3^Average normalized tag counts (TPM) for five CIS libraries.

^4^Average normalized tag counts (TPM) for six SCC libraries.

^5^Average normalized tag counts (TPM) for two PC libraries.

^6^Tag-to-gene mapping according to SAGE Genie “Best Gene for Tag”, September 17, 2009 version. No entry is given for tags that map to transcript sequences within the databases of Full-Length Set, UniGene Consensus, or Unclustered ESTs.
