# Supplementary material for: Transcriptome Profiles of Carcinoma-in-Situ and Invasive Non-Small Cell Lung Cancer as Revealed by SAGE
Source: PLoS One. 2010 Feb 11;5(2):e9162. doi: 10.1371/journal.pone.0009162 (PMC2820080; doi:10.1371/journal.pone.0009162)
Supplement: Table S7 — Summary of real-time RT-PCR data for select genes within invasive cancer, bronchial epithelium, and lung parenchyma. (0.03 MB DOC) [file pone.0009162.s007.doc]

**Table S7**. Summary of real-time RT-PCR data for select genes within invasive cancer, bronchial epithelium, and lung parenchyma.

| **Gene** | **AvgFoldVsParenchyma ^1^** | **AvgFoldVsBE^2^** |
| --- | --- | --- |
| ARTN | 165.63 | 9.34 |
| C19ORF48 | 9.23 | 3.52 |
| CKS1B | 9.74 | 1.96 |
| CLDN1 | 40.12 | 0.81 |
| ECE2 | 23.03 | 4.83 |
| FBXO27 | 13.17 | 0.97 |
| MAGEA11 | 904696.33 | 106496.34 |
| MAGEA9 | 362.57 | 42.57 |
| MCM2 | 21.55 | 3.26 |
| NTS | 406.71 | 1.73 |
| POSTN | 42.62 | 21.42 |
| SFRP2 | 8.3 | 18.62 |
| SLC2A1-1 | 280.84 | 3.16 |
| SLC2A1-2 | 428.79 | 3.03 |
| SLC6A8 | 62.65 | 3.2 |
| UBE2S | 14.89 | 2 |

^1^The average of 9 pairs of tumor/ parenchyma.

^2^The average of 9 tumors versus the average of 6 bronchial epithelium brushings.
