# Supplementary material for: Transcriptome Profiles of Carcinoma-in-Situ and Invasive Non-Small Cell Lung Cancer as Revealed by SAGE
Source: PLoS One. 2010 Feb 11;5(2):e9162. doi: 10.1371/journal.pone.0009162 (PMC2820080; doi:10.1371/journal.pone.0009162)
Supplement: Table S8 — Input for GATHER analysis of gene ontology for genes differentially expressed in cancer datasets relative to BE and PC. (0.16 MB DOC) [file pone.0009162.s008.doc]

**Table S8. Input for GATHER analysis of gene ontology for genes differentially expressed in cancer datasets relative to BE and PC.**

| **Up-regulated** | | **Down-regulated** | |
| --- | --- | --- | --- |
| **CIS over BE_PC^1^** | **SCC over BE_PC^2^** | **BE_PC over CIS^3^** | **BE_PC over SCC^4^** |
| ABCC5 | A2M | AGR2 | ADH7 |
| AKR1B10 | ABCA4 | ANXA11 | AGR2 |
| AKR1C1 | ACTA1 | AQP3 | AQP5 |
| AKR1C3 | ADM | AQP5 | ATP5F1 |
| ALDOC | AKR1B10 | ASS1 | C15orf29 |
| APOC2 | AKR1C3 | ATP5F1 | FLJ20321 (CASZ1) |
| ARTN | ALDOC | C15orf29 | FLJ23754 (CCDC57) |
| ATP1B3 | ALG3 | C5A (C5AR1) | C6orf79 (CCDC90A) |
| ATP5G1 | ANTXR1 | C6orf79 (CCDC90A) | CCL4L1 |
| BEX2 | APOC2 | CD24 | CEACAM5 |
| BNIP2 | APOE | CEACAM6 | CRIP1 |
| C10orf99 | ARHGAP19 | CHRNA2 | CRIP2 |
| MGC13170 (C19orf48) | ARTN | CLDN3 | CTSW |
| FLJ31438 (C2orf63) | ATP5G1 | CLIC6 | CXCL3 |
| MGC13057 (C2orf88) | BGN | CLMN | DCLRE1C |
| C6orf15 | MGC13170 (C19orf48) | CRIP1 | DHCR24 |
| RAMA3 (CCDC34) | FLJ20519 (C1orf56) | CRIP2 | DHRS9 |
| CD27 | C1QC | CST6 | DUOX1 |
| CHST2 | C1R | CTSW | C1orf22 (EDEM3) |
| CHST3 | C1S | CXCL3 | MGC45840 (EFCAB4A) |
| CKS1B | C20orf24 | CYFIP1 | ENPP4 |
| C3orf4 (CLDND1) | C5orf13 | CYP27A1 | F3 |
| CLK2P | CALR | DHRS9 | FLJ31306 |
| CTAG1B | CCL20 | DUOX1 | LMO7 |
| CXCL14 | CCL26 | DUSP1 | LRRC16 (LRRC16A) |
| CYP26A1 | CCL5 | C1orf22 (EDEM3) | LYPDC2 (LYPD2) |
| DAPL1 | CDC25B | MGC45840 (EFCAB4A) | MUC4 |
| DERL3 | CDK4 | ERBB2 | MYO5C |
| DKK3 | CDKN2A | CVL (EZR) | NR4A2 |
| DSC2 | CHST2 | F3 | PDLIM4 |
| DSC3 | CKS1B | FAM3D | PLEKHA7 |
| DSG1 | CLDN1 | GNE | S100P |
| DST | CRR9 (CLPTM1L) | GP73 (GOLM1) | SCGB1A1 |
| ECE2 | COL12A1 | GPX1 | SERPINB11 |
| EFNA1 | COL17A1 | HNRNPM | SH3BGRL2 |
| EIF4EBP2 | COL1A1 | HSPA8 | SLC30A9 |
| ETNK2 | COL1A2 | KDELR2 | TJP3 |
| FADS2 | COL3A1 | KRT7 | OIP106 (TRAK1) |
| MGC33692 (FAM116B) | COL4A1 | LGALS3 | VILL |
| FAM20B | COL5A1 | LOC400986 |  |
| FAM43A | COL6A3 | LRRC16 (LRRC16A) |  |
| FASN | CPE | LYPDC2 (LYPD2) |  |
| FBL | CRP | MBD2 |  |
| FBXO27 | CRTAP | MLPH |  |
| FETUB | CST1 | MUC1 |  |
| KSP37 (FGFBP2) | CYR61 | MUC4 |  |
| FZD7 | DCN | MYO5C |  |
| G6PD | DDX49 | PLS1 |  |
| GLTP | DERL3 | PSCA |  |
| GPC3 | DKK3 | RAB20 |  |
| GPNMB | DUT | RNPC1 (RBM38) |  |
| GPX2 | ECE2 | S100P |  |
| GSTM1 | EFNA1 | SCGB1A1 |  |
| GSTM3 | EIF5A | SDC4 |  |
| GSTM4 | EPHB3 | SERPINA1 |  |
| HCG9 | ESM1 | SERPINB11 |  |
| IMP-3 (IGF2BP3) | FADS2 | SERPINB6 |  |
| IGHG1 | FAM43A | SH3BGRL2 |  |
| IGJ | FBL | CTL4 (SLC44A4) |  |
| IGKC | FBLN1 | SSBP4 |  |
| IGL@ | FBN1 | ST6GALNAC1 |  |
| IL23A | FBXO27 | TIARP (STEAP4) |  |
| ISG15 | FMOD | SUMF1 |  |
| KCNS3 | FN1 | TCEA3 |  |
| C1orf45 (KPRP) | FNDC3B | TJP3 |  |
| KRTDAP | FOXD4 | TMPRSS2 |  |
| LCE3D | FZD7 | TNFRSF14 |  |
| LEPREL1 | G6PD | OIP106 (TRAK1) |  |
| LOC284889 | GAA | TSPAN1 |  |
| LOC389904 | GDF15 | NET-7 (TSPAN15) |  |
| LY6K | GGH | A1S9 (UBA1) |  |
| MAGEA9 | GLIS2 | USP22 |  |
| MAP4K1 | GPC3 | VILL |  |
| MCM2 | GPNMB | WFDC2 |  |
| MCM7 | GPX2 |  |  |
| MID1 | GRIN2C |  |  |
| MIF | GSTM1 |  |  |
| MUC17 | GSTM3 |  |  |
| PBEF1 (NAMPT) | H19 |  |  |
| NDRG1 | HLA-G |  |  |
| NUOMS (NDUFA4L2) | ECGP (HSP90B1) |  |  |
| NGFRAP1 | IFI16 |  |  |
| NMD3 | IFI616 (IFI6) |  |  |
| AD24 (NOC3L) | IFITM1 |  |  |
| NOTCH3 | IGHG1 |  |  |
| NTRK2 | IGJ |  |  |
| NTS | IGKC |  |  |
| NXF4 | IGL@ |  |  |
| ODC1 | IL32 |  |  |
| OR5U1 (OR14J1) | IRF6 |  |  |
| OXCT1 | JUNB |  |  |
| PERP | KCNS3 |  |  |
| PHYHIP | LAPTM4B |  |  |
| PI3 | LDHA |  |  |
| PIM2 | LEPREL1 |  |  |
| PKP1 | LOC284889 |  |  |
| FLJ45651 (PLA2G4E) | LOC440731 |  |  |
| PLAT | LOC440995 |  |  |
| POLI | PRO1855 (LRRC59) |  |  |
| POU2AF1 | MAN2C1 |  |  |
| PPP2R1B | MCM7 |  |  |
| PSMB4 | MFAP2 |  |  |
| PSMD2 | MFGE8 |  |  |
| PTGDS | MID1 |  |  |
| PTPLB | MMP11 |  |  |
| RAB6A | MMP12 |  |  |
| RAD1 | MTHFD2 |  |  |
| RHD | MYH7B |  |  |
| RIMS3 | NAPSA |  |  |
| RNASE7 | NDRG1 |  |  |
| RRM2 | NUOMS (NDUFA4L2) |  |  |
| SERPINA3 | NGFRAP1 |  |  |
| SLC16A14 | NIPSNAP1 |  |  |
| SLC2A1 | NME4 |  |  |
| CTL1 (SLC44A1) | MGC20781 (NT5C3L) |  |  |
| SLC6A8 | NTRK2 |  |  |
| SLCO1A2 | NUCKS (NUCKS1) |  |  |
| SPAG4 | ODC1 |  |  |
| SPRR2E | OXCT1 |  |  |
| SPRR2F | PGK1 |  |  |
| SPRR2G | PHYHIP |  |  |
| C20orf139 (SRXN1) | PLAT |  |  |
| TESK1 | POSTN |  |  |
| THOC3 | PSMB4 |  |  |
| TKTL1 | PSMD2 |  |  |
| TNNC2 | PTK7 |  |  |
| TP63 | PTPLB |  |  |
| TPD52L1 | PYCR1 |  |  |
| TRIB3 | RAB6A |  |  |
| MXS1 (TSPAN7) | RARRES2 |  |  |
| TTBK1 | RHD |  |  |
| TTR | RNF26 |  |  |
| UBE2S | RPL8 |  |  |
| UTRN | RPLP0 |  |  |
| VKORC1L1 | SEC62 |  |  |
| ZNF277 | SERPINA3 |  |  |
| ZNF302 | SFRP2 |  |  |
| ZNF335 | SFTPA2B |  |  |
|  | SFTPB |  |  |
|  | SFTPC |  |  |
|  | SFTPD |  |  |
|  | SHMT2 |  |  |
|  | SLC2A1 |  |  |
|  | SLC3A2 |  |  |
|  | SLC40A1 |  |  |
|  | SLC6A8 |  |  |
|  | SLCO1A2 |  |  |
|  | SNRPB |  |  |
|  | SPARC |  |  |
|  | SPP1 |  |  |
|  | SRP68 |  |  |
|  | C20orf139 (SRNX1) |  |  |
|  | SUMO3 |  |  |
|  | TAGLN |  |  |
|  | TAP1 |  |  |
|  | TFRC |  |  |
|  | THOC3 |  |  |
|  | TIMP3 |  |  |
|  | MGC5508 (TMEM109) |  |  |
|  | TNNC2 |  |  |
|  | TPD52L1 |  |  |
|  | TPI1 |  |  |
|  | TPM2 |  |  |
|  | TRIB3 |  |  |
|  | TSK (TSKU) |  |  |
|  | MXS1 (TSPAN7) |  |  |
|  | TUBB |  |  |
|  | UBE2S |  |  |
|  | FLJ11011 (UBE2W) |  |  |
|  | UCK2 |  |  |
|  | VIM |  |  |
|  | VKORC1L1 |  |  |
|  | ZNF207 |  |  |

^1^Gene symbol, 138 genes analyzed; ^2^Gene symbol, 173 genes analyzed; ^3^Gene symbol, 74 genes analyzed; ^4^Gene symbol, 39 genes analyzed.

Gene symbols enclosed in brackets as cited by SAGE Genie tag-to-gene mapping, but not recognized by GATHER.
