# Supplementary material for: Transcriptome Profiles of Carcinoma-in-Situ and Invasive Non-Small Cell Lung Cancer as Revealed by SAGE
Source: PLoS One. 2010 Feb 11;5(2):e9162. doi: 10.1371/journal.pone.0009162 (PMC2820080; doi:10.1371/journal.pone.0009162)
Supplement: Table S9 — Down-regulated gene expression changes in carcinoma-in-situ relative to bronchial epithelium and precancerous lesions. (0.14 MB DOC) [file pone.0009162.s009.doc]

**Table S9. Down-regulated gene expression changes in carcinoma-in-situ relative to bronchial epithelium and precancerous lesions.**

| **Tag^1^** | **BE Mean^2^** | **CIS Mean^3^** | **SCC Mean^4^** | **PC Av^5^** | **Gene Symbol^6^** |
| --- | --- | --- | --- | --- | --- |
| ATTTTCTAAA | 1775 | 159 | 325 | 1017 | AGR2 |
| TGACTAATTG | 110 | 4 | 17 | 54 | AGR2 |
| GTGTCTCGCA | 64 | 8 | 28 | 86 | ANXA11 |
| TTTGCTTTTG | 517 | 66 | 207 | 1099 | AQP3 |
| CGTGGGGCTG | 319 | 37 | 85 | 317 | AQP5 |
| AACGTGCAGG | 49 | 8 | 31 | 110 | ASS1 |
| ATAAATTGGG | 56 | 19 | 19 | 64 | ATP5F1 |
| AAAAAAATCA | 197 | 65 | 61 | 215 | C15orf29 |
| ACTTTAATGA | 143 | 46 | 56 | 387 | C5AR1 |
| TATGTAAAAA | 141 | 17 | 14 | 58 | CCDC90A |
| GGAACAAACA | 1148 | 240 | 256 | 723 | CD24 |
| AAGGATAAAA | 209 | 14 | 37 | 56 | CEACAM6 |
| TGAATAAAGA | 46 | 15 | 24 | 267 | CHRNA2 |
| CTCGCGCTGG | 352 | 16 | 55 | 127 | CLDN3 |
| ATAAATGATT | 236 | 8 | 29 | 58 | CLIC6 |
| TGTATTCTTA | 142 | 4 | 21 | 47 | CLMN |
| TTTGGGCCTA | 986 | 51 | 135 | 398 | CRIP1 |
| TCCCTGGCAG | 99 | 5 | 28 | 94 | CRIP2 |
| GTGGAGGGCA | 61 | 14 | 19 | 43 | CST6 |
| GCAGCGGCAG | 1421 | 6 | 6 | 81 | CTSW |
| ATAATAAAAG | 479 | 110 | 78 | 403 | CXCL3 |
| CCTTTCTCTC | 245 | 44 | 144 | 764 | CYFIP1 |
| TCTCAGATGA | 41 | 1 | 26 | 42 | CYP27A1 |
| CTTATGGTCC | 200 | 32 | 50 | 167 | DHRS9 |
| GCAAGCCATT | 76 | 17 | 20 | 98 | DUOX1 |
| CTTGACATAC | 98 | 21 | 55 | 115 | DUSP1 |
| AAGAAAGCTC | 267 | 24 | 45 | 144 | EDEM3 |
| AAGGACCTAG | 62 | 7 | 11 | 58 | EFCAB4A |
| AGGAAGGAAC | 79 | 13 | 29 | 51 | ERBB2 |
| AAATAAAAGC | 1133 | 304 | 336 | 968 | EZR |
| TAGGAAAGTA | 55 | 15 | 17 | 65 | F3 |
| CCCCCGAAGC | 55 | 13 | 28 | 43 | FAM3D |
| TTAATTTATT | 45 | 12 | 29 | 68 | GNE |
| TTTAATTTGT | 192 | 22 | 44 | 64 | GOLM1 |
| CTCTTCGAGA | 125 | 26 | 118 | 162 | GPX1 |
| ACCCTTGGCC | 1047 | 102 | 510 | 510 | HNRNPM |
| CCAGGAGGAA | 352 | 116 | 183 | 345 | HSPA8 |
| GAAAGGTCTG | 80 | 26 | 62 | 88 | KDELR2 |
| CCTGGTCCCA | 172 | 41 | 121 | 272 | KRT7 |
| TTCACTGTGA | 1745 | 416 | 649 | 2461 | LGALS3 |
| TTCTAATCAT | 117 | 24 | 47 | 192 | LOC400986 |
| AGCTTAATGA | 1283 | 1 | 7 | 126 | LRRC16A |
| GCCCTATGCG | 165 | 42 | 18 | 165 | LYPD2 |
| TTTATTTTGA | 44 | 14 | 47 | 57 | MBD2 |
| AATGGAATGG | 239 | 35 | 46 | 113 | MLPH |
| CCTGGGAAGT | 441 | 102 | 208 | 460 | MUC1 |
| GATGGGTCCT | 61 | 8 | 14 | 53 | MUC4 |
| TCTGTTGAGT | 68 | 12 | 26 | 76 | MYLK4 |
| AAAAAATGAA | 117 | 11 | 21 | 66 | MYO5C |
| AGTGACAGAG | 192 | 14 | 9 | 50 | NWD1 |
| AAATCTGGCA | 86 | 13 | 20 | 48 | PLS1 |
| GCCCAGCATT | 214 | 14 | 67 | 179 | PSCA |
| TATTATTAAA | 77 | 12 | 31 | 158 | RAB20 |
| AGGTGAGAGG | 40 | 12 | 23 | 40 | RBM38 |
| TACCTCTGAT | 1120 | 293 | 250 | 1114 | S100P |
| CTTTGAGTCC | 42246 | 98 | 366 | 2547 | SCGB1A1 |
| TCCATCTGTT | 117 | 24 | 50 | 98 | SDC4 |
| GGAAAAGTGG | 129 | 33 | 159 | 136 | SERPINA1 |
| AAATACCTTA | 67 | 12 | 8 | 65 | SERPINB11 |
| ATGATGCGGT | 127 | 26 | 49 | 192 | SERPINB6 |
| TATGTATCCT | 79 | 15 | 8 | 50 | SH3BGRL2 |
| CACCTGTCAT | 417 | 15 | 46 | 60 | SLC44A4 |
| AGCGTGTGAT | 173 | 9 | 34 | 40 | SSBP4 |
| GAAAGGCAAA | 288 | 44 | 72 | 159 | ST6GALNAC1 |
| TTAAGGGATG | 166 | 24 | 30 | 73 | STEAP4 |
| CACTGTGTTG | 82 | 22 | 45 | 65 | SUMF1 |
| AACAAGGTGA | 66 | 15 | 24 | 60 | TCEA3 |
| ATGCGGAGTC | 83 | 11 | 15 | 64 | TJP3 |
| AAGGCCTTGT | 103 | 17 | 51 | 92 | TMEM219 |
| CAAATAAATT | 75 | 21 | 25 | 74 | TMPRSS2 |
| AAACAGTGTA | 111 | 22 | 58 | 135 | TNFRSF14 |
| TTATTGTATT | 100 | 19 | 27 | 149 | TRAK1 |
| GGAACTGTGA | 1023 | 40 | 248 | 307 | TSPAN1 |
| TTTTGTTTTG | 82 | 21 | 45 | 153 | TSPAN15 |
| CCAGCTGCCA | 46 | 13 | 44 | 45 | UBA1 |
| GCCTTTCCCT | 57 | 15 | 48 | 47 | USP22 |
| GCCACGTGGA | 65 | 1 | 20 | 95 | VILL |
| CCTGCTTGTC | 483 | 26 | 183 | 94 | WFDC2 |
| CACTACTCAC | 903 | 106 | 264 | 358 |  |
| AGTTAATAAA | 47 | 8 | 15 | 78 |  |
| CAGCTCCAAA | 53 | 12 | 10 | 55 |  |
| ATTTTTTCCT | 59 | 9 | 9 | 45 |  |
| TCTTTTTGTA | 197 | 2 | 9 | 42 |  |
| AAAGGTGATA | 70 | 11 | 26 | 40 |  |

^1^Tags with a three-fold or greater decrease in average normalized tag counts in CIS relative to both BE and PC; a minimal abundance of 40 TPM in both BE and PC (84 tags in total).

^2^Average normalized tag counts, expressed as tags per million (TPM) for 14 bronchial epithelial libraries.

^3^Average normalized tag counts (TPM) for five carcinoma-in-situ libraries.

^4^Average normalized tag counts (TPM) for six invasive cancer libraries.

^5^Average normalized tag counts (TPM) for two precancerous libraries.

^6^Tag-to-gene mapping according to SAGE Genie “Best Gene for Tag”, September 17, 2009 version. No entry is given for tags that map to transcript sequences within the databases of Full-Length Set, UniGene Consensus, or Unclustered ESTs.
