# Supplementary material for: Transcriptome Profiles of Carcinoma-in-Situ and Invasive Non-Small Cell Lung Cancer as Revealed by SAGE
Source: PLoS One. 2010 Feb 11;5(2):e9162. doi: 10.1371/journal.pone.0009162 (PMC2820080; doi:10.1371/journal.pone.0009162)
Supplement: Table S10 — Down-regulated gene expression changes in invasive cancer relative to bronchial epithelium and precancerous lesions. (0.09 MB DOC) [file pone.0009162.s010.doc]

**Table S10. Down-regulated gene expression changes in invasive cancer relative to bronchial epithelium and precancerous lesions.**

| **Tag^1^** | **BE Mean^2^** | **CIS Mean^3^** | **SCC Mean^4^** | **PC Av^5^** | **Gene Symbol^6^** |
| --- | --- | --- | --- | --- | --- |
| TTAAAAATTC | 305 | 248 | 77 | 318 | ADH7 |
| ATTTTCTAAA | 1775 | 159 | 325 | 1017 | AGR2 |
| TGACTAATTG | 110 | 4 | 17 | 54 | AGR2 |
| ATGGTGGGTG | 49 | 37 | 16 | 54 | ANKRD44 |
| CGTGGGGCTG | 319 | 37 | 85 | 317 | AQP5 |
| ATAAATTGGG | 56 | 19 | 19 | 64 | ATP5F1 |
| AAAAAAATCA | 197 | 65 | 61 | 215 | C15orf29 |
| ACTGATGCAA | 114 | 56 | 24 | 72 | CASZ1 |
| GTGCCTGTGC | 60 | 22 | 14 | 46 | CCDC57 |
| GTAAAAAAGC | 95 | 53 | 15 | 113 | CCDC64B |
| TATGTAAAAA | 141 | 17 | 14 | 58 | CCDC90A |
| TGTTTTCATA | 59 | 46 | 12 | 99 | CCL4L1 |
| AATATTTATA | 88 | 39 | 24 | 443 | CEACAM5 |
| TTTGGGCCTA | 986 | 51 | 135 | 398 | CRIP1 |
| TCCCTGGCAG | 99 | 5 | 28 | 94 | CRIP2 |
| GCAGCGGCAG | 1421 | 6 | 6 | 81 | CTSW |
| ATAATAAAAG | 479 | 110 | 78 | 403 | CXCL3 |
| ACTGTGCCAC | 54 | 26 | 16 | 95 | DCLRE1C |
| TCCCCGTGGC | 62 | 23 | 19 | 62 | DHCR24 |
| CTTATGGTCC | 200 | 32 | 50 | 167 | DHRS9 |
| GCAAGCCATT | 76 | 17 | 20 | 98 | DUOX1 |
| AAGAAAGCTC | 267 | 24 | 45 | 144 | EDEM3 |
| AAGGACCTAG | 62 | 7 | 11 | 58 | EFCAB4A |
| ATTTTTAAAA | 63 | 21 | 18 | 53 | ENPP4 |
| TAGGAAAGTA | 55 | 15 | 17 | 65 | F3 |
| GACTGAATGT | 49 | 51 | 13 | 56 | FLJ31306 |
| GTTTGTATAC | 155 | 56 | 48 | 213 | LMO7 |
| AGCTTAATGA | 1283 | 1 | 7 | 126 | LRRC16A |
| GCCCTATGCG | 165 | 42 | 18 | 165 | LYPD2 |
| GATGGGTCCT | 61 | 8 | 14 | 53 | MUC4 |
| AAAAAATGAA | 117 | 11 | 21 | 66 | MYO5C |
| TAAATGAAAA | 48 | 23 | 11 | 43 | NR4A2 |
| AGTGACAGAG | 192 | 14 | 9 | 50 | NWD1 |
| TGCAGTGACT | 67 | 17 | 13 | 44 | PDLIM4 |
| TTGCTTAATA | 43 | 20 | 5 | 45 | PLEKHA7 |
| TACCTCTGAT | 1120 | 293 | 250 | 1114 | S100P |
| CTTTGAGTCC | 42246 | 98 | 366 | 2547 | SCGB1A1 |
| AAATACCTTA | 67 | 12 | 8 | 65 | SERPINB11 |
| TATGTATCCT | 79 | 15 | 8 | 50 | SH3BGRL2 |
| TGGACCCAAC | 40 | 16 | 12 | 42 | SLC30A9 |
| ATGCGGAGTC | 83 | 11 | 15 | 64 | TJP3 |
| TTATTGTATT | 100 | 19 | 27 | 149 | TRAK1 |
| GCCACGTGGA | 65 | 1 | 20 | 95 | VILL |
| ACGTAACAGA | 64 | 32 | 20 | 96 |  |
| AGTTAATAAA | 47 | 8 | 15 | 78 |  |
| CAGCTCCAAA | 53 | 12 | 10 | 55 |  |
| ATTTTTTCCT | 59 | 9 | 9 | 45 |  |
| TCTTTTTGTA | 197 | 2 | 9 | 42 |  |

^1^Tags with a three-fold or greater decrease in average normalized tag counts in SCC relative to both BE and PC; a minimal abundance of 40 TPM in both BE and PC (48 tags in total).

^2^Average normalized tag counts, expressed as tags per million (TPM) for 14 bronchial epithelial libraries.

^3^Average normalized tag counts (TPM) for five carcinoma-in-situ libraries.

^4^Average normalized tag counts (TPM) for six invasive cancer libraries.

^5^Average normalized tag counts (TPM) for two precancerous libraries.

^6^Tag-to-gene mapping according to SAGE Genie “Best Gene for Tag”, September 17, 2009 version. No entry is given for tags that map to transcript sequences within the databases of Full-Length Set, UniGene Consensus, or Unclustered ESTs.
