# Supplementary material for: Transcriptome Profiles of Carcinoma-in-Situ and Invasive Non-Small Cell Lung Cancer as Revealed by SAGE
Source: PLoS One. 2010 Feb 11;5(2):e9162. doi: 10.1371/journal.pone.0009162 (PMC2820080; doi:10.1371/journal.pone.0009162)
Supplement: Table S11 — Microarray expression data for up-regulated genes with biomarker potential. (0.19 MB DOC) [file pone.0009162.s011.doc]

**Table S11.** **Microarray expression data for up-regulated genes with biomarker potential.**

| **Specimen^1^** | **Expression Value^2^ for COL3A1 (211161_s_at)** | **Expression Value^2^ for CST1 (206224_at)** | **Expression Value^2^ for GSTM3 (202554_s_at)** | **Expression Value^2^ for IGHG1 (211634_x_at)** | **Expression Value^2^ for KRTDAP (230835_at)** | **Expression Value^2^ for NTRK2 (221796_at)** | **Expression Value^2^ for SFTPC (211735_x_at)** | **Expression Value^2^ for SLCO1A2 (211481_at)** | **Expression Value^2^ for SPRR2G (236119_s_at)** |
| --- | --- | --- | --- | --- | --- | --- | --- | --- | --- |
| Squamous.1._.00-1059 | 10.3039405 | 4.872124646 | 11.74320752 | 5.436447936 | 4.824902427 | 3.221575764 | 7.192532171 | 5.162647387 | 5.851408467 |
| Squamous.10._.00-550 | 11.04838522 | 4.462159601 | 6.282919532 | 8.584783938 | 3.548200112 | 7.679205036 | 7.360655853 | 4.980140438 | 5.603672801 |
| Squamous.11._.00-827 | 11.87676118 | 7.013672279 | 6.482241718 | 7.370349352 | 3.443131625 | 3.886641603 | 12.60388469 | 5.205261851 | 5.879242878 |
| Squamous.12._.01-181 | 7.592012819 | 3.721405576 | 7.947523975 | 10.52541889 | 3.474105847 | 7.60535152 | 14.00427264 | 4.900330466 | 5.49946562 |
| Squamous.13._.01-189 | 11.3048605 | 3.539671243 | 9.473631729 | 6.246997468 | 7.963755543 | 8.603909919 | 11.05994788 | 4.961023431 | 6.879909526 |
| Squamous.14._.01-236 | 9.023933374 | 10.07821986 | 7.774985625 | 5.753873141 | 3.412211898 | 5.713400433 | 6.006482285 | 4.939076508 | 5.763242345 |
| Squamous.15._.01-284 | 11.54751952 | 7.393919278 | 8.133394823 | 7.367110212 | 7.966329778 | 6.769094371 | 6.118825682 | 4.727384552 | 8.761997523 |
| Squamous.16._.01-369 | 8.930343822 | 3.485565561 | 7.157726488 | 5.875926657 | 3.310122091 | 3.547611188 | 13.66163271 | 4.836360755 | 5.649849601 |
| Squamous.17._.01-424 | 11.94093595 | 4.328860103 | 6.673725842 | 7.58864841 | 5.765216651 | 8.158205186 | 11.91605087 | 5.078076459 | 7.411190101 |
| Squamous.18._.01-534 | 10.62264265 | 4.959910559 | 10.07627021 | 6.902862092 | 8.368600996 | 11.86044655 | 6.081357884 | 5.060306358 | 7.015766519 |
| Squamous.19._.01-758 | 7.9324224 | 4.588610697 | 10.40414044 | 9.776156762 | 9.248568982 | 8.908923225 | 6.128583776 | 5.145959423 | 6.64577879 |
| Squamous.2._.00-11 | 10.81285685 | 3.663647007 | 8.915863204 | 4.9137066 | 3.312556543 | 3.764264815 | 6.375197994 | 4.930901517 | 5.513779875 |
| Squamous.20._.10-00 | 10.37435454 | 7.515946381 | 7.136012031 | 8.249495426 | 3.996844176 | 3.661725424 | 6.594586407 | 5.436783735 | 6.057948793 |
| Squamous.21._.96-264 | 11.2035604 | 4.081007741 | 6.650161235 | 12.20552653 | 3.42440567 | 4.538072439 | 8.84050501 | 4.894777079 | 5.443970634 |
| Squamous.22._.96-475 | 10.84782474 | 4.249644826 | 11.18010964 | 6.119939541 | 3.304744938 | 9.855529508 | 6.888835345 | 4.715044841 | 5.779021686 |
| Squamous.23._.97-1026 | 11.02002343 | 3.88876048 | 6.611166119 | 7.942461081 | 3.680708541 | 3.484727058 | 13.38047164 | 5.079595045 | 6.050563117 |
| Squamous.24._.97-403 | 9.406751051 | 5.127586774 | 6.624283389 | 6.842351446 | 8.859066097 | 3.78548005 | 8.038803739 | 5.298747541 | 7.526792854 |
| Squamous.25._.97-587 | 12.49772633 | 5.863734486 | 8.640061241 | 9.305164674 | 6.218803591 | 4.497674109 | 8.530315263 | 4.834249208 | 6.224762373 |
| Squamous.26._.97-608 | 11.97766124 | 4.315950996 | 6.016144005 | 9.942323995 | 3.790315076 | 5.187415784 | 12.10585491 | 4.873055518 | 5.940976489 |
| Squamous.27._.97-646 | 10.82182453 | 3.912466214 | 6.159976236 | 8.512158985 | 3.958209106 | 3.378791063 | 8.4195998 | 5.019025793 | 6.057875168 |
| Squamous.28._.97-666 | 11.48252998 | 5.691649446 | 9.434594501 | 10.49074511 | 8.210682069 | 8.761621898 | 13.10248627 | 5.04048051 | 6.273161616 |
| Squamous.29._.97-792 | 11.39379206 | 3.92822171 | 10.4714422 | 7.864796443 | 6.757504149 | 11.8348307 | 12.68246114 | 5.252310971 | 5.795511042 |
| Squamous.3._.00-151 | 10.72972324 | 5.022316473 | 5.982740067 | 5.846014612 | 3.405831183 | 10.41640189 | 5.835451604 | 5.181585815 | 6.280569037 |
| Squamous.30._.97-829 | 10.90974516 | 6.903300568 | 8.837676656 | 9.459678073 | 7.472720305 | 5.457306498 | 6.26494924 | 4.961291647 | 6.65101662 |
| Squamous.31._.97-930 | 8.017573336 | 3.595148815 | 6.517216347 | 4.809267118 | 4.400395974 | 6.835358348 | 6.391775498 | 4.8903614 | 5.392437738 |
| Squamous.32._.97-949 | 10.02928798 | 5.459848392 | 7.239600493 | 10.27176492 | 5.482686784 | 7.624437146 | 12.63230077 | 4.876550845 | 5.891254279 |
| Squamous.33._.98-1277 | 8.474569185 | 3.813734463 | 12.07266961 | 7.213636675 | 3.932181063 | 7.771234027 | 10.64095959 | 4.89247781 | 5.848850687 |
| Squamous.34._.98-1293 | 10.67248948 | 5.721589339 | 12.1870872 | 8.644210089 | 5.916299987 | 10.47970429 | 11.44711344 | 5.120558922 | 6.512530458 |
| Squamous.35._.98-180 | 10.36416589 | 4.501397108 | 6.318516313 | 12.46923918 | 3.391059812 | 3.603140612 | 10.1358011 | 5.081947597 | 6.346692275 |
| Squamous.36._.98-292 | 8.283715406 | 3.723342234 | 6.061238451 | 7.875834579 | 3.782159666 | 11.1766371 | 10.01525726 | 4.961658016 | 5.77184648 |
| Squamous.37._.98-320 | 9.1132436 | 3.803738858 | 10.74416725 | 5.579114232 | 3.577632746 | 10.50369831 | 6.413893261 | 5.06738019 | 5.917651565 |
| Squamous.38._.98-375 | 11.67049028 | 4.033407691 | 8.093171286 | 7.331710732 | 3.508545963 | 6.729971348 | 5.913058495 | 4.937446921 | 5.750901021 |
| Squamous.39._.98-401 | 11.89462907 | 9.893045051 | 8.888451472 | 7.87854447 | 3.706233289 | 11.64328704 | 6.012936836 | 4.929511024 | 5.633592723 |
| Squamous.4._.00-315 | 8.416091026 | 6.450641232 | 10.33555623 | 9.149112817 | 3.510623454 | 11.2513591 | 11.73462378 | 5.212036332 | 6.027233008 |
| Squamous.40._.98-506 | 10.66285399 | 6.591743838 | 10.16371123 | 9.569485038 | 3.632770602 | 11.107829 | 6.047836636 | 4.757821583 | 5.940886824 |
| Squamous.41._.98-610 | 9.606639594 | 4.582968221 | 4.577895932 | 5.712324167 | 5.45122261 | 7.486441654 | 5.580217357 | 5.472426227 | 5.937360046 |
| Squamous.42._.98-828 | 10.38814129 | 4.084585947 | 11.14330697 | 9.06029398 | 3.297824801 | 9.627932071 | 5.868838203 | 5.11014212 | 6.963069912 |
| Squamous.43._.98-853 | 9.90287709 | 3.703650205 | 8.481454642 | 9.800517393 | 4.372709769 | 5.911515044 | 5.737698255 | 4.889728957 | 5.832516977 |
| Squamous.44._.98-933 | 10.41442372 | 6.592871596 | 9.290407493 | 7.222204995 | 4.00956109 | 10.03159434 | 9.764563275 | 4.965670341 | 5.640208562 |
| Squamous.45._.99-1067 | 8.562197871 | 3.664705029 | 7.314917903 | 4.80323177 | 3.434350151 | 7.626472092 | 13.3089981 | 4.96876813 | 6.131470682 |
| Squamous.46._.99-301 | 8.566570582 | 4.276240671 | 11.37231868 | 5.116369477 | 3.494347539 | 9.127586605 | 10.69079211 | 5.13696376 | 6.156506543 |
| Squamous.47._.99-625 | 9.577615778 | 4.050484565 | 8.503188275 | 6.202683038 | 3.330844901 | 10.8581339 | 6.671653214 | 5.130382002 | 5.66995877 |
| Squamous.48._.99-692 | 11.5059308 | 6.207247767 | 11.56795398 | 5.193442238 | 7.375945342 | 11.096851 | 6.075138625 | 5.1323094 | 8.694693158 |
| Squamous.49._.99-706 | 10.94812733 | 4.046993032 | 11.37566625 | 6.43981397 | 6.727248243 | 12.16814977 | 5.664514984 | 4.921575651 | 7.65745972 |
| Squamous.5._.00-327 | 12.09969208 | 3.467449704 | 7.756025882 | 9.878439632 | 3.965421797 | 6.450035791 | 13.42695474 | 5.097031389 | 6.086028665 |
| Squamous.50._.99-728 | 10.5899807 | 4.489535569 | 11.36888776 | 6.950469768 | 4.56510576 | 7.945441701 | 6.536368991 | 4.88902492 | 6.232123751 |
| Squamous.51._.99-77 | 11.54086907 | 4.596959528 | 7.469095243 | 7.316769978 | 9.740816328 | 9.705437999 | 6.168934261 | 4.944068298 | 8.253497963 |
| Squamous.52._.99-830 | 9.975778818 | 4.137110569 | 7.657085329 | 6.073716593 | 3.696199044 | 9.13196401 | 5.916930692 | 4.8903614 | 5.643224205 |
| Squamous.53._.99-927 | 10.55738305 | 4.008098078 | 6.103580539 | 6.727215056 | 3.814530217 | 9.632580849 | 5.876015037 | 5.449061878 | 5.983462082 |
| Squamous.6._.00-37 | 10.78888845 | 8.513254118 | 8.648472169 | 6.395429774 | 3.57450369 | 3.230212282 | 5.882645451 | 5.324129673 | 6.017016057 |
| Squamous.7._.00-440 | 11.74994287 | 4.158944609 | 9.391141177 | 10.42353443 | 3.478963074 | 3.202121765 | 5.95537163 | 4.881979897 | 5.692400018 |
| Squamous.8._.00-452 | 11.18695391 | 3.89939454 | 10.46170041 | 9.106533323 | 3.40830063 | 3.363320188 | 8.232020384 | 5.076332893 | 5.595706746 |
| Squamous.9._.00-502 | 10.22732001 | 5.650889813 | 8.14626925 | 10.84424995 | 3.932485078 | 11.56348633 | 10.80734926 | 5.293726372 | 6.207781314 |
| **Normal1** | 3.280194237 | 3.799217065 | 5.59261304 | 4.199017057 | 3.626282875 | 3.482236913 | 5.643332182 | 3.567679443 | 4.550824343 |
| **Normal2** | 3.149045046 | 3.577786071 | 5.562601221 | 4.403165804 | 3.37966529 | 3.25922716 | 5.646068767 | 3.467411211 | 4.584398316 |
| **Normal3** | 3.455647989 | 3.652448123 | 6.023157971 | 4.221660954 | 3.174947062 | 3.24135951 | 7.361585331 | 4.151785705 | 4.405095954 |
| **Normal4** | 3.109678235 | 3.297199987 | 5.321116325 | 4.748156051 | 2.995946361 | 3.157234122 | 6.344773572 | 4.12841687 | 4.852415528 |
| **Normal5** | 4.31421223 | 3.631232912 | 6.420363256 | 4.692079693 | 3.3910551 | 3.572810939 | 5.588114145 | 3.075692334 | 4.388738521 |
| **Normal6** | 3.862500974 | 3.60412071 | 5.447873997 | 4.759903083 | 3.184848836 | 3.271927839 | 5.677492006 | 3.528897181 | 4.45675129 |
| **Normal7** | 3.729741047 | 8.346057052 | 6.146130172 | 4.447662341 | 3.490355373 | 4.043934107 | 5.649787446 | 4.308920907 | 4.416842251 |
| **Normal8** | 2.937458466 | 3.595028549 | 5.264920496 | 4.838878876 | 3.22950534 | 2.947915083 | 5.821452562 | 4.192992795 | 4.784206333 |
| **Normal9** | 3.515145888 | 3.498217532 | 6.48010475 | 4.63865437 | 3.447617178 | 3.368577274 | 5.733809577 | 3.997841052 | 4.755370601 |
| **Normal10** | 3.547822476 | 3.869635047 | 5.608467498 | 4.323903502 | 3.604183488 | 3.612096605 | 5.932233497 | 4.38792372 | 4.664921292 |
| **Normal11** | 4.114663873 | 3.553117293 | 6.457099994 | 4.784361569 | 3.356198915 | 3.340271933 | 5.982882026 | 4.126575198 | 4.923219146 |
| **Normal12** | 5.076356946 | 3.33162822 | 7.85050277 | 4.316144501 | 3.371550895 | 3.197505064 | 5.842164232 | 4.631579807 | 4.860655141 |
| **Normal13** | 3.536176663 | 3.662141396 | 6.952892002 | 4.382111625 | 3.430305385 | 3.14716365 | 6.095948859 | 3.377549561 | 5.194223215 |
| **Normal14** | 3.094919019 | 3.644329012 | 5.792677758 | 4.844900707 | 3.316929944 | 3.38959351 | 5.943181745 | 4.704445436 | 4.938548958 |
| **Normal15** | 3.310687867 | 3.324553792 | 7.166795887 | 4.498173149 | 3.258327588 | 3.191237912 | 5.879073359 | 3.977611497 | 5.041936032 |
| **Normal16** | 4.316464395 | 3.948317927 | 6.669606966 | 4.906246541 | 3.506084517 | 3.281841668 | 5.820359531 | 3.450469795 | 4.391526303 |
| **Normal17** | 3.253109109 | 3.685494128 | 7.122050921 | 4.378126457 | 3.48698786 | 3.429159232 | 6.091527268 | 4.606682955 | 5.078900528 |
| **Normal18** | 3.233744725 | 4.281695695 | 6.881324375 | 4.633928913 | 3.640840927 | 3.604702492 | 6.099837754 | 3.507606292 | 4.898300875 |
| **Normal19** | 3.776633996 | 3.849513852 | 5.977529605 | 4.199012224 | 3.154322668 | 3.354985493 | 5.433498518 | 3.895727087 | 4.707794889 |
| **Normal20** | 4.161999849 | 3.874679159 | 5.707778779 | 4.394479829 | 3.512875391 | 3.351744034 | 5.327883183 | 4.29226824 | 4.459648111 |
| **Normal21** | 3.08020508 | 3.651422669 | 6.546616554 | 4.90098454 | 2.923049081 | 3.250056839 | 5.811524426 | 3.520859764 | 4.78436298 |
| **Normal22** | 4.090197962 | 3.498381449 | 5.898527503 | 4.292198936 | 3.305358723 | 3.400703553 | 5.833218522 | 4.006585835 | 4.574995727 |
| **Normal23** | 3.526414871 | 3.864587637 | 6.1189085 | 4.343043851 | 4.180322895 | 3.391293407 | 5.869458531 | 3.877419497 | 4.441639127 |
| **Normal24** | 4.128683168 | 4.334920684 | 6.003815308 | 5.135469956 | 3.65039867 | 3.607886455 | 5.677557823 | 3.483428405 | 4.501200672 |
| **Normal25** | 4.622663014 | 3.328419075 | 6.197110325 | 4.743936547 | 3.233783216 | 3.048501844 | 5.509096474 | 3.210891198 | 4.545174028 |
| **Normal26** | 3.609068828 | 3.327701312 | 6.125663325 | 4.695376324 | 3.521615912 | 3.161597717 | 5.476585418 | 4.471639546 | 4.937940323 |
| **Normal27** | 4.514016439 | 3.704570501 | 6.196941901 | 4.402477995 | 3.343430858 | 3.17724786 | 8.888575302 | 3.854087104 | 4.297420567 |
| **Normal28** | 3.435337204 | 3.529862329 | 5.708480005 | 4.455251102 | 3.67190048 | 3.573137758 | 5.949709491 | 3.528639922 | 4.566300021 |
| **Normal29** | 3.136881308 | 3.771921598 | 5.789775502 | 4.463017276 | 3.315727603 | 3.425741132 | 5.938256179 | 3.988557286 | 4.720190812 |
| **Normal30** | 3.074462357 | 3.235649443 | 5.552426511 | 4.427270654 | 3.163458175 | 3.346726098 | 5.554459861 | 3.949867575 | 4.739685918 |
| **Normal31** | 3.432040056 | 3.621392659 | 5.754607507 | 4.987269367 | 3.57732238 | 3.401086157 | 5.603850797 | 3.709609984 | 4.330706386 |
| **Normal32** | 3.403157339 | 3.906004026 | 6.490000832 | 4.837392639 | 3.140409886 | 3.740578121 | 5.785126499 | 4.463841179 | 4.456031621 |
| **Normal33** | 3.284587584 | 3.624680346 | 6.183278052 | 4.723024207 | 3.715635034 | 3.654153866 | 5.571810919 | 3.622191966 | 4.585690048 |
| **Normal34** | 3.593624993 | 3.867654339 | 5.791006044 | 4.764383698 | 3.063839019 | 3.340983593 | 5.84869966 | 4.133882603 | 4.710257902 |
| **Normal35** | 4.301204014 | 3.380572583 | 6.423377244 | 4.683175714 | 3.230246551 | 3.596881199 | 5.297644741 | 3.720235755 | 4.944198875 |
| **Normal36** | 3.449932687 | 3.600877853 | 6.412347781 | 5.271632612 | 3.389958106 | 3.500762084 | 5.596399717 | 4.528631331 | 5.0536951 |
| **Normal37** | 3.510583315 | 3.356644798 | 5.90873196 | 4.991639433 | 3.510675758 | 3.353839332 | 5.778384839 | 4.147382005 | 5.060525706 |
| **Normal38** | 3.615558136 | 4.525477165 | 5.743927264 | 4.91300131 | 3.632562792 | 4.155484543 | 5.599282688 | 3.906996782 | 5.523030104 |
| **Normal39** | 3.142425185 | 3.314573115 | 6.860792566 | 5.249643229 | 3.086044376 | 3.578082685 | 5.752211509 | 4.223907198 | 4.760917157 |
| **Normal40** | 3.314275292 | 3.588392864 | 6.046289182 | 4.484644478 | 3.367011288 | 3.322958104 | 5.665842974 | 3.502089585 | 5.039593943 |
| **Normal41** | 7.844414145 | 3.465498824 | 7.202460247 | 5.398668139 | 3.23090005 | 3.008326608 | 5.490651884 | 3.814186338 | 4.406224816 |
| **Normal42** | 3.313599053 | 3.357738923 | 5.340596549 | 4.843160062 | 3.27917104 | 3.325556942 | 6.25493513 | 3.918723824 | 4.883253889 |
| **Normal43** | 4.163293884 | 3.455642999 | 6.176923839 | 4.874488924 | 3.272437032 | 3.357269338 | 5.754227126 | 4.127050713 | 5.002089842 |
| **Normal44** | 3.296768384 | 3.351817287 | 5.94529203 | 5.115345813 | 3.397819053 | 3.34179552 | 5.762806253 | 3.958161978 | 4.934055723 |
| **Normal45** | 3.810252232 | 3.796087408 | 5.949140381 | 5.142774172 | 3.373787876 | 3.787043652 | 5.836786805 | 3.823311942 | 4.908390598 |
| **Normal46** | 3.734315852 | 3.486902911 | 6.202750856 | 4.834335757 | 3.389266322 | 3.172299912 | 5.647835106 | 4.241226544 | 5.310089452 |
| **Normal47** | 4.233447935 | 3.344769472 | 6.219046846 | 4.80447425 | 3.399391857 | 3.412592146 | 5.535464175 | 4.154104631 | 5.134145041 |
| **Normal48** | 3.582915625 | 3.390126833 | 5.937712826 | 4.63090727 | 3.182328538 | 3.11792884 | 5.487816302 | 4.329036742 | 4.995326397 |
| **Normal49** | 3.81422504 | 3.408596893 | 6.127551523 | 4.479717706 | 3.30279002 | 3.898939245 | 6.192779894 | 4.432154808 | 4.924374134 |
| **Normal50** | 3.741162491 | 3.895104691 | 6.16583432 | 4.992675564 | 3.38196117 | 3.533333898 | 6.089967712 | 4.264194257 | 4.951116138 |
| **Normal51** | 3.272970884 | 6.521272101 | 6.856696759 | 4.832121317 | 3.09303143 | 3.723781141 | 5.992279468 | 4.438231854 | 5.023993356 |
| **Normal52** | 4.413379506 | 3.721185949 | 5.34453009 | 4.667171464 | 3.173430444 | 3.466914446 | 5.109684469 | 4.295526 | 4.779932886 |
| **Normal53** | 3.529994169 | 3.664534109 | 5.832829936 | 4.786411254 | 3.159360971 | 3.600450103 | 5.734861294 | 4.389748238 | 4.934259174 |
| **Normal54** | 3.340992333 | 3.292224929 | 5.309147696 | 4.865869659 | 3.253295822 | 3.471085147 | 5.444012337 | 4.401676174 | 4.677766071 |
| **Normal55** | 3.854531503 | 3.476665293 | 5.640924569 | 4.854561314 | 3.394563938 | 3.31356189 | 5.280025327 | 4.697617268 | 4.823200749 |
| **Normal56** | 3.76263709 | 3.234181019 | 5.927660813 | 4.570687462 | 3.658605991 | 3.366817067 | 5.242641433 | 4.468514215 | 5.115109305 |
| **Normal57** | 3.687361455 | 3.505171 | 6.419146504 | 4.485581923 | 3.478621772 | 3.355035279 | 5.515568611 | 4.519497025 | 4.678998616 |
| **Normal58** | 4.011899379 | 3.563107137 | 5.864236333 | 4.74365619 | 3.523621846 | 3.408153817 | 5.686107393 | 4.705734942 | 5.003884411 |
| **Normal59** | 4.623495377 | 3.354489032 | 5.476299575 | 4.718308172 | 3.53445146 | 3.500578778 | 5.679589958 | 4.454422262 | 5.375063967 |
| **Normal60** | 3.798894567 | 3.3467042 | 6.58028734 | 4.940384322 | 3.34661775 | 3.717176437 | 5.657769628 | 4.684571767 | 4.861607385 |
| **Normal61** | 4.29177593 | 4.116560929 | 6.110067781 | 4.295796336 | 3.724020798 | 3.470456345 | 9.972306806 | 4.566038991 | 4.966447198 |
| **Normal62** | 3.879082131 | 3.541678027 | 6.09707042 | 5.267437871 | 3.548738282 | 3.41545561 | 6.119203377 | 4.41988682 | 5.173486271 |
| **Normal63** | 4.443504888 | 3.809805349 | 5.621826128 | 4.573183787 | 3.316208625 | 3.242656756 | 5.660728478 | 4.161707279 | 4.903948554 |
| **Normal64** | 3.383029939 | 3.543031723 | 5.805570977 | 4.595970705 | 3.517124194 | 3.214215421 | 6.793031705 | 4.236675072 | 4.968718432 |
| **Normal65** | 3.610944489 | 3.805899052 | 6.26466477 | 5.158206627 | 3.617403262 | 3.273351346 | 5.524704996 | 4.426735136 | 4.985540294 |
| **Normal66** | 3.131017662 | 3.900607399 | 6.325257418 | 4.271310726 | 3.330790196 | 3.165585917 | 5.528306002 | 4.350434214 | 5.29282537 |
| **Normal67** | 3.510794833 | 3.47263651 | 6.03062056 | 4.677189062 | 3.41968086 | 3.34134345 | 5.444860044 | 4.408970911 | 4.658777966 |

^1^Microarray expression data for 53 squamous tumors was downloaded from NCBI, GEO accession number GSE3141 (reference #35 main manuscript); 67 bronchial brushings (Normal1 – Normal67) were internally profiled.

^2^Expression value is log2 intensity, and was RMA normalized.
