# Supplementary material for: Transcriptome Profiles of Carcinoma-in-Situ and Invasive Non-Small Cell Lung Cancer as Revealed by SAGE
Source: PLoS One. 2010 Feb 11;5(2):e9162. doi: 10.1371/journal.pone.0009162 (PMC2820080; doi:10.1371/journal.pone.0009162)
Supplement: Table S12 — Up-regulated genes in CIS and SCC analyzed for frequent copy-number gain (and loss) in CIS specimens. (0.33 MB DOC) [file pone.0009162.s012.doc]

**Table S12. Up-regulated genes in CIS and SCC analyzed for frequent copy-number gain (and loss) in CIS specimens**

| **Upregulated Dataset^1^** | **Known Gene ID** | **RefSeq Accession Number** | **Gene Symbol^2^** | **Chromosome** | **Locus** | **txStart^3^** | **txEnd^4^** | **Gain Freq^5^** | **Loss Freq^5^** |
| --- | --- | --- | --- | --- | --- | --- | --- | --- | --- |
| CIS, SCC | uc001dyk.1 | NM_000561 | GSTM1 | 1 | 1p13 | 110031964 | 110037890 | 0.05 | 0.4 |
| CIS, SCC | uc001dyo.1 | NM_000849 | GSTM3 | 1 | 1p13 | 110078076 | 110084656 | 0.05 | 0.4 |
| CIS | uc001dyf.1 | NM_000850 | GSTM4 | 1 | 1p13 | 110000225 | 110005845 | 0.05 | 0.4 |
| CIS | uc001cfu.1 | NM_014747 | RIMS3 | 1 | 1p22 | 40858938 | 40903911 | 0.1 | 0.45 |
| SCC | uc001dqh.1 | NM_000350 | ABCA4 | 1 | 1p22.1 | 94230981 | 94359293 | 0.05 | 0.4 |
| SCC | uc001dle.1 | NM_001554 | CYR61 | 1 | 1p31 | 85819047 | 85821978 | 0.05 | 0.35 |
| CIS, SCC | uc001cks.1 | NM_057091 | ARTN | 1 | 1p33 | 44171578 | 44175471 | 0.1 | 0.35 |
| SCC | uc001bon.1 | NM_022873 | IFI6 | 1 | 1p35 | 27865158 | 27871311 | 0.1 | 0.4 |
| CIS, SCC | uc001cik.1 | NM_006516 | SLC2A1 | 1 | 1p35 | 43164105 | 43197088 | 0.1 | 0.35 |
| CIS | uc001acj.1 | NM_005101 | ISG15 | 1 | 1p36 | 938741 | 939776 | 0 | 0.65 |
| CIS, SCC | uc001bjz.1 | NM_016124 | RHD | 1 | 1p36 | 25471567 | 25529523 | 0.1 | 0.4 |
| SCC | uc001bga.1 | NM_172369 | C1QC | 1 | 1p36.11 | 22842712 | 22847190 | 0.1 | 0.45 |
| SCC | uc001azw.1 | NM_002403 | MFAP2 | 1 | 1p36.11 | 17173585 | 17179726 | 0.1 | 0.45 |
| SCC | uc001ftw.1 | NM_000567 | CRP | 1 | 1q21 | 157948702 | 157951003 | 0.4 | 0.1 |
| CIS, SCC | uc001fhh.1 | NM_004428 | EFNA1 | 1 | 1q21 | 153366972 | 153374010 | 0.35 | 0.1 |
| CIS | uc001han.1 | NM_018208 | ETNK2 | 1 | 1q21 | 202366813 | 202387645 | 0.4 | 0.1 |
| CIS | uc001fal.1 | NM_001025231 | KPRP | 1 | 1q21 | 150997129 | 151001153 | 0.35 | 0.1 |
| CIS | uc001fab.1 | NM_032563 | LCE3D | 1 | 1q21 | 150818483 | 150819604 | 0.35 | 0.1 |
| CIS, SCC | uc001eyb.1 |  | PSMB4 | 1 | 1q21 | 149638664 | 149641006 | 0.3 | 0.15 |
| CIS | uc001fbh.1 | NM_001024209 | SPRR2E | 1 | 1q21 | 151332234 | 151333625 | 0.35 | 0.1 |
| CIS | uc001fbi.1 | NM_001014450 | SPRR2F | 1 | 1q21 | 151351236 | 151352613 | 0.35 | 0.1 |
| CIS | uc001fbk.1 | NM_001014291 | SPRR2G | 1 | 1q21 | 151388681 | 151389231 | 0.35 | 0.1 |
| SCC | uc001ewm.1 | NM_017860 | C1orf56 | 1 | 1q21.2 | 149286873 | 149288250 | 0.3 | 0.1 |
| CIS, SCC | uc001fga.1 |  | CKS1B | 1 | 1q21.2 | 153213790 | 153218346 | 0.35 | 0.1 |
| SCC | uc001ftf.1 | NM_005531 | IFI16 | 1 | 1q22 | 157236381 | 157255065 | 0.4 | 0.1 |
| SCC | uc001gdp.1 | NM_012474 | UCK2 | 1 | 1q23 | 164063513 | 164143963 | 0.45 | 0.1 |
| CIS | uc001gmc.1 | NM_014864 | FAM20B | 1 | 1q25 | 177261696 | 177312321 | 0.4 | 0.1 |
| SCC | uc001gzg.1 | NM_002023 | FMOD | 1 | 1q32 | 201395247 | 201584028 | 0.4 | 0.1 |
| CIS | uc001gwd.1 | NM_000299 | PKP1 | 1 | 1q32 | 199519202 | 199568740 | 0.4 | 0.1 |
| SCC | uc001hdc.1 | NM_022731 | NUCKS1 | 1 | 1q32.1 | 203953731 | 203985916 | 0.4 | 0.1 |
| SCC | uc001hhq.1 | NM_006147 | IRF6 | 1 | 1q32.3 | 208027884 | 208046102 | 0.4 | 0.1 |
| SCC | uc001htm.1 | NM_001100 | ACTA1 | 1 | 1q42.13 | 227633615 | 227636466 | 0.4 | 0.1 |
| SCC | uc002sqh.1 | NM_198843 | SFTPB | 2 | 2p12 | 85737950 | 85748885 | 0.2 | 0.1 |
| SCC | uc002sfd.1 |  | ANTXR1 | 2 | 2p13.1 | 69093992 | 69184079 | 0.25 | 0.1 |
| SCC | uc002skj.1 | NM_001040409 | MTHFD2 | 2 | 2p13.1 | 74279197 | 74295932 | 0.2 | 0.1 |
| CIS, SCC | uc002rcv.1 | NM_002252 | KCNS3 | 2 | 2p24 | 17922594 | 17977706 | 0.3 | 0.1 |
| CIS, SCC | uc002ran.1 | NM_002539 | ODC1 | 2 | 2p25 | 10497958 | 10499521 | 0.3 | 0.05 |
| CIS | uc002rah.1 | NM_001034 | RRM2 | 2 | 2p25 | 10180185 | 10188074 | 0.3 | 0.05 |
| SCC | uc002uqj.1 | NM_000090 | COL3A1 | 2 | 2q31 | 189547343 | 189585717 | 0 | 0.2 |
| SCC | uc002uqp.1 | NM_014585 | SLC40A1 | 2 | 2q32 | 190133560 | 190153858 | 0 | 0.2 |
| SCC | uc002vpl.1 | NM_004591 | CCL20 | 2 | 2q33 | 228386813 | 228390494 | 0 | 0.35 |
| CIS, SCC | uc002uyw.1 | NM_003507 | FZD7 | 2 | 2q33 | 202607554 | 202611405 | 0.05 | 0.2 |
| SCC | uc002vez.1 |  | FN1 | 2 | 2q34 | 215933423 | 215965251 | 0 | 0.35 |
| CIS | uc002vqd.1 | NM_152527 | SLC16A14 | 2 | 2q36.3 | 230607941 | 230641863 | 0 | 0.3 |
| SCC | uc002vwj.1 |  | COL6A3 | 2 | 2q37 | 237897400 | 237916896 | 0.1 | 0.35 |
| SCC | uc003cfl.1 | NM_006371 | CRTAP | 3 | 3p22.3 | 33130482 | 33164269 | 0 | 0.8 |
| CIS | uc003dso.1 | NM_019895 | CLDND1 | 3 | 3q12.1 | 99708010 | 99722976 | 0.7 | 0.05 |
| SCC | uc003foz.1 | NM_004443 | EPHB3 | 3 | 3q21 | 185762280 | 185782890 | 0.9 | 0.05 |
| CIS | uc003ejp.1 | NM_004526 | MCM2 | 3 | 3q21 | 128799942 | 128823968 | 0.7 | 0.1 |
| CIS, SCC | uc003egj.1 | NM_198402 | PTPLB | 3 | 3q21.1 | 124693091 | 124786722 | 0.75 | 0.05 |
| CIS | uc003eug.1 | NM_001679 | ATP1B3 | 3 | 3q23 | 143078159 | 143128072 | 0.9 | 0.05 |
| CIS, SCC | uc003evm.1 | NM_004267 | CHST2 | 3 | 3q24 | 144321357 | 144324502 | 0.9 | 0.1 |
| CIS | uc003feb.1 | NM_015938 | NMD3 | 3 | 3q26.1 | 162421792 | 162452489 | 0.9 | 0.05 |
| SCC | uc003fhy.1 | NM_022763 | FNDC3B | 3 | 3q26.31 | 173240111 | 173601181 | 0.95 | 0.05 |
| CIS | uc003fmg.1 | NM_005688 | ABCC5 | 3 | 3q27 | 185120417 | 185218421 | 0.9 | 0.05 |
| CIS | uc003fqn.1 | NM_014375 | FETUB | 3 | 3q27 | 187840842 | 187853491 | 0.9 | 0.05 |
| SCC | uc003fne.1 | NM_005787 | ALG3 | 3 | 3q27.1 | 185442810 | 185449440 | 0.9 | 0.05 |
| CIS, SCC | uc003fni.1 | NM_014693 | ECE2 | 3 | 3q27.1 | 185450142 | 185493512 | 0.9 | 0.05 |
| CIS, SCC | uc003fnn.1 | NM_002808 | PSMD2 | 3 | 3q27.1 | 185499715 | 185509534 | 0.9 | 0.05 |
| SCC | uc003fsh.1 | NM_021101 | CLDN1 | 3 | 3q28 | 191506186 | 191522909 | 0.9 | 0.05 |
| CIS, SCC | uc003fsf.1 | NM_018192 | LEPREL1 | 3 | 3q28 | 191157315 | 191321412 | 0.9 | 0.05 |
| CIS, SCC | uc003fuj.1 | NM_153690 | FAM43A | 3 | 3q29 | 195887910 | 195891055 | 0.9 | 0.05 |
| SCC | uc003fvz.1 | NM_003234 | TFRC | 3 | 3q29 | 197260746 | 197293338 | 0.9 | 0.05 |
| CIS | uc003gon.1 | NM_031950 | FGFBP2 | 4 | 4p16 | 15570963 | 15573926 | 0 | 0.7 |
| CIS, SCC | uc003hfn.1 | NM_144646 | IGJ | 4 | 4q21 | 71740547 | 71751128 | 0.15 | 0.4 |
| SCC | uc003hra.1 | NM_001040058 | SPP1 | 4 | 4q21 | 89115825 | 89123587 | 0.2 | 0.35 |
| SCC | uc003inv.1 | NM_003013 | SFRP2 | 4 | 4q31.3 | 154921191 | 154929678 | 0.1 | 0.5 |
| SCC | uc003irg.1 | NM_001873 | CPE | 4 | 4q32.3 | 166519543 | 166638926 | 0.1 | 0.5 |
| CIS, SCC | uc003jmn.1 | NM_000436 | OXCT1 | 5 | 5p13.1 | 41765923 | 41906548 | 0.35 | 0.15 |
| CIS | uc003jiw.1 |  | RAD1 | 5 | 5p13.2 | 34941122 | 34951488 | 0.35 | 0.15 |
| SCC | uc003jcg.1 | NM_030782 | CLPTM1L | 5 | 5p15.3 | 1370999 | 1394975 | 0.5 | 0.15 |
| SCC | uc003jpk.1 | NM_007036 | ESM1 | 5 | 5q11.2 | 54309899 | 54317167 | 0 | 0.65 |
| SCC | uc003kpk.1 |  | C5orf13 | 5 | 5q22.1 | 111026218 | 111120854 | 0.1 | 0.45 |
| CIS | uc003lay.1 | NM_004887 | CXCL14 | 5 | 5q31 | 134934273 | 134942868 | 0 | 0.55 |
| SCC | uc003lug.1 | NM_003118 | SPARC | 5 | 5q31.3 | 151021201 | 151034428 | 0 | 0.65 |
| CIS, SCC | uc003mdg.1 | NM_032361 | THOC3 | 5 | 5q35.2 | 175319139 | 175327886 | 0 | 0.7 |
| CIS | uc003pcw.1 |  | DST | 6 | 6p12.1 | 56430752 | 56440233 | 0.1 | 0.25 |
| SCC | uc003oua.1 |  | PTK7 | 6 | 6p21.1 | 43152006 | 43209437 | 0.1 | 0.35 |
| CIS | uc003ouq.1 | NM_032538 | TTBK1 | 6 | 6p21.1 | 43319199 | 43363975 | 0.1 | 0.35 |
| CIS | uc003nsk.1 | NM_014070 | C6orf15 | 6 | 6p21.3 | 31186978 | 31188311 | 0.05 | 0.4 |
| CIS | uc003nos.1 | NM_005844 | HCG9 | 6 | 6p21.3 | 30050870 | 30054156 | 0.05 | 0.4 |
| SCC | uc003nnu.1 | NM_002127 | HLA-G | 6 | 6p21.3 | 29902722 | 29906774 | 0.05 | 0.4 |
| SCC | uc003ocg.1 | NM_000593 | TAP1 | 6 | 6p21.3 | 32920963 | 32929726 | 0.05 | 0.4 |
| SCC | uc003nrl.1 | NM_178014 | TUBB | 6 | 6p21.33 | 30796135 | 30801174 | 0.05 | 0.4 |
| SCC | uc003phs.1 | NM_004370 | COL12A1 | 6 | 6q12 | 75850761 | 75972343 | 0.05 | 0.4 |
| CIS, SCC | uc003pzu.1 | NM_003287 | TPD52L1 | 6 | 6q22 | 125516577 | 125626343 | 0.05 | 0.35 |
| CIS | uc003qht.1 | NM_022121 | PERP | 6 | 6q24 | 138453618 | 138470280 | 0 | 0.35 |
| CIS | uc003qkt.1 | NM_007124 | UTRN | 6 | 6q24 | 144654565 | 145215863 | 0 | 0.35 |
| CIS | uc003swg.1 | NM_006547 | IGF2BP3 | 7 | 7p11 | 23316352 | 23476520 | 0.15 | 0.15 |
| CIS, SCC | uc003swa.1 |  | GPNMB | 7 | 7p15 | 23252840 | 23264188 | 0.15 | 0.15 |
| CIS | uc003swk.1 |  | CLK2P | 7 | 7p15.3 | 23591036 | 23592682 | 0.15 | 0.15 |
| CIS, SCC | uc003tul.1 | NM_173517 | VKORC1L1 | 7 | 7q11.21 | 64975691 | 65057235 | 0.1 | 0.35 |
| SCC | uc003udt.1 | NM_006072 | CCL26 | 7 | 7q11.23 | 75236777 | 75257000 | 0.35 | 0.05 |
| CIS, SCC | uc003usv.1 | NM_182776 | MCM7 | 7 | 7q21.3 | 99528339 | 99536316 | 0.35 | 0.05 |
| SCC | uc003ung.1 | NM_000089 | COL1A2 | 7 | 7q22.1 | 93861808 | 93898480 | 0.3 | 0.05 |
| CIS | uc003uxp.1 | NM_001040105 | MUC17 | 7 | 7q22.1 | 100450083 | 100488860 | 0.45 | 0.05 |
| CIS | uc003vgc.1 |  | ZNF277 | 7 | 7q31.1 | 111633878 | 111716244 | 0.15 | 0.1 |
| CIS, SCC | uc003vrr.1 | NM_020299 | AKR1B10 | 7 | 7q33 | 133862938 | 133876700 | 0.15 | 0.15 |
| SCC | uc003wha.1 | NM_002889 | RARRES2 | 7 | 7q36.1 | 149666350 | 149669639 | 0.15 | 0.2 |
| CIS, SCC | uc003xos.1 | NM_000930 | PLAT | 8 | 8p12 | 42151909 | 42184351 | 0.6 | 0.05 |
| SCC | uc003xaw.1 |  | SFTPC | 8 | 8p21 | 22072178 | 22077928 | 0.1 | 0.5 |
| CIS, SCC | uc003xbj.1 | NM_014759 | PHYHIP | 8 | 8p21.3 | 22133180 | 22145549 | 0.1 | 0.5 |
| SCC | uc003xuw.1 | NM_003878 | GGH | 8 | 8q12.3 | 64090192 | 64113940 | 0.35 | 0.1 |
| SCC | uc003xzt.1 | NM_001001481 | UBE2W | 8 | 8q21.11 | 74854885 | 74905261 | 0.35 | 0.1 |
| SCC | uc003yia.1 | NM_018407 | LAPTM4B | 8 | 8q22.1 | 98856984 | 98934006 | 0.4 | 0.05 |
| CIS | uc003ywv.1 | NM_017527 | LY6K | 8 | 8q24.3 | 143778532 | 143782613 | 0.6 | 0.05 |
| CIS, SCC | uc003yuf.1 | NM_006096 | NDRG1 | 8 | 8q24.3 | 134318595 | 134337653 | 0.45 | 0.1 |
| SCC | uc003zdz.1 |  | RPL8 | 8 | 8q24.3 | 145985957 | 145988329 | 0.55 | 0.3 |
| CIS | uc003zwz.1 |  | TESK1 | 9 | 9p13 | 35595280 | 35599993 | 0.35 | 0.4 |
| SCC | uc003zxq.1 | NM_213674 | TPM2 | 9 | 9p13.2 | 35671989 | 35680053 | 0.3 | 0.5 |
| SCC | uc003zpj.1 | NM_058197 | CDKN2A | 9 | 9p21 | 21957750 | 21965038 | 0.05 | 0.85 |
| SCC | uc003zfz.1 | NM_207305 | FOXD4 | 9 | 9p24.3 | 106233 | 108417 | 0.05 | 0.65 |
| CIS, SCC | uc004anv.1 | NM_001007097 | NTRK2 | 9 | 9q22.1 | 86473285 | 86620441 | 0.15 | 0.45 |
| CIS | uc004bcn.1 | NM_080546 | SLC44A1 | 9 | 9q31.2 | 107046749 | 107193503 | 0.2 | 0.35 |
| SCC | uc004cfe.1 | NM_000093 | COL5A1 | 9 | 9q34.2 | 136673472 | 136876509 | 0.35 | 0.15 |
| CIS | uc004ckd.1 |  | PTGDS | 9 | 9q34.2 | 138989366 | 138996015 | 0.5 | 0.1 |
| SCC | uc001iou.1 | NM_003380 | VIM | 10 | 10p13 | 17310475 | 17319598 | 0 | 0.3 |
| CIS | uc001iho.1 | NM_001353 | AKR1C1 | 10 | 10p15 | 4924795 | 5010158 | 0.05 | 0.3 |
| CIS, SCC | uc001ihr.1 | NM_003739 | AKR1C3 | 10 | 10p15 | 4995453 | 5139878 | 0.05 | 0.3 |
| CIS | uc001jra.1 | NM_004096 | EIF4EBP2 | 10 | 10q21 | 71833927 | 71853676 | 0 | 0.25 |
| CIS | uc001jsn.1 | NM_004273 | CHST3 | 10 | 10q22.1 | 73394125 | 73443318 | 0 | 0.3 |
| SCC | uc001kbh.1 | NM_003019 | SFTPD | 10 | 10q22.2 | 81687475 | 81698841 | 0 | 0.2 |
| CIS | uc001kik.1 | NM_057157 | CYP26A1 | 10 | 10q23 | 94823221 | 94827631 | 0 | 0.2 |
| CIS | uc001kcu.1 | NM_207373 | C10orf99 | 10 | 10q23.1 | 85923533 | 85935030 | 0 | 0.25 |
| CIS | uc001kjq.1 | NM_022451 | NOC3L | 10 | 10q23.33 | 96082978 | 96112673 | 0 | 0.2 |
| SCC | uc001kmy.1 | NM_032900 | ARHGAP19 | 10 | 10q24.1 | 98902792 | 99042403 | 0.1 | 0.15 |
| SCC | uc001kxr.1 | NM_000494 | COL17A1 | 10 | 10q24.3 | 105781035 | 105835628 | 0.15 | 0.2 |
| CIS | uc001mrh.1 | NM_030771 | CCDC34 | 11 | 11p14.1 | 27316636 | 27341371 | 0 | 0.35 |
| CIS, SCC | uc001mju.1 | NM_001018057 | DKK3 | 11 | 11p15.2 | 11941118 | 11986762 | 0 | 0.45 |
| SCC | uc001mik.1 | NM_001124 | ADM | 11 | 11p15.4 | 10283206 | 10285499 | 0 | 0.45 |
| SCC | uc001mok.1 | NM_005566 | LDHA | 11 | 11p15.4 | 18372686 | 18385969 | 0.05 | 0.35 |
| SCC | uc001loy.1 | NM_003641 | IFITM1 | 11 | 11p15.5 | 303852 | 305272 | 0.35 | 0.15 |
| CIS, SCC | uc001nsj.1 |  | FADS2 | 11 | 11q12 | 61340324 | 61391401 | 0.15 | 0.15 |
| SCC | uc001nqg.1 | NM_024092 | TMEM109 | 11 | 11q12.2 | 60438252 | 60447491 | 0.1 | 0.15 |
| SCC | uc001nwb.1 | NM_001012661 | SLC3A2 | 11 | 11q13 | 62380093 | 62412929 | 0.15 | 0.15 |
| CIS, SCC | uc001oue.1 | NM_002869 | RAB6A | 11 | 11q13.3 | 73064330 | 73149849 | 0.05 | 0.25 |
| SCC | uc001oxt.1 | NM_015516 | TSKU | 11 | 11q13.5 | 76171932 | 76186846 | 0.05 | 0.25 |
| SCC | uc001phk.1 | NM_002426 | MMP12 | 11 | 11q22.3 | 102238673 | 102250922 | 0 | 0.25 |
| SCC | uc001pwh.1 | NM_032015 | RNF26 | 11 | 11q23 | 118710446 | 118713232 | 0.05 | 0.3 |
| CIS | uc001plg.1 | NM_006235 | POU2AF1 | 11 | 11q23.1 | 110728189 | 110755627 | 0 | 0.25 |
| CIS | uc001plw.1 | NM_181699 | PPP2R1B | 11 | 11q23.2 | 111102841 | 111142379 | 0 | 0.25 |
| SCC | uc001pql.1 |  | TAGLN | 11 | 11q23.2 | 116575249 | 116580713 | 0 | 0.3 |
| CIS, SCC | uc001rer.1 | NM_021094 | SLCO1A2 | 12 | 12p12 | 21311650 | 21379099 | 0 | 0.15 |
| SCC | uc001qsm.1 | NM_001733 | C1R | 12 | 12p13 | 7057769 | 7136184 | 0.05 | 0.1 |
| SCC | uc001qsj.1 | NM_201442 | C1S | 12 | 12p13 | 6966611 | 7048596 | 0.05 | 0.1 |
| CIS | uc001qod.1 | NM_001242 | CD27 | 12 | 12p13 | 6424311 | 6431145 | 0.05 | 0.05 |
| SCC | uc001qrk.1 | NM_000365 | TPI1 | 12 | 12p13 | 6846966 | 6850253 | 0.05 | 0.05 |
| SCC | uc001qvj.1 | NM_000014 | A2M | 12 | 12p13.3 | 9111570 | 9123691 | 0.05 | 0.15 |
| SCC | uc001snf.1 | NM_005412 | SHMT2 | 12 | 12q12 | 55909818 | 55914981 | 0 | 0.15 |
| CIS | uc001sla.1 | NM_016584 | IL23A | 12 | 12q13.2 | 55018929 | 55020461 | 0 | 0.15 |
| CIS, SCC | uc001snm.1 |  | NDUFA4L2 | 12 | 12q13.3 | 55914952 | 55917202 | 0 | 0.15 |
| SCC | uc001spv.1 | NM_000075 | CDK4 | 12 | 12q14 | 56428269 | 56432431 | 0 | 0.15 |
| CIS | uc001tag.1 | NM_006183 | NTS | 12 | 12q21 | 84792205 | 84800896 | 0 | 0.15 |
| SCC | uc001tbo.1 | NM_133504 | DCN | 12 | 12q21.33 | 90063165 | 90096493 | 0.45 | 0.1 |
| CIS | uc001tpm.1 | NM_016433 | GLTP | 12 | 12q24.11 | 108773130 | 108802676 | 0 | 0.2 |
| SCC | uc001tkb.1 | NM_003299 | HSP90B1 | 12 | 12q24.2 | 102848318 | 102865833 | 0 | 0.25 |
| SCC | uc002rqt.1 | NM_053275 | RPLP0 | 12 | 12q24.2 | 38562429 | 38563529 | 0.25 | 0.05 |
| SCC | uc001uwo.1 | NM_006475 | POSTN | 13 | 13q13.3 | 37034778 | 37070874 | 0.1 | 0.8 |
| SCC | uc001vqw.1 | NM_001845 | COL4A1 | 13 | 13q34 | 109599310 | 109757459 | 0.05 | 0.7 |
| CIS | uc001vzk.1 | NM_032572 | RNASE7 | 14 | 14q11.2 | 20580250 | 20582226 | 0 | 0.35 |
| CIS, SCC | uc001xhx.1 | NM_002083 | GPX2 | 14 | 14q24.1 | 64475624 | 64479284 | 0.05 | 0.35 |
| CIS, SCC | uc001ydo.1 | NM_001085 | SERPINA3 | 14 | 14q32.1 | 94128146 | 94160143 | 0.05 | 0.35 |
| CIS, SCC | uc001yse.1 |  | IGHG1 | 14 | 14q32.33 | 105273724 | 105401515 | 0 | 0.85 |
| SCC | uc002baf.1 | NM_006715 | MAN2C1 | 15 | 15q11 | 73435185 | 73447994 | 0.2 | 0.25 |
| SCC | uc001zws.1 | NM_001025248 | DUT | 15 | 15q15 | 46410912 | 46422862 | 0.1 | 0.3 |
| CIS | uc001zov.1 | NM_001080490 | PLA2G4E | 15 | 15q15.1 | 40063243 | 40077012 | 0.2 | 0.2 |
| SCC | uc001zwx.1 | NM_000138 | FBN1 | 15 | 15q21.1 | 46487796 | 46725210 | 0.1 | 0.25 |
| CIS | uc002agh.1 | NM_004330 | BNIP2 | 15 | 15q22.2 | 57742355 | 57768778 | 0.2 | 0.2 |
| SCC | uc002bnf.1 | NM_005928 | MFGE8 | 15 | 15q25 | 87242919 | 87256759 | 0.15 | 0.3 |
| SCC | uc002cwc.1 | NM_032575 | GLIS2 | 16 | 16p13.3 | 4322216 | 4329599 | 0.1 | 0.15 |
| SCC | uc002ctk.1 | NM_001012633 | IL32 | 16 | 16p13.3 | 3055313 | 3059669 | 0.35 | 0.1 |
| SCC | uc002cgy.1 | NM_005009 | NME4 | 16 | 16p13.3 | 386725 | 390755 | 0.65 | 0.05 |
| SCC | uc002gfr.1 |  | EIF5A | 17 | 17p13 | 7151041 | 7156152 | 0.05 | 0.65 |
| SCC | uc002hkf.1 | NM_002985 | CCL5 | 17 | 17q11.2 | 31222608 | 31231490 | 0.25 | 0.1 |
| SCC | uc002hhh.1 | NM_003457 | ZNF207 | 17 | 17q11.2 | 27701269 | 27721583 | 0.35 | 0.1 |
| CIS, SCC | uc002hbp.1 | NM_005165 | ALDOC | 17 | 17q12 | 23924259 | 23928078 | 0.35 | 0.1 |
| SCC | uc002hxx.1 | NM_052935 | NT5C3L | 17 | 17q21.2 | 37234983 | 37242266 | 0.25 | 0.15 |
| CIS, SCC | uc002iog.1 | NM_005175 | ATP5G1 | 17 | 17q21.32 | 44325146 | 44328231 | 0.45 | 0.05 |
| SCC | uc002iqm.1 | NM_000088 | COL1A1 | 17 | 17q21.33 | 45616455 | 45633999 | 0.35 | 0.05 |
| SCC | uc002iqt.1 | NM_018509 | LRRC59 | 17 | 17q21.33 | 45813597 | 45829831 | 0.35 | 0.05 |
| CIS | uc002kdu.1 | NM_004104 | FASN | 17 | 17q25 | 77629502 | 77649395 | 0.65 | 0.15 |
| SCC | uc002jlt.1 | NM_000835 | GRIN2C | 17 | 17q25 | 70349762 | 70367602 | 0.45 | 0.05 |
| SCC | uc002jqj.1 | NM_014230 | SRP68 | 17 | 17q25.1 | 71546785 | 71561332 | 0.6 | 0.05 |
| SCC | uc002jxo.1 | NM_001079803 | GAA | 17 | 17q25.2 | 75689949 | 75708274 | 0.45 | 0.2 |
| SCC | uc002kcp.1 | NM_153824 | PYCR1 | 17 | 17q25.3 | 77483557 | 77488259 | 0.6 | 0.15 |
| CIS | uc002kwk.1 | NM_004949 | DSC2 | 18 | 18q12.1 | 26900003 | 26936375 | 0.2 | 0.2 |
| CIS | uc002kwi.1 | NM_024423 | DSC3 | 18 | 18q12.1 | 26825028 | 26876687 | 0.2 | 0.2 |
| CIS | uc002kwp.1 | NM_001942 | DSG1 | 18 | 18q12.1 | 27152049 | 27191391 | 0.2 | 0.2 |
| CIS | uc002kwx.1 | NM_000371 | TTR | 18 | 18q12.1 | 27425837 | 27432781 | 0.2 | 0.2 |
| CIS | uc002lfj.1 | NM_007195 | POLI | 18 | 18q21.1 | 50049922 | 50075092 | 0.1 | 0.35 |
| SCC | uc002nkq.1 | NM_019070 | DDX49 | 19 | 19p12 | 18891493 | 18900436 | 0.55 | 0.1 |
| SCC | uc002niv.1 | NM_004864 | GDF15 | 19 | 19p13.11 | 18357984 | 18360986 | 0.45 | 0.15 |
| SCC | uc002mvb.1 | NM_002229 | JUNB | 19 | 19p13.2 | 12763285 | 12766248 | 0.35 | 0.2 |
| CIS | uc002nan.1 | NM_000435 | NOTCH3 | 19 | 19p13.2 | 15131443 | 15172792 | 0.35 | 0.15 |
| SCC | uc002mvu.1 | NM_004343 | CALR | 19 | 19p13.3 | 12910422 | 12916303 | 0.35 | 0.2 |
| CIS, SCC | uc002omm.1 | NM_001436 | FBL | 19 | 19q13.1 | 45016937 | 45022995 | 0.4 | 0.1 |
| CIS | uc002oiw.1 | NM_001042600 | MAP4K1 | 19 | 19q13.1 | 43770120 | 43790441 | 0.35 | 0.25 |
| CIS | uc002nvp.1 | NM_018443 | ZNF302 | 19 | 19q13.11 | 39860406 | 39869142 | 0.45 | 0.15 |
| CIS, SCC | uc002pah.1 | NM_000483 | APOC2 | 19 | 19q13.2 | 50141082 | 50144658 | 0.2 | 0.3 |
| SCC | uc002pab.1 | NM_000041 | APOE | 19 | 19q13.2 | 50100878 | 50104490 | 0.2 | 0.3 |
| CIS, SCC | uc002okh.1 | NM_178820 | FBXO27 | 19 | 19q13.2 | 44206502 | 44215038 | 0.4 | 0.1 |
| CIS, SCC | uc002pte.1 |  | C19orf48 | 19 | 19q13.33 | 55992772 | 55998944 | 0.15 | 0.3 |
| SCC | uc002prx.1 | NM_004851 | NAPSA | 19 | 19q13.33 | 55553545 | 55560743 | 0.25 | 0.25 |
| CIS, SCC | uc002qkx.1 | NM_014501 | UBE2S | 19 | 19q13.43 | 60604461 | 60611137 | 0.05 | 0.45 |
| SCC | uc002wtp.1 | NM_001898 | CST1 | 20 | 20p11.21 | 23676189 | 23679574 | 0.2 | 0.15 |
| SCC | uc002wjl.1 | NM_021873 | CDC25B | 20 | 20p13 | 3724385 | 3734761 | 0.3 | 0.05 |
| SCC | uc002wfz.1 | NM_198216 | SNRPB | 20 | 20p13 | 2390280 | 2399499 | 0.3 | 0.15 |
| CIS, SCC | uc002wea.1 | NM_080725 | SRXN1 | 20 | 20p13 | 575267 | 581890 | 0.25 | 0.15 |
| CIS, SCC | uc002wdm.1 | NM_021158 | TRIB3 | 20 | 20p13 | 309307 | 326203 | 0.15 | 0.2 |
| CIS | uc002xdb.1 | NM_003116 | SPAG4 | 20 | 20q11.21 | 33667222 | 33672379 | 0.35 | 0.05 |
| CIS | uc002xqv.1 | NM_022095 | ZNF335 | 20 | 20q11.21 | 44010698 | 44016750 | 0.3 | 0.05 |
| SCC | uc002xbi.1 | NM_020884 | MYH7B | 20 | 20q11.22 | 33026866 | 33053897 | 0.35 | 0.05 |
| SCC | uc002xfp.1 | NM_018840 | C20orf24 | 20 | 20q11.23 | 34667580 | 34674354 | 0.35 | 0.05 |
| CIS | uc002xng.1 | NM_002638 | PI3 | 20 | 20q12 | 43236911 | 43238599 | 0.25 | 0.05 |
| CIS, SCC | uc002xpr.1 | NM_003279 | TNNC2 | 20 | 20q12 | 43885261 | 43889360 | 0.25 | 0.05 |
| SCC | uc002zfz.1 | NM_006936 | SUMO3 | 21 | 21q22.3 | 45049959 | 45062472 | 0.35 | 0.35 |
| CIS, SCC | uc002zws.1 |  | IGL@ | 22 | 22q11.1 | 21559959 | 21578969 | 0.25 | 0.2 |
| SCC | uc002zxx.1 | NM_005940 | MMP11 | 22 | 22q11.2 | 22445035 | 22456503 | 0.3 | 0.2 |
| CIS, SCC | uc002zyh.1 | NM_001002862 | DERL3 | 22 | 22q11.23 | 22506689 | 22511201 | 0.3 | 0.15 |
| CIS | uc002zyq.1 |  | MIF | 22 | 22q11.23 | 22566564 | 22567405 | 0.3 | 0.15 |
| SCC | uc003ana.1 |  | TIMP3 | 22 | 22q12.1 | 31526801 | 31586540 | 0.25 | 0.2 |
| SCC | uc003afx.1 | NM_003634 | NIPSNAP1 | 22 | 22q12.2 | 28280799 | 28307326 | 0.25 | 0.15 |
| SCC | uc003bgg.1 | NM_006487 | FBLN1 | 22 | 22q13.31 | 44277382 | 44333255 | 0.3 | 0.15 |
| CIS | uc003bkx.1 | NM_001001794 | FAM116B | 22 | 22q13.33 | 49092963 | 49100010 | 0.65 | 0.1 |

^1^Relative to BE and PC.

^2^Sorted according to chromosomal locus.

^3^Transcription start.

^4^Transcription end.

^5^Average frequency from 20 independent CIS specimens.
