# Supplementary material for: Transcriptome Profiles of Carcinoma-in-Situ and Invasive Non-Small Cell Lung Cancer as Revealed by SAGE
Source: PLoS One. 2010 Feb 11;5(2):e9162. doi: 10.1371/journal.pone.0009162 (PMC2820080; doi:10.1371/journal.pone.0009162)
Supplement: Table S13 — Down-regulated genes in CIS and SCC analyzed for frequent copy-number loss (and gain) in CIS specimens. (0.14 MB DOC) [file pone.0009162.s013.doc]

**Table S13.** **Down-regulated genes in CIS and SCC analyzed for frequent copy-number loss (and gain) in CIS specimens**

| **Down-regulated Dataset^1^** | **Known Gene ID** | **RefSeq Accession Number** | **Gene Symbol^2^** | **Chromosome** | **Locus** | **txStart^3^** | **txEnd^4^** | **GainFreq^5^** | **LossFreq^5^** |
| --- | --- | --- | --- | --- | --- | --- | --- | --- | --- |
| CIS, SCC | uc001ebc.1 | NM_001688 | ATP5F1 | 1 | 1p13.2 | 111793265 | 111806048 | 0.05 | 0.4 |
| CIS, SCC | uc001dqp.1 |  | F3 | 1 | 1p22 | 94767460 | 94779210 | 0.05 | 0.4 |
| SCC | uc001cyc.1 | NM_014762 | DHCR24 | 1 | 1p33 | 55087887 | 55125509 | 0.1 | 0.35 |
| CIS | uc001cpd.1 | NM_005727 | TSPAN1 | 1 | 1p34.1 | 46418798 | 46424217 | 0.15 | 0.3 |
| CIS | uc001bgx.1 | NM_003196 | TCEA3 | 1 | 1p36.12 | 23580141 | 23623848 | 0.1 | 0.4 |
| SCC | uc001aro.1 | NM_001079843 | CASZ1 | 1 | 1p36.22 | 10619252 | 10779294 | 0.25 | 0.4 |
| CIS | uc001ajr.1 | NM_003820 | TNFRSF14 | 1 | 1p36.3 | 2479150 | 2486613 | 0.5 | 0.25 |
| CIS | uc001fhy.1 | NM_002456 | MUC1 | 1 | 1q21 | 153424923 | 153427775 | 0.35 | 0.1 |
| CIS, SCC | uc001gqx.1 | NM_025191 | EDEM3 | 1 | 1q24-25 | 182926259 | 182990327 | 0.4 | 0.1 |
| SCC | uc002tyx.1 | NM_173173 | NR4A2 | 2 | 2q22-23 | 156889194 | 156897446 | 0.05 | 0.15 |
| CIS, SCC | uc002uep.1 | NM_005771 | DHRS9 | 2 | 2q31.1 | 169629544 | 169660923 | 0.05 | 0.15 |
| CIS | uc002viz.1 | NM_000784 | CYP27A1 | 2 | 2q33 | 219354948 | 219388259 | 0 | 0.3 |
| SCC | uc002utz.1 | NM_153697 | ANKRD44 | 2 | 2q33.1 | 197539985 | 197695761 | 0 | 0.2 |
| CIS | uc002vws.1 | NM_001042467 | MLPH | 2 | 2q37.3 | 238059791 | 238128700 | 0.1 | 0.35 |
| CIS | uc003dkq.1 | NM_138805 | FAM3D | 3 | 3p14.2 | 58594709 | 58627601 | 0.1 | 0.7 |
| CIS | uc003cws.1 | NM_000581 | GPX1 | 3 | 3p21.3 | 49369612 | 49370795 | 0 | 0.6 |
| CIS, SCC | uc003chj.1 | NM_015873 | VILL | 3 | 3p21.3 | 38004533 | 38023680 | 0 | 0.8 |
| CIS, SCC | uc003cky.1 | NM_001042646 | TRAK1 | 3 | 3p25.3 | 42107749 | 42242272 | 0 | 0.8 |
| CIS | uc003bpz.1 | NM_182760 | SUMF1 | 3 | 3p26.2 | 4377829 | 4483954 | 0.05 | 0.8 |
| CIS | uc003euz.1 | NM_002670 | PLS1 | 3 | 3q23 | 143825033 | 143915191 | 0.95 | 0.05 |
| CIS, SCC | uc003fuz.1 |  | MUC4 | 3 | 3q29 | 196959308 | 196986810 | 0.9 | 0.05 |
| SCC | uc003gwl.1 | NM_006345 | SLC30A9 | 4 | 4p13 | 41687279 | 41784308 | 0 | 0.7 |
| CIS, SCC | uc003gjl.1 | NM_005980 | S100P | 4 | 4p16 | 6746466 | 6749798 | 0.05 | 0.6 |
| CIS, SCC | uc003hhl.1 | NM_002090 | CXCL3 | 4 | 4q21 | 75121175 | 75123354 | 0.25 | 0.35 |
| SCC | uc003huv.1 | NM_000673 | ADH7 | 4 | 4q23 | 100552440 | 100575548 | 0.2 | 0.35 |
| SCC | uc003kwn.1 | NM_003687 | PDLIM4 | 5 | 5q31.1 | 131621285 | 131637046 | 0 | 0.55 |
| CIS | uc003mbu.1 | NM_004417 | DUSP1 | 5 | 5q34 | 172127706 | 172130240 | 0 | 0.65 |
| SCC | uc003oxy.1 | NM_014936 | ENPP4 | 6 | 6p12.3 | 46205870 | 46222237 | 0.15 | 0.25 |
| CIS | uc003nxt.1 | NM_025257 | SLC44A4 | 6 | 6p21.3 | 31938953 | 31954753 | 0.05 | 0.4 |
| CIS, SCC | uc003nbd.1 | NM_001031713 | CCDC90A | 6 | 6p24.3 | 13898998 | 13922768 | 0.05 | 0.4 |
| CIS | uc003mui.1 |  | SERPINB6 | 6 | 6p25 | 2893391 | 2901115 | 0.05 | 0.4 |
| CIS, SCC | uc003piz.1 | NM_031469 | SH3BGRL2 | 6 | 6q13 | 80397718 | 80470088 | 0 | 0.4 |
| CIS, SCC | uc003str.1 | NM_006408 | AGR2 | 7 | 7p21.3 | 16797959 | 16811133 | 0.15 | 0.1 |
| CIS | uc003sqe.1 | NM_006854 | KDELR2 | 7 | 7p22.1 | 6468794 | 6490225 | 0.15 | 0.05 |
| CIS | uc003tzg.1 | NM_001306 | CLDN3 | 7 | 7q11.23 | 72821262 | 72822512 | 0.5 | 0.2 |
| CIS | uc003ujs.1 | NM_024636 | STEAP4 | 7 | 7q21.12 | 87743679 | 87774145 | 0.2 | 0.05 |
| CIS | uc003xft.1 | NM_000742 | CHRNA2 | 8 | 8p21 | 27374181 | 27392675 | 0.1 | 0.5 |
| CIS | uc003ywu.1 | NM_005672 | PSCA | 8 | 8q24.2 | 143758876 | 143761145 | 0.6 | 0.05 |
| CIS, SCC | uc003ywz.1 | NM_205545 | LYPD2 | 8 | 8q24.3 | 143828629 | 143830954 | 0.6 | 0.05 |
| CIS | uc003zsv.1 |  | AQP3 | 9 | 9p13 | 33431153 | 33437590 | 0.1 | 0.6 |
| CIS | uc003zzg.1 | NM_005476 | GNE | 9 | 9p13.3 | 36204438 | 36248401 | 0.25 | 0.55 |
| CIS | uc004bzm.1 | NM_000050 | ASS1 | 9 | 9q34.1 | 132309914 | 132366482 | 0.3 | 0.1 |
| SCC | uc001ink.1 | NM_001033858 | DCLRE1C | 10 | 10p13 | 14988876 | 15005680 | 0 | 0.3 |
| CIS | uc001jpo.1 | NM_012339 | TSPAN15 | 10 | 10q21.3 | 70881231 | 70937429 | 0 | 0.2 |
| CIS | uc001kbq.1 | NM_145869 | ANXA11 | 10 | 10q23 | 81904859 | 81954227 | 0 | 0.2 |
| SCC | uc001mmm.1 |  | PLEKHA7 | 11 | 11p15.1 | 16765785 | 16769805 | 0 | 0.4 |
| CIS, SCC | uc001lru.1 | NM_173584 | EFCAB4A | 11 | 11p15.5 | 817617 | 821986 | 0.45 | 0.15 |
| CIS, SCC | uc001ntj.1 | NM_003357 | SCGB1A1 | 11 | 11q12.3-13.1 | 61943098 | 61947243 | 0.15 | 0.15 |
| CIS | uc001ogr.1 | NM_001323 | CST6 | 11 | 11q13 | 65536037 | 65537552 | 0.25 | 0.15 |
| CIS, SCC | uc001ogb.1 |  | CTSW | 11 | 11q13.1 | 65403859 | 65407726 | 0.25 | 0.15 |
| CIS | uc001pyo.1 | NM_006597 | HSPA8 | 11 | 11q24.1 | 122433409 | 122438054 | 0 | 0.35 |
| CIS | uc001saa.1 | NM_005556 | KRT7 | 12 | 12q12 | 50913220 | 50928976 | 0 | 0.2 |
| CIS, SCC | uc001rvo.1 | NM_001651 | AQP5 | 12 | 12q13 | 48641545 | 48645227 | 0 | 0.15 |
| SCC | uc001vjs.1 | NM_005358 | LMO7 | 13 | 13q22.2 | 75092570 | 75332003 | 0 | 0.6 |
| CIS | uc001vqy.1 | NM_017817 | RAB20 | 13 | 13q34 | 109973413 | 110012072 | 0.05 | 0.7 |
| CIS | uc001xbr.1 | NM_002306 | LGALS3 | 14 | 14q21 | 54665624 | 54681901 | 0.05 | 0.35 |
| CIS | uc001ycw.1 |  | SERPINA1 | 14 | 14q32.1 | 93914450 | 93919331 | 0.05 | 0.35 |
| CIS | uc001yeg.1 | NM_024734 | CLMN | 14 | 14q32.13 | 94727617 | 94855955 | 0.1 | 0.35 |
| CIS, SCC | uc001yrc.1 |  | CRIP2 | 14 | 14q32.3 | 105010809 | 105016429 | 0.4 | 0.15 |
| CIS, SCC | uc001yri.1 | NM_001311 | CRIP1 | 14 | 14q32.33 | 105024593 | 105026169 | 0.35 | 0.2 |
| CIS | uc001yus.1 | NM_014608 | CYFIP1 | 15 | 15q11 | 20444124 | 20555044 | 0.05 | 0.65 |
| CIS, SCC | uc001zhp.1 | NM_024713 | C15orf29 | 15 | 15q14 | 32220166 | 32289515 | 0.1 | 0.2 |
| CIS, SCC | uc001zus.1 | NM_017434 | DUOX1 | 15 | 15q15.3 | 43209483 | 43245066 | 0.15 | 0.25 |
| CIS, SCC | uc002abw.1 | NM_018728 | MYO5C | 15 | 15q21 | 50271811 | 50375144 | 0.1 | 0.2 |
| CIS | uc002gym.1 |  | USP22 | 17 | 17p11.2 | 20844852 | 20886944 | 0 | 0.85 |
| CIS | uc002hsm.1 | NM_001005862 | ERBB2 | 17 | 17q21.1 | 35097918 | 35138441 | 0.3 | 0.1 |
| CIS | uc002jsh.1 | NM_018414 | ST6GALNAC1 | 17 | 17q25.1 | 72132439 | 72151489 | 0.6 | 0.05 |
| SCC | uc002kdx.1 | NM_198082 | CCDC57 | 17 | 17q25.3 | 77652634 | 77763978 | 0.6 | 0.1 |
| CIS, SCC | uc002ljj.1 |  | SERPINB11 | 18 | 18 | 59528381 | 59541613 | 0.15 | 0.3 |
| CIS | uc002lfg.1 | NM_003927 | MBD2 | 18 | 18q21 | 49934572 | 50005156 | 0.1 | 0.35 |
| CIS | uc002niy.1 | NM_032627 | SSBP4 | 19 | 19p13.1 | 18391135 | 18406459 | 0.45 | 0.15 |
| CIS, SCC | uc002net.1 | NM_001007525 | NWD1 | 19 | 19p13.11 | 16691786 | 16789763 | 0.3 | 0.15 |
| CIS, SCC | uc002lyk.1 | NM_014428 | TJP3 | 19 | 19p13.3 | 3659381 | 3701682 | 0.5 | 0.05 |
| SCC | uc002orj.1 | NM_004363 | CEACAM5 | 19 | 19q13.1 | 46904369 | 46923112 | 0.25 | 0.25 |
| CIS | uc002orm.1 | NM_002483 | CEACAM6 | 19 | 19q13.2 | 46951340 | 46967953 | 0.25 | 0.25 |
| CIS | uc002pgj.1 | NM_001736 | C5AR1 | 19 | 19q13.3 | 52504943 | 52517167 | 0.2 | 0.25 |
| CIS | uc002xnu.1 | NM_002999 | SDC4 | 20 | 20q12 | 43387342 | 43410478 | 0.2 | 0.1 |
| CIS | uc002xon.1 | NM_080736 | WFDC2 | 20 | 20q12-13.2 | 43531807 | 43541802 | 0.2 | 0.1 |
| CIS | uc002xyj.1 | NM_017495 | RBM38 | 20 | 20q13.31 | 55399869 | 55417792 | 0.05 | 0.15 |
| CIS | uc002yuf.1 | NM_053277 | CLIC6 | 21 | 21q22 | 34963557 | 35012389 | 0.1 | 0.4 |
| CIS | uc002yzj.1 | NM_005656 | TMPRSS2 | 21 | 21q22.3 | 41758350 | 41801948 | 0.1 | 0.4 |

^1^Relative to BE and PC.

^2^Sorted according to chromosomal locus.

^3^Transcription start; ^4^Transcription end. ^5^Average frequency from 20 independent CIS specimens.
