# Supplementary material for: Transcriptome Profiles of Carcinoma-in-Situ and Invasive Non-Small Cell Lung Cancer as Revealed by SAGE
Source: PLoS One. 2010 Feb 11;5(2):e9162. doi: 10.1371/journal.pone.0009162 (PMC2820080; doi:10.1371/journal.pone.0009162)
Supplement: Text S1 — Further description of the down-regulated genes displayed in Figure S2. (0.14 MB DOC) [file pone.0009162.s020.doc]

Genes down-regulated in both CIS and PC datasets relative to BE, were analyzed by IPA pathway graphical representation; only those gene products connected by direct or indirect interactions were included in Figure S2. Transcription factors identified here include mediators of mucociliary differentiation FOXJ1 (see main manuscript), and SPDEF, a master regulator of goblet cell development [1,2]. Additionally, FOXJ1, as well as transcription factor CITED2, are developmental regulators of left/right asymmetry determination [3,4,5]. The most prominent network in Figure S2 displays interactions among heavy, intermediate, and light chains of ciliary axonemal dyneins. Additional axonemal constituents identified here include IFT proteins that mediate intraciliary transport required for assembly and maintenance of primary cilia [6,7], NPHP1 associated with the hereditary cystic kidney disease nephronophthisis [8], and proteins associated with spermatogenesis including SPAG6, SPAG16, and nucleoside diphosphate kinase NME5 [9,10,11,12]. KIF3A and KIF3B, motor components of kinesin-2, mediate microtubule-directed anterograde transport along the ciliary axoneme, and are required for both maintenance of cilia and left/right asymmetry [13,14,15]. Chaperone proteins HSPH1 and HSP90 stabilize tubulin polymerization and ciliary beating [16]. Mucosal gene products identified in Figure S2 include the Clara cell globular protein SCGB1A1, MUC5AC the major mucin of the airway surface liquid (ASL), surfactant/surfactant processing proteins, and TFF3 associated with mucosal restitution including ciliogenesis [17,18,19]. TGFA contributes to goblet cell hyperplasia via induction of MUC5AC gene expression in bronchial epithelium in response to stress stimuli (dsRNA, virus, oxidative stress) via EGFR signaling [20]; [21,22,23], and to mucous cell metaplasia in response to IL-13 [24,25]. Other genes in Figure S2 associated with host defense/detoxification/anti-oxidant defense include secreted protease inhibitor SLPI [26], major histocompatibility gene HLA-B, aldo-keto reductase AKR7A2, oxido-reductase SOD1, and major anti-oxidant enzyme, CAT.

Considering the structural similarity between centrosomes and ciliary basal bodies, it is not surprising to find genes encoding centrosomal proteins to be identified in Figure S2. Cyclin-dependent kinase CDK2, microtubule polymerization, cytoplasmic dynein, and HSP90 (all represented in Figure S2), play a role in over-duplication of centrosomes and formation of centriolar satellites with consequences for both cytokinesis and ciliogenesis [27]. Additional centrosomal proteins identified here include protein kinase A anchoring protein AKAP9, a mediator of microtubular nucleation, and centrosome duplication/amplification [28,29,30], ADP-ribosylation factor ARL3, associated with acetylation of alpha-tubulin and cytokinesis, and also found in photoreceptor connecting cilium [31,32,33], and DNA helicase RUVBL1. Although p53 was not identified within the down-regulated genes in PC and CIS lesions, the detection of functionally related genes such as MLF1, an inducer of p53 [34], and genes associated with DNA damage response [CDK2-activator protein SPDYA, ATM/53BP1-regulated S-phase checkpoint gene RIF1 [35], DNA helicase RUVBL2], suggests that the inhibition of centrosome over-duplication by p53 [36,37], may be important for maintenance of the mutliciliated bronchial epithelium. Identification of these genes likely reflects the tight regulation that links DNA damage-induced cell cycle checkpoint signaling and centrosome homeostasis [38,39].

Differentiated bronchial epithelial cells are highly polarized, and genes regulating epithelial cell polarization including cytoskeletal linker protein EZR and apical scaffolding protein SPTA1, are identified in Figure S2. FOXJ1 is required for apical localization of EZR in ciliated airway cells, and for EZR-mediated anchoring of basal bodies to the apical cytoskeleton for axoneme formation [40,41]. EZR mediates apical recruitment of additional proteins, including the sodium/hydrogen exchanger NHE3 to the plasma membrane of intestinal brush border cells via interaction with SLC9A3R2/NHERF2, also identified in Figure S2 [42]. Regulation of transepithelial ion transport is crucial to maintain proper hydration and volume of the ASL, critical for mucociliary clearance [43,44,45,46].

**References**

1. Yu X, Ng CP, Habacher H, Roy S (2008) Foxj1 transcription factors are master regulators of the motile ciliogenic program. Nat Genet 40: 1445-1453.

2. Chen G, Korfhagen TR, Xu Y, Kitzmiller J, Wert SE, et al. (2009) SPDEF is required for mouse pulmonary goblet cell differentiation and regulates a network of genes associated with mucus production. J Clin Invest 119: 2914-2924.

3. Weninger WJ, Lopes Floro K, Bennett MB, Withington SL, Preis JI, et al. (2005) Cited2 is required both for heart morphogenesis and establishment of the left-right axis in mouse development. Development 132: 1337-1348.

4. Bamforth SD, Braganca J, Farthing CR, Schneider JE, Broadbent C, et al. (2004) Cited2 controls left-right patterning and heart development through a Nodal-Pitx2c pathway. Nat Genet 36: 1189-1196.

5. Chen J, Knowles HJ, Hebert JL, Hackett BP (1998) Mutation of the mouse hepatocyte nuclear factor/forkhead homologue 4 gene results in an absence of cilia and random left-right asymmetry. J Clin Invest 102: 1077-1082.

6. Cole DG (2003) The intraflagellar transport machinery of Chlamydomonas reinhardtii. Traffic 4: 435-442.

7. Pazour GJ, Dickert BL, Vucica Y, Seeley ES, Rosenbaum JL, et al. (2000) Chlamydomonas IFT88 and its mouse homologue, polycystic kidney disease gene tg737, are required for assembly of cilia and flagella. J Cell Biol 151: 709-718.

8. Fliegauf M, Horvath J, von Schnakenburg C, Olbrich H, Muller D, et al. (2006) Nephrocystin specifically localizes to the transition zone of renal and respiratory cilia and photoreceptor connecting cilia. J Am Soc Nephrol 17: 2424-2433.

9. Zhang Z, Sapiro R, Kapfhamer D, Bucan M, Bray J, et al. (2002) A sperm-associated WD repeat protein orthologous to Chlamydomonas PF20 associates with Spag6, the mammalian orthologue of Chlamydomonas PF16. Mol Cell Biol 22: 7993-8004.

10. Zhang Z, Zariwala MA, Mahadevan MM, Caballero-Campo P, Shen X, et al. (2007) A heterozygous mutation disrupting the SPAG16 gene results in biochemical instability of central apparatus components of the human sperm axoneme. Biol Reprod 77: 864-871.

11. Munier A, Serres C, Kann ML, Boissan M, Lesaffre C, et al. (2003) Nm23/NDP kinases in human male germ cells: role in spermiogenesis and sperm motility? Exp Cell Res 289: 295-306.

12. Ross AJ, Dailey LA, Brighton LE, Devlin RB (2007) Transcriptional profiling of mucociliary differentiation in human airway epithelial cells. Am J Respir Cell Mol Biol 37: 169-185.

13. Lin F, Hiesberger T, Cordes K, Sinclair AM, Goldstein LS, et al. (2003) Kidney-specific inactivation of the KIF3A subunit of kinesin-II inhibits renal ciliogenesis and produces polycystic kidney disease. Proc Natl Acad Sci U S A 100: 5286-5291.

14. Marszalek JR, Ruiz-Lozano P, Roberts E, Chien KR, Goldstein LS (1999) Situs inversus and embryonic ciliary morphogenesis defects in mouse mutants lacking the KIF3A subunit of kinesin-II. Proc Natl Acad Sci U S A 96: 5043-5048.

15. Nonaka S, Tanaka Y, Okada Y, Takeda S, Harada A, et al. (1998) Randomization of left-right asymmetry due to loss of nodal cilia generating leftward flow of extraembryonic fluid in mice lacking KIF3B motor protein. Cell 95: 829-837.

16. Takaki E, Fujimoto M, Nakahari T, Yonemura S, Miyata Y, et al. (2007) Heat shock transcription factor 1 is required for maintenance of ciliary beating in mice. J Biol Chem 282: 37285-37292.

17. Kjellev S (2009) The trefoil factor family - small peptides with multiple functionalities. Cell Mol Life Sci 66: 1350-1369.

18. Hoffmann W (2005) Trefoil factors TFF (trefoil factor family) peptide-triggered signals promoting mucosal restitution. Cell Mol Life Sci 62: 2932-2938.

19. LeSimple P, van Seuningen I, Buisine MP, Copin MC, Hinz M, et al. (2007) Trefoil factor family 3 peptide promotes human airway epithelial ciliated cell differentiation. Am J Respir Cell Mol Biol 36: 296-303.

20. Takeyama K, Dabbagh K, Lee HM, Agusti C, Lausier JA, et al. (1999) Epidermal growth factor system regulates mucin production in airways. Proc Natl Acad Sci U S A 96: 3081-3086.

21. Tadaki H, Arakawa H, Mizuno T, Suzuki T, Takeyama K, et al. (2009) Double-stranded RNA and TGF-alpha promote MUC5AC induction in respiratory cells. J Immunol 182: 293-300.

22. Zhu L, Lee PK, Lee WM, Zhao Y, Yu D, et al. (2009) Rhinovirus-induced major airway mucin production involves a novel TLR3-EGFR-dependent pathway. Am J Respir Cell Mol Biol 40: 610-619.

23. Yan F, Li W, Jono H, Li Q, Zhang S, et al. (2008) Reactive oxygen species regulate Pseudomonas aeruginosa lipopolysaccharide-induced MUC5AC mucin expression via PKC-NADPH oxidase-ROS-TGF-alpha signaling pathways in human airway epithelial cells. Biochem Biophys Res Commun 366: 513-519.

24. Tesfaigzi Y (2008) Regulation of mucous cell metaplasia in bronchial asthma. Curr Mol Med 8: 408-415.

25. Booth BW, Sandifer T, Martin EL, Martin LD (2007) IL-13-induced proliferation of airway epithelial cells: mediation by intracellular growth factor mobilization and ADAM17. Respir Res 8: 51.

26. Weldon S, Taggart CC (2007) Innate host defense functions of secretory leucoprotease inhibitor. Exp Lung Res 33: 485-491.

27. Prosser SL, Straatman KR, Fry AM (2009) Molecular dissection of the centrosome overduplication pathway in S-phase-arrested cells. Mol Cell Biol 29: 1760-1773.

28. Larocca MC, Jin M, Goldenring JR (2006) AKAP350 modulates microtubule dynamics. Eur J Cell Biol 85: 611-619.

29. Rivero S, Cardenas J, Bornens M, Rios RM (2009) Microtubule nucleation at the cis-side of the Golgi apparatus requires AKAP450 and GM130. EMBO J 28: 1016-1028.

30. Nishimura T, Takahashi M, Kim HS, Mukai H, Ono Y (2005) Centrosome-targeting region of CG-NAP causes centrosome amplification by recruiting cyclin E-cdk2 complex. Genes Cells 10: 75-86.

31. Zhou C, Cunningham L, Marcus AI, Li Y, Kahn RA (2006) Arl2 and Arl3 regulate different microtubule-dependent processes. Mol Biol Cell 17: 2476-2487.

32. Grayson C, Bartolini F, Chapple JP, Willison KR, Bhamidipati A, et al. (2002) Localization in the human retina of the X-linked retinitis pigmentosa protein RP2, its homologue cofactor C and the RP2 interacting protein Arl3. Hum Mol Genet 11: 3065-3074.

33. Schrick JJ, Vogel P, Abuin A, Hampton B, Rice DS (2006) ADP-ribosylation factor-like 3 is involved in kidney and photoreceptor development. Am J Pathol 168: 1288-1298.

34. Yoneda-Kato N, Tomoda K, Umehara M, Arata Y, Kato JY (2005) Myeloid leukemia factor 1 regulates p53 by suppressing COP1 via COP9 signalosome subunit 3. EMBO J 24: 1739-1749.

35. Silverman J, Takai H, Buonomo SB, Eisenhaber F, de Lange T (2004) Human Rif1, ortholog of a yeast telomeric protein, is regulated by ATM and 53BP1 and functions in the S-phase checkpoint. Genes Dev 18: 2108-2119.

36. Tarapore P, Fukasawa K (2002) Loss of p53 and centrosome hyperamplification. Oncogene 21: 6234-6240.

37. Fukasawa K (2008) P53, cyclin-dependent kinase and abnormal amplification of centrosomes. Biochim Biophys Acta 1786: 15-23.

38. Fletcher L, Muschel RJ (2006) The centrosome and the DNA damage induced checkpoint. Cancer Lett 243: 1-8.

39. Gong Y, Sun Y, McNutt MA, Sun Q, Hou L, et al. (2009) Localization of TEIF in the centrosome and its functional association with centrosome amplification in DNA damage, telomere dysfunction and human cancers. Oncogene 28: 1549-1560.

40. Gomperts BN, Gong-Cooper X, Hackett BP (2004) Foxj1 regulates basal body anchoring to the cytoskeleton of ciliated pulmonary epithelial cells. J Cell Sci 117: 1329-1337.

41. Huang T, You Y, Spoor MS, Richer EJ, Kudva VV, et al. (2003) Foxj1 is required for apical localization of ezrin in airway epithelial cells. J Cell Sci 116: 4935-4945.

42. Cha B, Donowitz M (2008) The epithelial brush border Na+/H+ exchanger NHE3 associates with the actin cytoskeleton by binding to ezrin directly and via PDZ domain-containing Na+/H+ exchanger regulatory factor (NHERF) proteins. Clin Exp Pharmacol Physiol 35: 863-871.

43. Randell SH, Boucher RC (2006) Effective mucus clearance is essential for respiratory health. Am J Respir Cell Mol Biol 35: 20-28.

44. Tarran R, Trout L, Donaldson SH, Boucher RC (2006) Soluble mediators, not cilia, determine airway surface liquid volume in normal and cystic fibrosis superficial airway epithelia. J Gen Physiol 127: 591-604.

45. Tarran R (2004) Regulation of airway surface liquid volume and mucus transport by active ion transport. Proc Am Thorac Soc 1: 42-46.

46. Tarran R, Grubb BR, Gatzy JT, Davis CW, Boucher RC (2001) The relative roles of passive surface forces and active ion transport in the modulation of airway surface liquid volume and composition. J Gen Physiol 118: 223-236.
