# Supplementary material for: Transcriptome Profiles of Carcinoma-in-Situ and Invasive Non-Small Cell Lung Cancer as Revealed by SAGE
Source: PLoS One. 2010 Feb 11;5(2):e9162. doi: 10.1371/journal.pone.0009162 (PMC2820080; doi:10.1371/journal.pone.0009162)
Supplement: Text S2 — Further description of the down-regulated genes displayed in Figure S4. (0.08 MB DOC) [file pone.0009162.s021.doc]

Gene products down-regulated in CIS relative to BE and PC, and in SCC relative to BE and PC, were displayed by IPA pathway graphical representation, according to both direct and indirect connections (Figure S4). ERBB2, receptor tyrosine kinase of the EGFR family, is central to the most prominent interactive network identified here. ERBB2 is associated with multiple developmental processes, but particularly relevant for the discussion here, are reports that ERBB2 plays a role in airway repair, including differentiation of ciliated and goblet cells, while inhibiting squamous metaplasia [1]. Interacting proteins identified here include transmembrane mucins MUC4 and MUC1. MUC4 has been identified as a functional ligand for ERBB2 in response to mechanical injury, mediating localization to the apical membrane, phosphorylation, and down-stream signaling [2]. MUC1 also associates with erbB receptors to mediate MAP kinase signaling [3]. The molecular connections identified in Figure S4 suggests that EZR, activated by S100P in response to calcium stimulation [4], and tight junction protein CLDN3 [5], may contribute to apical localization of ERBB2 in airway epithelium. Other genes forming connections with ERBB2 in Figure S4 include disulfide isomerase AGR2, associated with goblet cell differentiation and mucus production/processing [6,7], KRT7, cytoskeletal protein and goblet cell marker [8], and two LIM domain proteins, CRIP1 and PDLIM4, associated with cytoskeletal actin remodeling [9]. It is intriguing to hypothesize that failure to initiate a potential ERBB2 signaling complex in CIS lesions, may compromise re-differentiation/restoration of the bronchial epithelium following injury, and contribute to initiation of in-situ cancer.

It is interesting to note that we observe down-regulation of alpha 2 nAChR (CHRNA2) in the CIS dataset, accompanied by relatively high expression in PC lesions relative to both CIS and BE (Table S9). A recent study demonstrates a role for the alpha 7 subunit of nAChR in airway epithelial differentiation, via inhibition of basal cell proliferation and squamous metaplasia [10]. It would be of value to determine whether or not alpha 2 also exerts this regulation over basal cell proliferation during initiation of in-situ cancer. Intriguingly, the ERBB2 pathway induces expression of nAChR subunits [11,12].

We observe down-regulation of protease inhibitor CST6 in CIS lesions relative to both BE and PC. Through inhibition of cathepsin protease-mediated processing of transglutaminase cross-linking enzymes, CST6 is associated with regulation of epidermal terminal differentiation and barrier formation [13,14]. Consideration that expression of CST6 is frequently down-regulated by promoter hypermethylation in various cancers including breast [15], and that numerous genes within the epidermal terminal differentiation pathway are up-regulated in lung CIS lesions (see main manuscript), warrants further investigation into the role of this pathway in lung cancer development.

IPA analysis previously identified a number of genes associated with DNA replication/repair to be underexpressed in both CIS and PC lesions relative to BE (see main manuscript). Genes associated with DNA repair/telomerase maintenance that are under-expressed in CIS/SCC relative to PC lesions (and BE) include nuclease DCLRE1C (Artemis) [16,17], USP22, ubiquitin-specific peptidase and component of the SAGA complex [18], MBD2, a methyl-CpG binding transcriptional regulator and repressor of hTERT transcription [19], and nuclear orphan receptor NR4A2 [20].

Additional down-regulated genes in Figure S4 specify proteins associated with physiology and function of the bronchial epithelium. These include proteins associated with cell polarity, including tight junction protein (TJP3/ZO-3), and adherens junction proteins (LMO7 and PLEKHA7), water channel proteins (AQP3 and AQP5), ion channel protein (CLIC6), genes associated with mucin production and modification (ST6GALNAC1, DUOX1), genes associated with host defense (WFDC2), and genes associated with retinoic acid metabolism and signaling (ADH7, DHRS9, NR4A2). Other down-regulated genes identified in Figure S4 include molecules associated with immune response (C5AR1, CXCL3, TNFRSF14), actin-binding/regulatory proteins (CLMN, CYFIP1, PLS1, VILL, MYO5C, MLPH, CRIP2), intracellular transport/targeting proteins (TRAK1, MYO5C, MLPH, RAB20), protein folding and modification proteins (EDEM3, HSPA8, SUMF1, KDELR2, GNE, SUMF1, UBA1), and transcription factors/regulators (TRAK1, NR4A2, CASZ1, TCEA3, SLC30A9, SSBP4).

**References**

1. Vermeer PD, Panko L, Karp P, Lee JH, Zabner J (2006) Differentiation of human airway epithelia is dependent on erbB2. Am J Physiol Lung Cell Mol Physiol 291: L175-180.

2. Theodoropoulos G, Carraway CA, Carraway KL (2009) MUC4 involvement in ErbB2/ErbB3 phosphorylation and signaling in response to airway cell mechanical injury. J Cell Biochem 107: 112-122.

3. Schroeder JA, Thompson MC, Gardner MM, Gendler SJ (2001) Transgenic MUC1 interacts with epidermal growth factor receptor and correlates with mitogen-activated protein kinase activation in the mouse mammary gland. J Biol Chem 276: 13057-13064.

4. Koltzscher M, Neumann C, Konig S, Gerke V (2003) Ca2+-dependent binding and activation of dormant ezrin by dimeric S100P. Mol Biol Cell 14: 2372-2384.

5. Shin K, Fogg VC, Margolis B (2006) Tight junctions and cell polarity. Annu Rev Cell Dev Biol 22: 207-235.

6. Chen G, Korfhagen TR, Xu Y, Kitzmiller J, Wert SE, et al. (2009) SPDEF is required for mouse pulmonary goblet cell differentiation and regulates a network of genes associated with mucus production. J Clin Invest 119: 2914-2924.

7. Park SW, Zhen G, Verhaeghe C, Nakagami Y, Nguyenvu LT, et al. (2009) The protein disulfide isomerase AGR2 is essential for production of intestinal mucus. Proc Natl Acad Sci U S A 106: 6950-6955.

8. Shatos MA, Rios JD, Tepavcevic V, Kano H, Hodges R, et al. (2001) Isolation, characterization, and propagation of rat conjunctival goblet cells in vitro. Invest Ophthalmol Vis Sci 42: 1455-1464.

9. Vallenius T, Scharm B, Vesikansa A, Luukko K, Schafer R, et al. (2004) The PDZ-LIM protein RIL modulates actin stress fiber turnover and enhances the association of alpha-actinin with F-actin. Exp Cell Res 293: 117-128.

10. Maouche K, Polette M, Jolly T, Medjber K, Cloez-Tayarani I, et al. (2009) {alpha}7 nicotinic acetylcholine receptor regulates airway epithelium differentiation by controlling basal cell proliferation. Am J Pathol 175: 1868-1882.

11. Yarden Y, Sliwkowski MX (2001) Untangling the ErbB signalling network. Nat Rev Mol Cell Biol 2: 127-137.

12. Falls DL, Rosen KM, Corfas G, Lane WS, Fischbach GD (1993) ARIA, a protein that stimulates acetylcholine receptor synthesis, is a member of the neu ligand family. Cell 72: 801-815.

13. Zeeuwen PL, Cheng T, Schalkwijk J (2009) The biology of cystatin M/E and its cognate target proteases. J Invest Dermatol 129: 1327-1338.

14. Cheng T, van Vlijmen-Willems IM, Hitomi K, Pasch MC, van Erp PE, et al. (2009) Colocalization of cystatin M/E and its target proteases suggests a role in terminal differentiation of human hair follicle and nail. J Invest Dermatol 129: 1232-1242.

15. Schagdarsurengin U, Pfeifer GP, Dammann R (2007) Frequent epigenetic inactivation of cystatin M in breast carcinoma. Oncogene 26: 3089-3094.

16. Frias C, Garcia-Aranda C, De Juan C, Moran A, Ortega P, et al. (2008) Telomere shortening is associated with poor prognosis and telomerase activity correlates with DNA repair impairment in non-small cell lung cancer. Lung Cancer 60: 416-425.

17. Rooney S, Alt FW, Lombard D, Whitlow S, Eckersdorff M, et al. (2003) Defective DNA repair and increased genomic instability in Artemis-deficient murine cells. J Exp Med 197: 553-565.

18. Atanassov BS, Evrard YA, Multani AS, Zhang Z, Tora L, et al. (2009) Gcn5 and SAGA regulate shelterin protein turnover and telomere maintenance. Mol Cell 35: 352-364.

19. Chatagnon A, Bougel S, Perriaud L, Lachuer J, Benhattar J, et al. (2009) Specific association between the methyl-CpG-binding domain protein 2 and the hypermethylated region of the human telomerase reverse transcriptase promoter in cancer cells. Carcinogenesis 30: 28-34.

20. Smith AG, Luk N, Newton RA, Roberts DW, Sturm RA, et al. (2008) Melanocortin-1 receptor signaling markedly induces the expression of the NR4A nuclear receptor subgroup in melanocytic cells. J Biol Chem 283: 12564-12570.
